# Theoretical Models of Consciousness: A Scoping Review

**DOI:** 10.3390/brainsci11050535

**Published:** 2021-04-24

**Authors:** Davide Sattin, Francesca Giulia Magnani, Laura Bartesaghi, Milena Caputo, Andrea Veronica Fittipaldo, Martina Cacciatore, Mario Picozzi, Matilde Leonardi

**Affiliations:** 1 Neurology, Public Health, Disability Unit—Scientific Department, Fondazione IRCCS Istituto Neurologico Carlo Besta, 20133 Milan, Italy; francesca.magnani@istituto-besta.it (F.G.M.); bartesaghilaura@gmail.com (L.B.); milena.caputo@outlook.com (M.C.); marty9484@live.it (M.C.); matilde.leonardi@istituto-besta.it (M.L.); 2 Experimental Medicine and Medical Humanities-PhD Program, Biotechnology and Life Sciences Department and Center for Clinical Ethics, Insubria University, 21100 Varese, Italy; 3 Oncology Department, Mario Negri Institute for Pharmacological Research IRCCS, 20156 Milan, Italy; veronicaandrea.fittipaldo@marionegri.it; 4 Center for Clinical Ethics, Biotechnology and Life Sciences Department, Insubria University, 21100 Varese, Italy; mario.picozzi@uninsubria.it

**Keywords:** consciousness, neural correlates, cognition, consciousness theory, consciousness definition, subjectivity

## Abstract

The amount of knowledge on human consciousness has created a multitude of viewpoints and it is difficult to compare and synthesize all the recent scientific perspectives. Indeed, there are many definitions of consciousness and multiple approaches to study the neural correlates of consciousness (NCC). Therefore, the main aim of this article is to collect data on the various theories of consciousness published between 2007–2017 and to synthesize them to provide a general overview of this topic. To describe each theory, we developed a thematic grid called the dimensional model, which qualitatively and quantitatively analyzes how each article, related to one specific theory, debates/analyzes a specific issue. Among the 1130 articles assessed, 85 full texts were included in the prefinal step. Finally, this scoping review analyzed 68 articles that described 29 theories of consciousness. We found heterogeneous perspectives in the theories analyzed. Those with the highest grade of variability are as follows: subjectivity, NCC, and the consciousness/cognitive function. Among sub-cortical structures, thalamus, basal ganglia, and the hippocampus were the most indicated, whereas the cingulate, prefrontal, and temporal areas were the most reported for cortical ones also including the thalamo-cortical system. Moreover, we found several definitions of consciousness and 21 new sub-classifications.

## 1. Introduction

The scientific literature debating themes on consciousness is huge. Contributing scientists come from different disciplines such as medicine, philosophy, physics, psychology, neurobiology, mathematics, and computer science. Authors from these disciplines have led to an increase in knowledge about consciousness, giving new and interesting inputs for neuroscience. However, all this information has created a multitude of viewpoints and it is difficult to compare (and synthesize) some of the various issues that have emerged in the last few decades. For example, one of the main problems related to consciousness is its definition. Indeed, as remarked by Sommerhoff [1], “A precise definition of the word [‘consciousness’], of course, can only be the endpoint of a theory of consciousness, just as the concepts of work and energy found a precise definition only as part of a theory of mechanics” [1]. Therefore, the probability of having different definitions of the same object correlates directly to the number of theories that try to explain the nature of consciousness itself. In this sense, the concept of ‘consciousness’ can be found in historical documents since ancient times, and it can be proven in various traditions and cultures with some perspectives that, although the expression language has become different, seem to be concepts that had evolved throughout history [2]. Fritjof Capra [3] describes how some eastern ancient concepts on a universal consciousness are consistent with modern physics, considering that some physics perspectives try to invalidate the concept of atomic particles as consisting of smaller independent building blocks made of different substances. Capra believes that one substance exists that comes from a ‘cosmic consciousness’ (this was the definition of consciousness used) out of which all matter is made of. This is a position commonly used also by those who argue that consciousness supervenes on the whole embodied animal in dynamic interaction with the environment [4]. Another example proposed in the last decade derived by some eastern reductionist perspective highlighted that consciousness could be defined as “the ability to maintain an alert state, attention, and awareness of self and environment” [5] or as the “the subjective character of our mental states” [6], according to the clinical and representational perspective used to describe it. This provides particular attention to the role of introspection and metarepresentation, two themes cited by Aristotle that contribute to current debates over the nature of consciousness [7].

Therefore, the analysis of the general framework and the perspectives proposed by each model of consciousness is important for improving the science of consciousness and, consequently, its definitions.

Another issue is represented by the definition of the neural correlates of consciousness (NCCs). If we consider consciousness as a human phenomenon deriving from matter, one of the main neurobiological questions is the definition of the minimum neuronal mechanisms jointly sufficient for any one specific conscious percept [8,9]. The definition of what we mean by consciousness and the brain neurons/areas/processes linked to the appearance of this phenomenon is a crucial issue for all theories of consciousness. The evolution of imaging and neurophysiological techniques, as well as the possibility to change brain activities online using tailored magnetic and electrical stimulations during tasks requiring wakefulness and awareness of external stimuli, has led to increases in the possibility of verifying/falsifying different hypotheses. Moreover, it has led to study the of consciousness modulation through the stimulation of different brain areas, overpassing the limits derived from the anatomo-clinical model based on the lesional approach. However, every theoretical approach has some assumptions according to which consciousness could be generated by a multitude of processes. This is grounded in the activity of a wide range of structures that vary from the single neuron to the whole brain. Consequently, the number of NCCs is presently very large.

There are other main issues debated in the scientific literature related to consciousness. The problem of how we can “quantify” the conscious level, for example, is one of the most important questions that many scientists are still studying. This question is crucial for clinicians during the clinical diagnosis of patients with disorders of consciousness after a severe brain injury (to assess the consciousness level beyond behavioral abilities of patients), as well as for computer science/artificial intelligence researchers involved in machine and computer development.

All the examples reported above makeup only a minimal part of the literature on consciousness, which has increased over the last few years. Therefore, the general aim of this article is to offer an exhaustive framework on the theoretical models of consciousness that currently exist, without committing to a specific perspective or viewpoint. To achieve this goal, the specific aims of this research are twofold: Firstly, we sought to systematically review the scientific literature on theoretical models of consciousness published from 2007 to 2017 in major scientific databases and, secondly, to describe each theory collected using a common thematic grid called the dimensional model (DM) in order to qualitatively and quantitatively analyze how each article, related to a specific theory, debates/analyzes specific issues.

## 2. Materials and Methods

We included all the published studies that analyzed theoretical aspects of consciousness.

### 2.1. Search Strategies and Information Sources

The authors performed a systematic search in the Medline (PubMed), Embase (EMBASE.com), Scopus, Web of Science (WoS), and PsycINFO databases. A tailored search strategy was developed for each database according to their thesaurus characteristics. The search method for the identification of studies is reported in Supplemental Materials (Table S1). Results obtained from each database were exported in a web-based bibliographic management software (RefWorks; https://www.refworks.com/it/, accessed on May–December 2017) and duplicate deletion was done by two independent raters, which were then matched. Moreover, all records were imported in a tailored Excel spreadsheet including title, abstract, and record information for each article.

### 2.2. General Selection Criteria

Studies were excluded if they met any of the following criteria: (1) studies published before 2007; (2) article type: book, generic, magazine article, or monograph; (3) studies not published in the English or Italian languages.

This review was performed following the preferred reporting items for systematic reviews and meta-analyses criteria [10].

### 2.3. Screening and Eligibility Criteria for Each Step

Search results were independently screened by different reviewers using a three-step procedure due to the high number of articles selected. Duplicate articles were removed before step 1.

#### 2.3.1. Step 1: Abstract Selection

In the first step, one author (Rater #1) screened the articles by title, abstract, and keywords using the following assessment scale: 0 = the article does not debate consciousness itself (the term consciousness appears in the title or the abstract but with general meaning (e.g., people are conscious of their life, etc.); 1 = the rater doubts this article; 2 = the article debates some aspects related to consciousness. In total, 20% (4100 studies) of the total number of articles were selected with random data extraction (using SPSS software; SPSS Inc., Chicago, IL, USA) for an independent rating made by Rater #2. The agreement between the two raters (Cohen K analysis) was predetermined; it had to be over 0.90 to proceed with the second step of the screening process. If the agreement value was below 0.90, a third rater was involved in the screening of abstracts, and then a second analysis was performed. The third author (Rater #3), who had senior experience in systematic reviews and consciousness issues, checked the quality of data collection, helping to solve the discrepancy between the evaluations of the first- and second-rater.

#### 2.3.2. Step 2: Full-Text Selection

In the second step, two raters (#2 and #3) analyzed all full-text articles extracted during step 1 to assess them for final eligibility. The assessment scale used in this step was as follows: 0 = the article does not report theoretical aspects related to consciousness; 1 = the rater doubts this article; 2 = the article discusses theoretical aspects related to consciousness. The term “theoretical aspects” was used in referring to papers that accomplished three things: (a) describe a theory of consciousness; (b) describe one dimension of the DM (see below for its description) concerning consciousness as a topic; (c) describe a specific issue concerning different models of consciousness. In the case of a disagreement between raters regarding a selected study, consensus was achieved by means of discussion involving Rater #4, who read the article/s and participated in the discussion.

#### 2.3.3. Step 3: Data Extraction and Management

In the final step, full-text analysis of the selected records classified as “included” in the previous step was performed. Raters #2, #4, and #5 independently analyzed the selected articles. Each of them completed the DM (see below for the full description) and then summarized each theory of consciousness. Additional relevant papers were retrieved through a cross-reference check. The bibliography of each article was read to collect information on other studies not included in the final list.

### 2.4. Outcome Measures

Before beginning the review process, we developed a DM. The DM is a thematic grid used to quantify how each article, belonging to one specific theory, debates/analyzes specific issues. Dimensions are selected according to (i) important questions that emerged from previous consciousness studies [11,12,13,14,15] and (ii) questions on which authors wanted to point out.

### 2.5. Main Dimensions Analyzed

#### 2.5.1. Neural Correlates of Consciousness (NCCs)

NCCs are defined as the minimum neuronal mechanisms jointly sufficient for any one specific conscious percept [8,9,16]. In each article, the rater analyzed how much NCC was debated (quantifying this dimension using the Likert scale described below). Further, the rater reported the DM in the main brain structures. Moreover, the raters indicated whether the NCCs were described at the neuronal or brain area levels and whether the NCCs were correlated to a process involving different neural circuits. In this review, we did not consider the distinction between content-related consciousness NCC (i.e., the local state) and awakening state NCC (i.e., the global state or the activity which determines a particular phenomenal distinction within an experience) as eligibility criteria. Rather, raters reported more specific information when a clear description was found in the analyzed articles.

#### 2.5.2. Association between Consciousness and Other Cognitive Functions

In this dimension, raters were asked to analyze how much each article explained the relationships between consciousness and other cognitive functions such as attention, memory, mental imagery, and so on. In each study, raters were asked to analyze how much the theoretical process linked conscious information to cognitive functions (specifically about information processing). For instance, raters were required to analyze how stimuli conscious elaboration is related to memory or how consciousness generation mechanisms work in relation to attentional functions [17].

#### 2.5.3. Translation from Theory to Clinical Practice

In this area, we spotlighted information useful for the clinical management of patients with neurological/psychiatric disorders related to consciousness manifestations. Raters evaluated if each article provided information about clinical implications for either the diagnosis or the treatments of patients (e.g., with disorders of consciousness or with loss of consciousness due to epilepsy, etc.). Moreover, raters evaluated if the article reported any implications that would determine some prognostic markers based on the theory described. Specifically, the translation from theoretical aspects to clinical practice was examined, looking for words such as “marker”, “recovery of consciousness”, “diagnosis”, “clinical impact”, and “clinical implications”, as is reported in the supplementary materials.

#### 2.5.4. Quantitative Measures of Consciousness

Raters observed if an article reported information about the quantification of consciousness levels. We analyzed if instruments/tools to measure consciousness were described and if tailored algorithms were reported. Special attention was paid to the “index” definition directly related to the consciousness definition reported in the theory.

#### 2.5.5. Consciousness, Sensory Processes, and the Autonomic Nervous System

This area was chosen to explore if the selected theories of consciousness debate on the relationships between peripheral information derived from the autonomic nervous system (ANS) (also including sensory processes) and consciousness. In detail, raters were asked to quantify how much the elaboration of information coming from ANS was described in each article and how this information was linked to a consciousness percept (e.g., pathways that provide visceral sensation to conscious perception).

#### 2.5.6. Subjectivity

The final dimension analyzed by raters refers to how each article/theory of consciousness explains where subjectivity comes from and how it is related to conscious experience. A specific definition of subjectivity was not provided to raters to avoid the exclusion of new concepts and alternative viewpoints about subjectivity. In this sense, raters were instructed to search within for text-specific words such as “subjective experience”, “subjectivity”, “first-person experience”, and other terms linked to the discussion about how subjective experience could emerge from neural activity, and then to analyze how much this topic is analyzed in the text of each article.

### 2.6. Outcomes

#### 2.6.1. Quantitative Outcomes

For each study collected, we analyzed the full text, giving a score on a Likert scale ranging from 0 to 5. The score for each dimension was assigned according to the following criteria:

0 = the issue was not debated in the article.

1 = the article gave minimal attention to the issue. The theme was cited in the text (introduction or discussion) as a marginal topic that used fewer words.

2 = the issue was not reported in the abstract. The theme was debated in the text using some sentences as collateral/secondary issues in refers to other main topics.

3 = the issue was not reported in the abstract but there was a short paragraph that discussed the issue reporting specific references.

4 = the issue was cited in the abstract. In specific sections of the paper (introduction, results, or discussion) there was at least a long paragraph on the issue reporting specific references.

5 = the issue was cited in the title and/or in the abstract and/or as a keyword. The issue was the main topic of the article, which was debated in different parts of the text.

#### 2.6.2. Qualitative Outcomes

A series of information, although not evaluable in a quantitative way, was also collected from full texts.

1. Main definitions of consciousness. Raters were asked to report if there was a main definition of consciousness (e.g., consciousness is/represents/serves/refers to/consists of/results in/defined as/has to do with) linked to the main theory described in the articles they read. The definition of consciousness expressed by other authors and only cited in the text was not reported unless strictly related to the theory presented in the article. Definitions of what is not consciousness were also included.
2. Definitions of consciousness’ components/parts/sub-elements. Raters noted any definitions concerning parts and elements of consciousness and their definitions (if available).
  Related terms/features. Raters reported specific terms and/or features concerning the nature of consciousness as described by the single article/theory that is complementary to the main definition.

## 3. Results

### 3.1. Review Results

**Figure 1:**
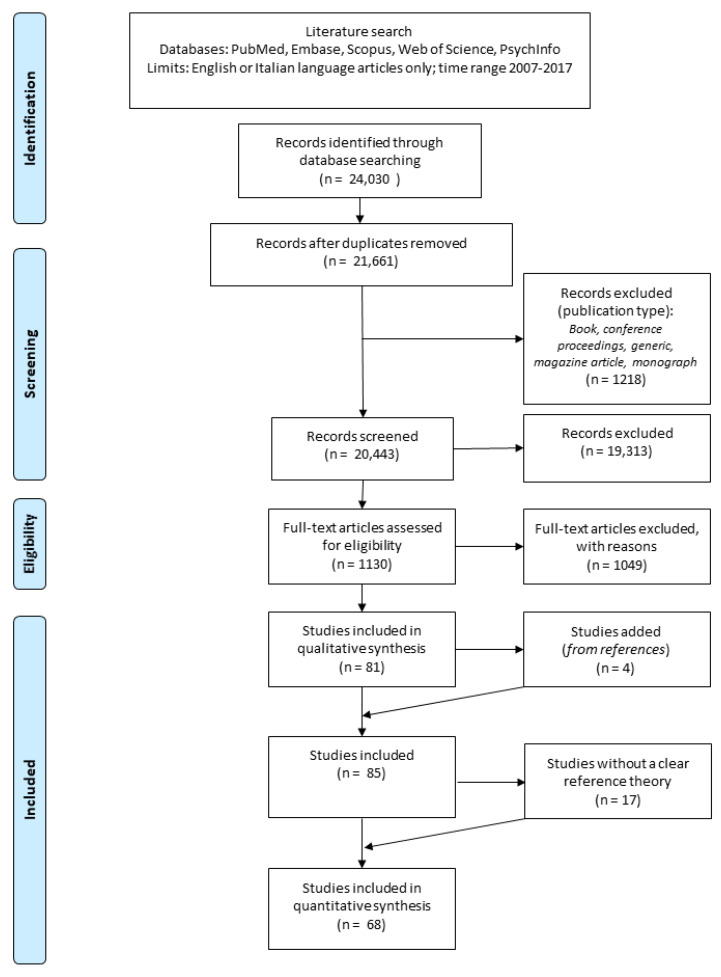
Prisma flow diagram.

The search identified 21,661 records after duplicates deletion performed in the step. In total, 11130 full-text articles were analyzed in step 2. Further, 68 articles were analyzed using the DM (see Figure 1).

The criteria used to exclude records before the final analysis consisted of using the term “consciousness” with a generic meaning (e.g., “conscious about their condition”) or with a moral viewpoint (e.g., “conscious about the consequences of his actions”).

**Figure 2:**
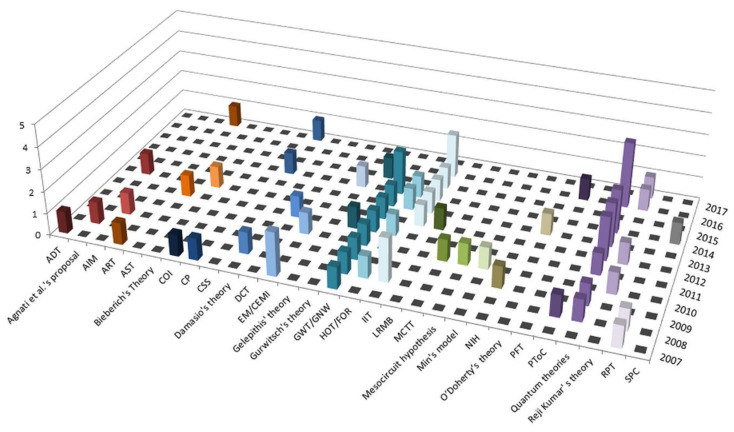
Graphic representation of the number of articles found for each theory in the period analyzed. ADT: apical dendrite theory; AIM: activation/information/mode-synthesis hypothesis; ART: adaptive resonance theory; AST: attention schema theory; COI: cross-order integration theory of consciousness; CP: centrencephalic proposal; CCS: consciousness state space model; DCT: dynamic core theory; EM/CEMI: electromagnetic field hypothesis/consciousness electromagnetic Information field theory; GWT: global workspace theory; GNW: global neural workspace; HOT/FOR: higher order theories of consciousness/first order representational theory; IIT: integrated information theory; LRMB: layered reference model of the brain; MCTT: memory consciousness and temporality theory; NIH: network inhibition hypothesis; PFT: passive frame theory; PToC: psychological theory of consciousness. Q theories—Orch OR: orchestrated objective reduction theory; single particle consciousness hypothesis; quantum no-go theorems; the three layer model; Koehler’s mathematical approach; timeless and spaceless; the four-dimensional Einstein; RPT: radical plasticity thesis; SPC: semantic pointer competition theory of consciousness; Agnati et al.; Bieberich’s; Damasio; Gelepithis’s; Gurwitsch’s; Min’s; O’Doherty’s; Rejikumar’s. The Mesocircuit hypothesis is not abbreviated.

During the analysis of the final 68 articles, we identified 29 different theories of consciousness (Figure 2). Each of them was synthetized following alphabetical order. Figure 2 graphically represents the number of record distribution over the years.

**Figure 3:**
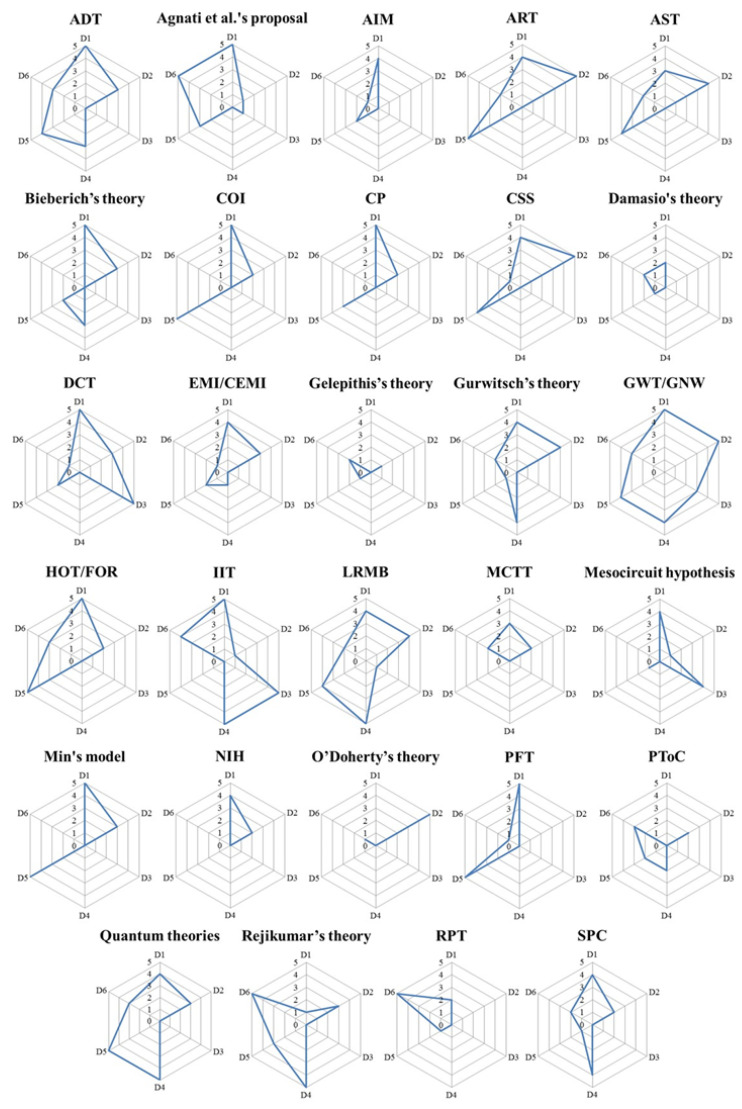
Graphic representation of the maximum scores obtained in the six dimensions analyzed by each theory. The figures depicted the scores achieved by each theory (displayed in alphabetical order) in each dimension of the dimensional model. We considered the maximum score for each theory among the articles belonging to it. The y-axis represents the scores ranging from 0 to 5. **D1** refers to the neural correlates of consciousness dimension. **D2** refers to the association between consciousness and other cognitive functions dimension. **D3** refers to translation from theory to clinical practice dimension. **D4** refers to the quantitative measures of consciousness dimension. **D5** refers to consciousness, sensory processes, and the autonomic nervous system dimension. **D6** refers to the subjectivity dimension.

Figure 3 reports the maximum score obtained by the articles analyzed for each theory. In in dimension number 1 (NCC), the maximum score was observed in 11 theories; in D2 (Association between consciousness and other cognitive functions) in three theories; in D3 (translation from theory to clinical practice) in two theories; in D4 (quantitative measures of consciousness) in four theories; in D5 (consciousness, sensory processes, and the autonomic nervous system) in six theories; and in D6 (subjectivity) in two theories. Regarding the total number of dimensions debated by each theory, we found that the range was comprised of a minimum of two dimensions debated in the selected articles (NIH and O’Doherty’s theory) to a maximum of all dimensions, as reported in Table S2. The theories with the highest number of analyzed articles were the group of the quantum theories of consciousness (*n* = 12), the IIT (*n* = 8), and the GWT/GNW (*n* = 9), whereas 18 theories were described by a single article.

For each analyzed article, we reported the definition of consciousness (Table 1). Regarding the various definitions found, we decided to report the exact sentences found in each article to avoid misinterpretation. Frequencies of the main terms used in the main definitions of consciousness by each theory were also reported in Table 2.

Moreover, other terms related to definitions or sub-categorizations of consciousness were also reported in Table 1. Considering only adjectives/nouns reported before the word consciousness, we found 21 different terms related to consciousness or consciousness sub-categorization (in alphabetical order): access consciousness; background consciousness; core consciousness; elevated consciousness; extended consciousness; foreground consciousness; general consciousness; knowing consciousness; marginal consciousness; meta-consciousness; micro-consciousness; perceptual consciousness; phenomenal consciousness; primary consciousness; reflective consciousness; secondary consciousness; self-consciousness; specific consciousness; subconsciousness; temporal consciousness; and unreflected consciousness.

Table 3 shows the main NCC reported in the various theories. The most cited neural structures were related to sub-cortical structures, although several theories reported cortical structures as well, defining in detail NCCs that could refer to cortico-thalamic systems.

### 3.2. Analytical Description of Each Theory

In the next sections, we report a brief description of each theory analyzed (in alphabetical order).

#### 3.2.1. The Apical Dendrite Theory (ADT)

The apical dendrite theory (ADT) of consciousness, proposed by LaBerge and Kasevich [18], focuses on input-processing, which seeks to understand how consciousness is generated by neurons and how information processing sustains itself over a prolonged time period without apparent outputs.

The theory affirmed that the pyramidal neurons within the thalamus-cortical circuit are important anatomical structures for consciousness due to their long apical dendrite, which produce a special kind of electrical activity.

Authors focused on structures called “minicolumns”, which are mini structures composed of major pyramidal neurons, stellate neurons, inhibitory neurons, and axons. The minicolumn is regarded as the functional unit of the cortex [83], based on the observation that neurons in each minicolumn share many receptive field properties. This theory analyzed two main circuits of the minicolumn: the shell and axis circuit.

The first one is a one-directional input–output circuit that connects one minicolumn with another, and the processing component consists of connections between layer 2 and 3 of neurons within the minicolumn, creating a sort of “horizontal” circuit. The axis circuit, instead, is considered as an “input-stayput” circuit due to its apparent role as a holder of neural activity over time. It constitutes the “vertical” circuit of the layer 5 cortical minicolumn, which extended to the thalamus in a reciprocally connected manner in a looping way.

The theory assumed that sustained activity in a primary sensory area provides the ongoing activities for a background consciousness. Elevated consciousness for selected aspects of background consciousness is assumed to arise when sustained activity of a primary sensory area is sent to higher sensory areas, where a selected part of the sensory scene is amplified by attentional activity controlled by the frontal lobes.

Within this attentional selection process, the control for the selective amplification/suppression activity is hypothesized to be provided by frontal cortex axions, which connect with the thalamic nuclei that serve those microcolumns. On the other side, the thalamic nuclei receive both this frontal information and data from primary sensory microcolumns, which remain relatively constant.

The shell circuits in early sensory areas that receive V1 inputs identify object appearances and locations. Later, sensory areas process these identifications into categories. In the frontal areas, categories are organized into propositions that may be based mainly on activity in the shell circuitry, which is spread across many minicolumns. On the other side, the global axis pathway begins with the initial cortical registration of the sensory inputs in the primary sensory minicolumns, and it extends into the frontal cortex (where presumably special supporting functions are performed and act as holding circuits that sustain activity for extended periods of time). When the intensity of the apical dendrite component of the thalamus-cortical holding circuit is sufficiently high, it produces the cognitive event, which may be labeled as “having an impression” of something. Two kinds of pathways are shown to sustain this activity: global ascending pathways that connect posterior cortical columns with anterior cortical columns and the global descending pathways that connect anterior cortical columns with posterior cortical columns.

Finally, the authors analyzed the properties of the electromagnetic activity of the apical dendrite to determine what properties of conscious impressions they may support. Interestingly, they affirmed that clusters of neighbor apical dendrites developed electric field energy in their core structures from the combined electric field components parallel to the dendritic axes. The development of magnetic field energy between parallel electric field dipoles is much less significant compared to electric field energy coupling between pairs of parallel apical dendrites with internal collinear dipoles moving at the conduction velocity. According to the present theory, the electromagnetic field intensity would underlie our immediate impressions of consciousness.

#### 3.2.2. Agnati et al.’s Proposal

Agnati et al. defined consciousness “as the global result of integrative processes taking place at different levels of miniaturization in plastic mosaics” [20].

The authors mainly referred to Cook and Sevush’s idea of a single-neuron theory of consciousness [19,84]. Accordingly, the synaptic connections within a network could represent the substrate of cognition, whilst the ion-flows during the action potential could represent the substrate of sentience. The authors claimed that both cognition and sensitivity could be present in the glial cells (other than neurons), which could represent in this way a prerequisite for consciousness and subjectivity. Furthermore, it is postulated the existence of the so-called functional modules representing microcircuits of neurons and astrocytes, which are well-organized in specific patterns to carry out a specific process. The functional modules do not have anatomical boundaries as they are determined by a functional structure. Furthermore, the functional modules are characterized by both vertical and horizontal organizations. The vertical organization includes 3 well-segregated levels, namely the molecular, local circuit, and cell network. Each level is then characterized by a horizontal organization; thus, the molecular level includes transmitters and receptors. The local circuit includes aggregates of synapses working as a unit. The cell network is composed of neurons and glia cells representing a network. A set of different functional modules could correspond to diverse sensory information that needs to be gathered in a high order fashion to give rise to conscious processes. The functional modules perform the first-level integrative functions that transform sensation into perception. The sensory information deriving from the outside world is spread to the cortex by the thalamus, which receives and redirects both sensory (from the outside world to the cortex) and cortical (from the cortex to other structures/nuclei) afferences. The resting-state network (encompassing medial prefrontal, parietal, and cingulate cortices) activity has been considered by the authors as a background condition to bind different information together. However, what is the phenomenon that is directly responsible for such a bind is still under debate. The authors considered the synchronization of the neural activity (detected through the gamma range recording) as a possible explanation [20]. A similar hypothesis was advanced by Cook who affirmed that the contents of both cognition and subjective feeling were grounded in temporal synchronization of synaptic activity and firing of action potentials, respectively [19].

As for the anatomical structures involved in the emergence of consciousness, the authors focused on the key role of the thalamus in receiving and redirecting both sensory and cortical input [20]. Furthermore, they considered the role of the brainstem system as necessary for the emergence of consciousness. The locus coeruleus has a role in the arousal maintenance, the raphe nuclei (by means of its projections) has a role in slow-wave sleep maintenance, and the ventral tegmental area is crucial because of its projections to the limbic and prefrontal cortex (two areas implicated in reward, attention, working memory, and emotions). Lastly, the claustrum is considered as a region of integration between perceptual, sensory, and motor information. Its role is similar to the thalamus, the main different being the integration mechanisms. The thalamus is assumed not to directly integrate the activity of all of its nuclei.

#### 3.2.3. REM Sleep–Dream Protoconsciousness Hypothesis (AIM)

Hobson [21] took into account the definition of primary and secondary consciousness, as postulated by Edelman [85], along with rapid eye movement (REM) sleep and dream models, to hypothesize the existence of waking consciousness, defined as “the awareness of the external world, our bodies and ourselves (including the awareness of our awareness) that humans experience when awake” [21].

He analyzed the difference between the awake experience and the sleep experience in relation to Edelman’s definitions [86] before considering the evolutionary and developmental aspects of REM sleep.

Waking consciousness is richer than dream consciousness, especially considering the measurable evaluation between task and brain activity, or between background and foreground processing. This contrasts with dream consciousness, which appears richer than waking consciousness in creating a scenario with different mental images. For the author, dreaming abounds in features of primary consciousness, especially perceptions and emotions, which are produced by the brain without external stimulation, whereas waking is fulfilled by secondary consciousness.

The hypothesis gives particular attention to the cortical-thalamic and limbic systems, which are active in waking and REM sleep for conscious experience. Moreover, this hypothesis considers dream consciousness and REM to start at the age of 5 years or as late as 8 years (there is an open debate on this point). This is when brain development has advanced sufficiently so that the narrative organization of subjective experience becomes possible. Considering that REM sleep appears before dreaming, the authors suggested that REM is one of the first signals that indicates that the brain is preparing itself for gradual development of integrative work among different functions, one of which is consciousness. The activation of the forebrain in the absence of external input during sleep could be important for the provision of an automatic, built-in, self-organized process that offers a spontaneous solution to the so-called binding problem, as demonstrated by the fact that REM sleep enhances sensorimotor integration.

The theory considered three factors that may be computerized: activation (A) (large parts of the brain are not only a collection of passive reflexive circuits but they also possess the means of regulating their own activation); input–output gating (I) and input–output gate control (factor I), which is mediated by a brainstem [87] that guarantees the coordination of factors A and I; modulation (M) (all modulatory brain cell populations in the brainstem during the move of being awake to nREM sleep to REM sleep, the so-called REM-off cells (which are active during waking and inactive during REM sleep [88], and REM-on cells (which are active during REM sleep and inactive during waking)). Joining the AIM model (which proposes that the wake–nREM–REM sleep cycle is the result of interactions between aminergic REM-off cells and cholinergic REM-on cells) to this ‘activation synthesis’ hypothesis of dreaming [89] (which posits that brain activation during REM sleep results in the synthesis of dream mentation), the authors affirmed that waking consciousness, with its impressive secondary features, might be present only in humans, who have the highly evolved and extensive cortical structures necessary to mediate the abstract aspects of conscious awareness.

#### 3.2.4. Adaptive Resonance Theory (ART)

Grossberg [23] aimed to develop a theory of consciousness that is able to answer to the so-called “hard problem” of consciousness. The authors stated that “consciousness is not just a whir of information-processing” [23], which purports to the already existing adaptive resonance theory (ART), where resonance consists of dynamical states represented by the amplification of neuronal firing and interactions between top-down and bottom-up processes [23]. This resonance is adaptive since it can trigger learning and memory traces. ART’s assumption is that “adaptive resonance refers to the fast learning that resonant states can drive” [22]. Resonant states bind together to build coherent world representations (i.e., learned bottom-up categories are bound with learned top-down expectations). The bottom-up categories and top-down expectations are bound only if there is a match between them. The matching between bottom-up external input and the top-down expectation results in resonant states. In this framework, all conscious states are resonant states, but the reverse is not true. The authors stated that the rules of ART align with several sensory and cognitive processes, as attested to by different evidence.

A key process for producing conscious states is represented by attention. The authors enlisted three different types of attention in the framework of the spatial cognition: (i) boundary attention (throughout propagation, the spatial attention selects a certain object for inspection); (ii) surface attention (acting in a scene with eye movements that allow the learning of view-invariant categories); (iii) prototype attention (the kind of attention produced via the ART model, which enhances critical features of certain bottom-up patterns). The latter kind of attention represents the attentional process involved in the ART mode of functioning.

Furthermore, the authors introduced the concept of the stability–plasticity dilemma to explain the way by which the brain can quickly learn new information without falling into catastrophic forgetting. This principle, in the author’s view, should be at the base of the way the brain unifies different sources of information into a coherent conscious experience. In order to explain the mechanism at the base of the stability–plasticity dilemma, the authors firstly considered the human beings as both intentional (meaning that the individuals learn expectations and make predictions about what is going to happen in the real world) and attentional (meaning that processing resources are focused on a restricted pool of information each time). If one has some expectations (i.e., searching for an object of a specific color among objects of different colors), then the attentional mechanism leads to the matching process between the bottom-up pattern and the expectations, amplifying the activation of the pattern resulting in a resonant state. In such a framework, only resonant states lead to rapid new learning. Hence, only experiences that attracted our attention and which have been learned can be considered conscious experiences.

As for the neural underpinnings of the learning process, a pivotal role is played by the thalamo-cortical circuit. Indeed, the first-order thalamic nuclei relay the sensory information towards cortical areas whilst the second-order thalamic nuclei receive inputs from low-order cortical areas. The learning of neural representations should correspond to the match between the first- and the second-order thalamic nuclei.

The authors explained the mechanisms underlying the ART model by using the visual system as an example. In this framework, the concept of attentional matching was introduced, meaning that matching between bottom-up and top-down processes were responsible for learning mechanisms. Specifically, whenever an individual receives an input, this can lead to the activation of target cells (bottom-up mechanism), while, at the same time, top-down mechanisms activate the on-center cells (inhibiting the off-surround of the same cells) and the attentional focus arises from the eventual match between top-down and bottom-up mechanisms. The attentional matching is characterized by a reinforcement mechanism as the attentional focus boosts the bottom-up process, which, in turn, emphasizes the top-down process. Thus, the learning process occurs only if there is a good match between bottom-up and top-down processes. Conversely, when something new has to be learned, it is necessary to activate the orienting system. This situation happens with unfamiliar events that are related to a mismatch between bottom-up and top-down mechanisms. This “mismatched” situation elicits a memory search mechanism. Whilst the prefrontal cortex is supposed to be related to attentional matching, the hippocampus seems to be related to mismatch processing. Furthermore, evidence supports an association between attentional matching and faster gamma oscillations, while mismatch processing appears to be associated with slower beta oscillations.

Furthermore, the authors also considered the role of emotions because they are different from visual and auditory inputs since they are internally generated. However, even in this case, a cognitive-emotional resonance process has been hypothesized by using feedback interactions between invariant objects categories, value categories (representations), homeostatic representations, and object-value categories that receive object and value category inputs.

#### 3.2.5. Attention Schema Theory (AST)/Graziano’s Theory

The present hypothesis suggested that awareness is a perceptual reconstruction of the attentional state. The fundamental position of Graziano and Kastner [24] was that consciousness is not an emergent property but is itself information computed by an expert system.

According to previous results [90,91], the authors affirmed that “the machinery that computes information about other people’s awareness is the same machinery that computes information about our own awareness” [24]. In other words, when a subject constructs a perceptual model of someone else’s attention (e.g., “person A is aware of X”), he/she also computes secondary information such as “I am aware of X”. In this sense, social perception is the mechanism through which stimulus representation surpasses neural competition derived from other representations.

For this theory, awareness is a form of meta-social intelligence reconstructing someone else’s thoughts, beliefs, or emotions, which also determines the state of someone else’s attention. Consequently, when social perception is applied to oneself, it provides not only a description of one’s own inner thoughts and feelings but also “a description of one’s awareness of items in the outside environment” [24].

To support their hypothesis, the authors cited results from studies on neglect and out of body experience, discussing the role of the superior temporal sulcus, temporal-parietal junction, medial prefrontal cortex, and the frontal-parietal circuits as key areas for social perception. In relation to this point, the authors discussed interesting predictions derived from their theory. For instance, they hypothesized the existence of different kinds of neglect associated with two neural networks—one for controlling attention (neglect caused by damage to parietal-frontal attentional mechanisms) and the other for perceptual attention (caused by the temporo-parietal junction and superior temporal sulcus impairment).

#### 3.2.6. Bieberich’s Theory

In the model proposed by Bieberich [25], “consciousness determines what is perceived as reality” [25]. It is composed by sentyons that belong to the physical world.

In the authors’ view, the binding mechanism is at the base of the perception of reality, i.e., there are stimuli (visual, auditory etc.) within the space (i.e., environment) that are consciously perceived in an internal space (i.e., endospace); in doing so, the system needs to bind together different information deriving from the space to consciously experience the endospace. This binding mechanism should be operated at the single neuron level. However, according to the authors’ view, in the neural space there would only be a summation process without any integration mechanism leading to conscious perception.

The dendritic trees are sites where the input signal starts and are summed with other signals: The sum of different signals, when surpassing the threshold level, provokes the firing of a client neuron. The latter sends the signal to other neurons in the network, and, in turn, it receives recurrent signals from the network’s neurons, thus creating a so-called “psychic loop”. Furthermore, the dendritic trees in a neuron receive the synaptic activity of different frequencies. Since the signal flowing inside the dendritic trees is slower than the signals’ propagation within the network, the signal looping times of a closed and distant network would be the same; this leads to an inverse connectivity due to the reverse relationship between the signal delay times within the dendritic tree and the recurrent signal delay times of the psychic loop. Thus, in the recurrent network with inverse connectivity, the farther the signal travels outside of the client neuron, the shorter it travels inside the neuron when the signal returns. This recurrent network is supposed to have a fractal architecture. The idea at the base is that the maps of a single part (e.g., dendritic tree inside the client neuron) are similar to the whole map (e.g., the neural network). To switch from the whole map to part of the map, a downscaling mechanism should be applied. Both mapping in a fractal way and downscaling are mathematically defined by the author who attempts to explain how these principles could describe how the entire brain functions. By assuming a refractal structure and by applying a downscale computation, it would be possible to describe both macro- and the micro-neural structures. These mechanisms define the recurrent fractal networks intended to underlie the emergence of consciousness as defined by the author.

Within this view, consciousness (derived from the activity of the recurrent fractal networks) resides at a single neuron level. However, because the networks connected between them are structurally fractal, consciousness is also diffused, as hypothesized by the global workspace theory [92].

In discussing the brain areas associated with consciousness, the authors suggested that the olfactory system were the more likely candidate to be the site of consciousness due to neuron features. It could at least represent a system with the minimum requirement for consciousness. Another site linked to consciousness is represented by both the hippocampus and the emotional centers of the brain (such as the amygdala). At a molecular level, calcium has been identified as responsible for the emergence of consciousness. Furthermore, by applying the downscaling, the fractal structure is supposed to present at the molecular level (at the level of ions channels and calcium).

#### 3.2.7. The Cross-Order Integration (COI) Theory

The COI theory provides a hypothesis for the NCC, focusing on the following question: “Why would this particular neural feature, rather than another, correlate with consciousness?” [26].

One of the COI assumptions is that psychological events and states are representations in the brain, some of which can be conscious whilst others can remain non-conscious. Yet we can envisage both a conscious and non-conscious representation of the same stimulus.

Considering Rosenthal [93,94], Kriegel affirmed that “although representing something is not the mark of conscious states, it may well be that being represented is. This family of views is motivated by the basic idea that conscious states are states we are aware of” [26].

According to COI theory, conscious states arise from the integration, or unification (two representations become a single representation), of what is initially separate: (i) a first-order representation of an external stimulus and (ii) a higher-order representation of that first-order representation. Thus, when an individual has a higher-order representation that is unified with the first-order representation it represents, the conscious state consists of this representational unity. In this sense, the COI is different from the high order theories because high order representation is external to the conscious state, i.e., it is unconscious and does not take part in the subject’s overall phenomenology.

Therefore, to generate a testable hypothesis about the NCC using the COI theory, the authors identified three levels: (i) a floor-level representation that determines the specific contents of consciousness (e.g., visual and auditory inputs) once consciousness is present, rather than ensuring the presence of consciousness in the first place; (ii) a higher-order representation of that floor-level representation; and (iii) the functional integration of these two representations. There is a neural triad of correlates that need to be pinned down before we can specify the neural correlate of a given conscious state.

According to COI, the second and third elements of the triad—the functional integration with the higher-order representation—constitutes the correlate of consciousness and not the content of consciousness.

The second element requires the ability to direct the cognitive system onto itself (involving prefrontal cortex), which is associated with executive and other forms of cognition functions (e.g., state-metacognition) [26]. Finally, the theory describes the third element as “the process of unifying disparate bits of information into a single representation in a functionally significant way, that is, in such a way that the functional role of the single representation is in some sense more than the sum of the functional roles of the different bits of information making it up” [26].

The COI affirmed that there are seven possible hypotheses about the neural correlates of higher-order representations. Three hypotheses suggest a single neuroanatomical site (the anterior cingulate cortex (ACC), the dorsolateral prefrontal cortex (dlPFC), the medial prefrontal cortex (mPFC)), whereas three other hypotheses suggest two neuroanatomical sites (ACC and dlPFC; ACC and mPFC; and dlPFC and mPFC. Lastly, one hypothesis suggests three sites (ACC, dlPFC, and mPFC).

As for the NCC associated with the third element (integration), the authors cited some studies regarding patients who exhibit “misbinding” or “illusory conjunctions” [95] due to different activities not coordinated by specific brain areas. Specifically, the authors considered the results deriving from research on the visual system to hypothesize that the synchronized activities in the visual and prefrontal cortices would be responsible for a fusion of the visual world- and self-awareness. Consequently, these areas can be considered neural correlates of visual consciousness.

#### 3.2.8. The Centrencephalic Proposal (CP)

The so-called centrencephalic proposal, developed and revisited by Merker [27], is grounded in Penfield and Jasper’s assumption of a “highest integrative function which, while anatomically subcortical, is functionally supra-cortical” [27,96]. These authors claim that the brainstem structure plays a pivotal role in determining a certain level of consciousness. Indeed, lesions at the brainstem level can impair normal cortical functionality. Specifically, the main subcortical structures, which are part of this “brainstem system”, are the ventral tegmental area, substantia nigra, colliculus, thalamus, raphe nucleus, and pontine reticular formation. According to Merker’s view, consciousness can be defined as “the medium of any and all possible experience” [27] and, similarly to Penfield and Jasper’s assumption [96], it is determined by sub-cortical activity that influences the cortical one. Within this framework, Merker’s theory postulated the existence of the so-called “triangle selection”, which encompassed the (i) target selection, (ii) action selection, and (iii) motivational ranking. Each component is stand-alone as they can be defined in their own way; however, they are interrelated as the action selection could depend on the target, and the motivational ranking could influence both the target and the action selection. The authors claimed that to interact with the environment and to take any kind of decision, this interaction must occur in real-time and, usually, it happens in the form of simulation. Using Merker’s words, “The way this simulation is structured constitutes a conscious mode of function” [27]. Furthermore, when anyone is conscious, the above-mentioned interaction happens in an ego-centric frame of reference (where the contents of sensory consciousness are disclosed), which could be biased by the motivation. The neural structures involved in such interaction are represented by a system ranging from the colliculus to the periaqueductal gray. The colliculus, however, is considered the key structure in the present theory due to its bidirectional connections with the cortex and its laminar structure, which is strongly related to a multisensory topography. This multisensory layered structure (due to the afferences coming from different cortical areas) leads to a sort of multisensory integration mechanism. Furthermore, when the neural information crosses the collicular threshold, the cortical activity enters the mesodiencephalon, which is considered the first step for a process to be conscious.

#### 3.2.9. The Consciousness State Space Model (CSS)

CSS represents a three-dimensional neurophenomenological model composed of three dimensions: time, awareness, and emotion.

According to the CSS, consciousness is fragmented into two different but interrelated categories [28,29]. The first is core consciousness (CC), which is related to the here and now. For instance, this category supports a sense of agency, ownership, and first-person content (by using the authors’ words, “it encompasses the minimal self”) [28]. The second category is represented by extended consciousness (EC), which is related to both episodic and prospective memory, as well as to verbal thoughts (“it encompasses the narrative self”) [28]. These two categories are organized in an embedded way as the CC represents a ‘low-order’ category being strongly related to the body-sense, whilst the EC is far away from the body and is related to higher-order cognitive processes. Furthermore, each category is organized along a continuum of three dimensions. Specifically, the time dimension (strongly supported by the mnemonic functions) ranges from past to future (i.e., retrospective and prospective memory-related; EC) passing through the here and now (i.e., working memory-related; CC). Similarly, the awareness dimension extended from an absence of the awareness to a full-awareness state (included in the EC category), passing through a sensory and subliminal bodily awareness (included in the CC category). Moreover, the awareness dimension appears to be related to attentional processes that determine the degree of awareness itself. Finally, the emotional dimension is subdivided into two sub-components, namely the valence component (through which a positive or negative value is ascribed to a specific event; EC) and the arousal component (which represents the bodily and visceral activations in relation to a specific event; CC).

As for the neural underpinning of CSS, the authors attributed the activity of those regions belonging to the default mode network to the EC, whilst the activity of regions implicated in multisensory integration processes would be at the base of the CC. This implies a widespread set of regions underlying the CSS, which encompass both cortical and subcortical areas. Since CC and EC are assumed to be interrelated, the responsible network of such interaction could be represented by the frontoparietal network, which encompass both the executive control network (i.e., dorsal attention network) and the salience network.

#### 3.2.10. Damasio’s Theory

The aim of the study by Bosse et al. [30] was to test a simulation model related to Damasio’s theory of consciousness [97].

The authors considered three components as conceptualized by Damasio: (i) emotion as a neural representation (deriving from neural activation after a certain change in the environment); (ii) feeling as body change perception (the emotion causes body changes that represent observable emotional states); and (iii) core consciousness (or feeling a feeling) as the detection of the body change after a stimulus has occurred.

To create a simulation model of Damasio’s theory, the authors referred to the temporal trace language (TTL; [98]), which allows computing a change of the state if given temporal parameters. For instance, the change of state A into B is computed by considering the duration of state A, the delay from state A to state B, and the duration of state B.

By using this model, an emotion is the result of external stimulation (e.g., auditory), which is detected and induces an internal representation (which is supposed to be multidimensional). This, in turn, prepares the body to act (each representational dimension corresponds to a component of body preparation to action).

The feeling is related to two different dynamics in the model: the “body loop” and the “as if body loop”. The first represents real change in the body and the second represents a cognitive change related to the body instead of a real change in the body.

The core consciousness (i.e., feeling a feeling) occurs when changes caused by the external event/stimulus are detected and attributed to a specific event/stimulus. In this context, the temporal specification plays a pivotal role (see the TTL principles as specified before). Moreover, the authors stated that a false core consciousness could occur when, for instance, two events occur even if only one of these is detected. The body change may occur due to the event not being detected; however, the subject, in this case, would erroneously associate the occurred body change with the only detected event.

For neural correlate concerns, the authors referred to Damasio’s theory, which considers the pivotal role of the cingulate gyrus, thalamus, and superior colliculus [97].

In this framework, subjectivity is outlined as a possible explanation for different feelings related to the same event/stimulus in different individuals.

#### 3.2.11. The Thalamic Dynamic Core Theory (DCT)

In 2011, Ward proposed the thalamic DCT, which is grounded in similar mechanisms explained by other authors. Further, it consists of the synchronous firing of large cortical neuronal population [86,99]. However, different from previous theories, the authors affirmed that neither a specific cerebral structure nor a single specific kind of neural activity is necessary nor sufficient for explaining conscious states [31]. In other words, the theory assumes the existence of consciousness thanks to a distributed neural activity with a central role of the synchronized neural activity in cortico-thalamic circuits. The central role of the thalamus is supported by evidence on both patients with disorders of consciousness and from the functional data during the REM and NREM sleep patterns in healthy subjects. A novelty in this theory is represented by the distinction of two different neuronal populations within the thalamus, although this was already postulated by Jones [100]. Specifically, the “core neurons” are typical of sensory and motor thalamic nuclei and represent relay stations for sensory and motor pathways, whilst “matrix neurons” are typical of non-sensory thalamic nuclei and project mainly to frontal areas bound by the activity of the thalamus and cortex. Furthermore, the authors distinguished the burst mode of functioning as a promotion of sleep, and the tonic mode of functioning as a promotion of wakeful consciousness. In the latter condition, there is both thalamo-cortical and cortico-cortical synchronization, which are promote matrix neurons and are mediated by the thalamo reticular nucleus and brainstem. Within this framework, the cortex represents the site where computation takes place, thus creating the contents of consciousness. The thalamus integrates information derived from the cortical computation.

#### 3.2.12. The Electromagnetic Field Theories

The electromagnetic field theories consider neurons densely packed in the brain, with about 104 neurons/mm^2^. The basic idea of this group of theories is that when any neuron receives a signal from upstream neurons, synaptic transmitters stimulate ion pumps that cause the membrane to become negatively polarized. This massive membrane depolarization generates an electromagnetic field perturbation that influences the probability of firing of adjacent neurons, so the electromagnetic fields of adjacent neurons are not discrete but form a complex overlapping field composed of the superposition of the fields of millions of neurons [101].

The key feature of this superposition field is its ability to integrate vast quantities of information into a single physical system. Therefore, it can account for the binding of information for consciousness.

The consciousness electromagnetic information field theory (CEMI). CEMI [32]) is one of the theories that studied the above-mentioned process. Specifically, it affirmed that an electromagnetic field generated by brain activity inevitably influences the membrane’s dynamics and the action of ion pumps, thus creating a self-referred “feedback loop” that can represent the physical substrate of consciousness.

The authors suggested that, normally, this superposition field is not influenced by the external electromagnetic field at all, because the high conductivity of the cerebral fluid creates an effective ‘faraday cage’ that insulates the brain from most natural exogenous electric fields.

In contrast to other viewpoints, the CEMI’s proposal is that consciousness corresponds to the component of the brain’s electromagnetic field that impacts motor activity in the widest sense [101]. As McFadden wrote: “Digital information within neurons is pooled and integrated to form an electromagnetic information field. Consciousness is the component of the brain’s electromagnetic information field that is downloaded to motor neurons and is thereby capable of communicating its state to the outside world” [32]. This process can unify information and account for the unity of consciousness because a field is a continuum of information within space and time dimensions.

The authors criticized the need to use quantum mechanics to explain consciousness because the brain is not an optimal place for quantum coherence, considering the infinite number of information that should be stored in a qubit (unit of quantum information). In this sense, he suggested that our brain may be capable of implementing quantum algorithms to perform quantum computing without the need for physically unrealistic quantum states in the brain [102]. The CEMI accounts for all essential features of consciousness rather than for direct consciousness generation.

The electromagnetic field hypothesis (EM). The hypothesis of the electromagnetic field theory of consciousness by Susan Pockett [33] postulated that conscious experience is identical with certain spatiotemporal patterns in the electromagnetic field.

The theory was based on the idea that consciousness is a local, brain-generated, configuration of patterns, and the hypothesis was also confirmed by the evidence that localized electromagnetic fields are known to be capable of causing neurons to fire, which in principle offer a mechanism by which consciousness can cause a “behavior”. Consequently, for this theory, what is important is not individual neurons but the spatiotemporal pattern of neural firing over large groups of neurons, which generates a conscious field-configuration in some circumstances but not in others (explanation of why some processes could be unconscious).

The explanation offered by the present theory is that only some configurations of the electromagnetic field have the property of consciousness, although the definitions of these characteristics have not yet been investigated. The implications of the EM theory should be analyzed considering different perspectives. Specifically, the EM supports the existence of different types of consciousness: the human consciousness probably differs from the consciousness of bats [103] because the spatiotemporal electromagnetic field-configurations generated by a bat’s brains differs from those generated by a human brain. Furthermore, it would be theoretically possible to generate specific electromagnetic fields in the total absence of neurons that could be an experimental test of the theory.

The resonator. Lewis and MacGregor [34] proposed a model of consciousness based on the exchange of energy between the mental and the physical realms. Considering the limitation of natural science, they worked on nine hypotheses, two of which are particularly important [34]. The two most important hypotheses are:

(i) Hypothesis 7. The neural foundations and the nature, actions, and properties of consciousness are all describable in terms of a system of resonator elements by which consciousness participates in energy exchanges with the brain, which mediate both the generation of conscious awareness and the active modulation of volitional processes in the brain. All resonator elements produce P → M transformation and engage in collective partially-free M–M selections, but some (perceptual) may produce lesser or no M → P transformation”;
(ii) Hypothesis 8. (a) The capacity for consciousness is undergirded by the physical matter of resonator elements, which are target structures of a consciousness gene. (b) Consciousness itself is a dispositional form of energy (DE) that may relate to physical forms of energy, similar to how the phase state of a gas relates in a material way to its liquid phase. Conscious awareness and its selective release are parts of DE and under the partially-free regulation of home resonator DE advised by collective systemic DE. (c) The functional identity of conscious perceptions and feelings is intrinsically determined by the anatomical locations of neurons which either house resonators or are connected directly to non-neural cells which house resonators (the labeled line hypothesis).

Following these hypotheses, the authors introduced the concept of an energy-based resonator, intended as a brain element, that “may receive and project metabolic, traditional neural, and perhaps electromagnetic energy with traditional neural and brain elements”. The consequence of the introduction of the resonator is that the receiving and projecting connections of resonators with neural structures allows for the generation of a composite conscious–subconscious cooperative action [34].

The neural candidates for energy transformation have different locations according to resonator elements, and the theory presumes a special role for the astroglial systems and their modulators of metabolite transmission (e.g., glutamate receptors) to pyramidal cells as the conscious modulators of attention, which is in line with hypothesis number 9 (i.e., consciousness consists of dispositional energy generated by metabolic biomolecules in resonator regions of astrocytes, which undergird attention in the human brain). By merging the electromagnetic models with an astroglial effector, the total resonator’s influence on pyramidal neurons and their apical dendrites can be coupled with modulation of traditional metabolic and neural actions, as well as possible EM effects. This is the core of the theory that also offers an upgrading of consciousness levels citing the Damasio’s model [104].

#### 3.2.13. Gelepithis’s Theory

The theory described by Gelepithis analyzes different approaches of consciousness study, highlighting the problems related to all of them [105], especially for the definition of consciousness derived by these theories. Then, Gelepithis formulated a theory based on seven definitions that compose a definitional system [35]:

**Definition** **1.** *For a human, H, I call neural formation, N, a structure of interacting sub-cellular components across nerve cells able to influence the survival or reproduction of H.*

**Definition** **2.** *For a human, H, a neural formation is meaningful (symbol Nm), if and only if it is an N that influences the attention of that H.*

**Definition** **3.** *The meaning of a novel Sc (i.e., stimulus within its context), for the human H at time t, is whatever Nm is created by the interaction of Sc and H at time t.*

**Definition** **4.** *The meaning of a previously encountered Sc, for the human H, at time t is the prevailed Np of Ɲ.*

**Definition** **5.** *A human, H, is conscious of an external Sc, at time t, if and only if, there are Ɲm structures that correspond to Sc and these structures are activated by H’s attention at that time.*

**Definition** **6.** *A human, H, is conscious of an internal Sc, at time t, if and only if, the Ɲm structures identified with the internal Sc are activated by H’s attention at that time.*

**Definition** **7.** *A human, H, is reflectively conscious of an internal Sc, at time t, if and only if, the Ɲ structures identified with the internal Sc are activated by H’s attention at that time and they have already been modified by H’s thinking processes activated by primary consciousness at least once.*

#### 3.2.14. The Global Workspace Theory

The Global Workspace Theory (GWT). The core idea grounding the GWT is the presence of an interaction between bottom-up and top-down attentional modulation mechanisms which, throughout a broadcasting process, allows for a specific percept/event to became conscious [106]. This idea has been developed by several authors with slight differences. Baars et al. [38,40,44] stated that several inputs enter the so-called workspace and only the more salient ones are selected and broadcasted, thus becoming conscious. Since the selection of the more salient inputs can affect the already stored internal and external representations, this process has been considered particularly important for the interaction with the environment, thus influencing selection [40,44]. Similarly, Prakash et al. [39] affirmed that some information reaches a sort of buffer and, by means of the top-down attentional amplification, they became conscious. Successively, Raffone and Pantani [41] supported the existence of two different forms of top-down attentional modulation hypothesized in the GWT, namely attention for perception and attention for access. The first is supposed to affect both the phenomenal consciousness (i.e., related to the sensory information; phenomenally conscious states that are not cognitively accessible; [107]) and the access consciousness (i.e., content information broadcasted in the global workspace). Conversely, the attention for access is supposed to only affect access consciousness. Despite the segmentation of the top-down attentional modulation for access perception and attention, the layered model hypothesized by the authors is similar to models postulated in the above-mentioned works [38,39,40]. The authors claimed that the sensory maps generate sensory representations that pass through intermediate levels (i.e., the phenomenal registry and global workspace gate) before reaching the workspace where, thanks to the so-called consumer system, broadcasting process takes place, thus enabling a conscious experience.

As for the neural correlates of consciousness, it has been hypothesized that the key role of an extended cortico-thalamic system is the responsibility of different sensory areas [108] to bind and integrate sensory information. The prefrontal cortex has a pivotal role in the broadcast process and for access to consciousness [41]. Within this system, the thalamus has a key role in redirecting both sensory and cortical input [20]. Furthermore, the mediotemporal lobe plays a crucial role in organizing the subjective experience and episodic memory, following the theory presented by Baars et al. [44].

Besides the anatomical description of the neural correlates of consciousness, some authors supported the pivotal role of the neural firing patterns at the base of consciousness within the GWT. Specifically, Prakash et al. [39] suggested that the spatiotemporal patterns of the electromagnetic fields generated by neuronal firing can be indicative of the emergence of consciousness. Experimental evidence attested to more complex neural electromagnetic dynamics in conscious states/events compared to unconscious state/events. Similarly, Bartolomei et al. [46] considered the neural network synchronization as a key feature at the base of consciousness. It is determined by slow oscillations (theta, alpha, and beta bands) in a long-ray, as well as by fast gamma-band activity at the local level and supported by clinical evidence derived from epileptic patients with alterations of consciousness. Indeed, in this population, alterations of consciousness seem to be mainly caused by slow electrical activity in the associative cortices, which appear deactivated along with a subcortical activity during epileptic discharges. From this evidence, it is hypothesized that hyper synchronization (impairing both local and distant networks) underlies an alteration of consciousness. The neural structures mainly implicated in such hyper synchronization are represented by the corticothalamic system with cortical areas mainly involving the prefrontal and the parietal cortices [46].

Finally, among the retrieved works on the GWT, Sergent and Naccache [43] were the only ones to explicitly reference subjectivity. For the authors, this had great importance in determining what is consciously perceived. Indeed, the authors stated that if someone was conscious of something, he/she must be able to report (either verbally or not) the content of their consciousness. However, subjectivity has not been integrated within the GWT into the author’s article.

The Global Neuronal Workspace hypothesis (GNW). The GNW hypothesis [45] was born from Baars idea of a model where current conscious content is represented within a distinct mental space called a global workspace, which is a structure shared and updated by many specialized modules [45]. The GNW hypothesis proposes associative perceptual, motor, attention, and memory areas interconnect to create a unified space where information is shared in a global blackboard and is sent back to lower-level processors [42]. Dehaene and Changeaux proposed, in their simulations, that this neuronal workspace is anatomically formed by cortical pyramidal cells in layers II/III with long-range excitatory axons, particularly dense in prefrontal, cingulate, and parietal regions, together with relevant thalamocortical circuits [109].

For GNW, what we subjectively experience as consciousness is the selection, amplification, and global broadcasting of a single piece of information selected for its salience or relevance to current goals, while the rest of the neurons composing the GNW are inhibited. In other words, the hypothesis postulated that the availability of information is what we subjectively experience as a conscious state [110].

This global availability of information, which is the main core of conscious access, was hypothesized through two mechanisms. The first is the feed-forward propagation of information, during which input progresses through high-order areas in a feed-forward manner involving all probabilistic representations of the stimuli. All signals converge in high-order areas and then return to lower sensory representations that favor an increase in the power of representations compatible with current goals. The second mechanism postulates that the NMDA-mediated feedback connections (composed of GABAergic inhibitory interneurons) inhibit other neurons from amplifying the input that cross the threshold for the “ignition” and cause a self-amplification of the global state of the selected representation. According to Dehaene and Changeaux [109], “In GNW simulations, ignition manifests itself, at the cortical level, as a depolarization of layer II/III apical dendrites of pyramidal dendrites in a subset of activated GNW neurons defining the conscious contents, the rest being inhibited”. This is in order to prevent multiple ignitions.

The ignition mechanism has an important role in GNW. Some simulations demonstrated that it could fail to be triggered for very brief or low amplitude stimuli because it is not able to achieve sufficient reverberant activation during the feedforward process [111]. Moreover, several experiments with visual stimuli seem to provide robust results about the role of ignition. Indeed, some evidence seems to support that the early stages of non-conscious processing show a linear variation in activation, whereas conscious access is often characterized by a late non-linear amplification of activation through the cortex areas reported above [112,113,114]. The sharing of information creates a global workspace formed by high-level interconnected areas that work differently with respect to non-conscious stimuli, which are processed in parallel by specialized cortical processors. The global workspace allows for the explanation of when a novel operation is required, especially when a behavioral decision requires a subsequential serial strategy [115,116].

In conclusion, the GNW focuses on the well-delimited issue of how an external or internal piece of information goes beyond nonconscious processing and gains access to conscious processing, a transition characterized by the existence of a reportable subjective experience.

#### 3.2.15. Gurwitsch’s Theory

Gurwitsch accounted for the totality of conscious experience, delineating its structure and dynamics by using the phenomenological method. Using his words, Gurwitsch proposed a theory of the “articulation of the total field of consciousness and the patterns and forms in which co-present data are organized with respect to each other” [117]. According to the theory, any field of consciousness is organized according to the following three domains: theme, thematic field, and marginal consciousness. 

Gurwitsch defined the theme as “that which engrosses the mind of the experiencing subject, or as it is often expressed, which stands in the ‘focus of his attention” [117]. The thematic field has been defined as a domain of relevance. It comprises all data co-present with the theme and experienced as materially belonging and related to the theme. Finally, for the marginal consciousness, Gurwitsch claims that data in the margin are irrelevant to the theme. The only relation between marginal data and the theme is simultaneous occurrence. These are “data which, though co-present with, have no relevancy to, the theme” [117]. The author elaborated upon three distinct categories in marginal consciousness: (1) Self-awareness, which consists of awareness of one’s own inner thoughts, a stream of ideas, concepts, and inner speech running through the mind. Gurwitsch states that “self-awareness permanently and necessarily pervades all of our conscious life” [118]. The author referred to the self we are aware of in the sense of our “psychic self”. In addition to the sense of our psychic selves, we also have an ongoing sense of our bodies, corresponding to what Gurwitsch calls the corporeal or “somatic” self: “There is no moment in conscious life when we are completely unaware of our bodily posture, of the fact that we are walking, standing, sitting, lying down, etc.” [118]. Finally, insofar as we are aware of our body, we are aware of it as positioned at some location in the physical world, according to Gurwitsch: “Our body appears in experience under the perspective of the perceptual world and derives its positional index... from this horizon” [118]. Thus, we always have at least some marginal consciousness of where we are in the perceptual world: “The perceptual world has...the privilege of omnipresence” [118]. According to Gurwitsch, we pass from the theme to an item in the thematic field along a “line of relevancy”. Regarding relevance, the author wrote: “Two co-present items in the field of consciousness are relevant if they are felt to be ‘intrinsically related’ due to their ‘material contents’ transition”. In contradistinction to marginal data, items belonging to the thematic field not only present themselves along with the theme but are also experienced as intrinsically related to the theme due to the material content involved.

Yoshimi and Vinson, in 2015, proposed an extension of this theory, focusing on three points [37]:

1. Considering some results about inattentional blindness and changes in blindness, subjects sometimes fail to report seeing anything, so they hypothesized that there also exists a peripheral experience.
2. They distinguished several types of relevance, showing how one of these concepts (i.e., “causal relevance”) can be empirically tested. They introduced a new type of relevance called “predictive relevance”.
3. They developed the idea that the theme has a “variable size”, expanding and contracting, and sometimes disappearing.

Furthermore, Yoshimi [36] showed phenomenological and connectionism approaches as having the same mathematical solution for some problems, e.g., giving an interpretation of the space of possible brain state and the relationship with a possible conscious state using connectionist dynamical system approach. He described some axioms in his approach, such as the idea that the base state of a system at a time is sufficient for determining the main state of that system at that time.

#### 3.2.16. The Representational Theories: High-Order (HOT) and First-Order (FOR) Models

The representational theories affirmed that consciousness is directly linked to “mental representations” rather than to a physical state. The main technical question related to this group of theories could be expressed as followed: What makes a mental state a conscious mental state?

The representational theories were mainly divided into two groups: FOR and HOR representationalism.

There are several varieties of HOR yet they all share common neural correlates at the level of the prefrontal (PFC) and parietal cortex, which assumed a crucial role for the creation of high representation that link different information. Conversely, the FOR performs a distinction between post-sensory structures that correspond to the same neural correlates of consciousness in the HOR, which determine the so-called “general consciousness”. Further, sensory structures correspond to specific neural sensory areas and sensory thalamic nuclei, deputing to the so-called “specific consciousness”, which corresponds to the contents of consciousness [50]. HORs, based on the thinking of Locke and Kant [119,120], contrast with FOR, which affirmed that perceptual representations formed in the sensory regions and are able to determine a conscious mental state, as reported by Dretske [121] and Michael Tye [122,123]. In other words, the HORs affirmed that consciousness depends on the supra-mental representation, while the FOR argued that the supra-mental representation must be determined by a specific content so that the sensory structures have a pivotal role in determining specific consciousness [50].

The high-order thoughts (HOT) theory, one of the most cited HOR theories, posits that consciousness awareness depends mainly on mental representation in the terms of mental states that represent oneself as being in a representational character of a first-order representation [48]. In other words, as Rosenthal wrote, “What makes a mental state M conscious is that it is the object of some kind of higher-order mental state directed at M” [124]. To empirically sustain this position, Lau and Rosenthal [48] considered the results from different studies, showing how transcranial magnetic stimulation (TMS) applied to PFC can affect the awareness in healthy subjects without impairing their task performance [125].

At the moment, the debate about HOR and FOR is sizable, although it is important to observe the critical consequence derived from HOR, including the fact that “if PFC activity is necessary for all conscious experience, and if there is little or no PFC activity in infants and most animals, then either infants and most animals do not have conscious experience”, as was noted by Gennaro [126].

#### 3.2.17. The Integrated Information Theory (IIT)

The IIT tries to explain the enigmas of conscious experience. Within this framework, Tononi [52] offered a mathematical definition to measure whether a system is conscious, to what degree it is conscious, and to explain consciousness itself. The IIT claims that consciousness is determined by its causal properties and it is, therefore, an intrinsic, fundamental property of any physical system. Consciousness is linked to integrated information (identified by the symbol φ), which represents the information generated by a system that goes beyond what can be explained by its parts working independently [51]. The IIT identified the main properties of consciousness, namely information and integration. Information is defined as the reduction of uncertainty; indeed, it is the ability to discriminate among many alternatives. Thus, the more numerous alternatives to be excluded, the greater the reduction of uncertainty, and thus the greater the information [52]. Integration allows the unity of experience, which is due to causal interaction between the elements of a system. Thus, if they are disconnected, their performance breaks down [52].

More precisely, Tononi explains both the quantity and the quality of consciousness. The first corresponds to the amount of integrated information generated by a system and it corresponds to its irreducibility, φmax (i.e., the highest value of φ). The second is identified by the set of informational relationships generated within that complex system [52,54]. The quality of consciousness could be geometrically represented within a space, called qualia space, in which it is possible to see the complexity of informational relationship that determines it [52]. Citing Tononi: “Qualia space (Q) is a space where each axis represents a possible state of the complex, each point is a probability distribution of its states, and arrows between points represent the informational relationships among its elements generated by causal mechanisms (connections)” [52].

The cortico-thalamic system is one of the most important NCCs for IIT since cortical damage leads to the permanent loss of consciousness. On the contrary, the cerebellum removal, even if it is full of neurons, does not considerably hurt consciousness [52]. The IIT addresses the hard problem of consciousness by proposing a set of phenomenological axioms, ontological postulates, and identities. The axioms are as follows [15,55]:

- Existence: Consciousness exists—it is an undeniable aspect of reality. Paraphrasing Descartes: “I think, therefore, I am”.
- Composition: Consciousness is compositional (structured)—each experience consists of multiple aspects in various combinations. Within the same experience, one can see, for example, left and right, red and blue, a triangle and a square, a red triangle on the left, a blue square on the right, and so on.
- Information: consciousness is informative—each experience differs from other possible experiences. Thus, an experience of pure darkness is what it is by particularly differing from an immense number of other possible experiences. A small subset of these possible experiences includes, for example, all the frames of all possible movies.
- Integration: consciousness is integrated—each experience is (strongly) irreducible to non-interdependent components. Thus, experiencing the Italian word “SONO” (i.e., I am) written in the middle of a blank page is irreducible to an experience of the Italian word “SO” (i.e., I know) at the right border of a half-page, plus an experience of the Italian word “NO” (i.e., no) on the left border of another half page—the experience is whole. Similarly, seeing a red triangle is irreducible to seeing a triangle but no red color, plus a red patch but no triangle.
- Exclusion: consciousness is exclusive—each experience excludes all others. At any given time there is only one experience having its full content, rather than a superposition of multiple partial experiences; each experience has definite borders—certain things can be experienced while others cannot; each experience has a particular spatial and temporal grain—it flows at a particular speed and has a certain resolution such that some distinctions are possible, whereas finer or coarser distinctions are not.

The postulates are sufficient conditions concerning the requirement that each system has to satisfy to account for experience (reported from [55]):

- Existence: mechanisms in a state exist. A system is a set of mechanisms.
- Composition: elementary mechanisms can be combined into higher-order ones.
- Information: a mechanism can contribute to consciousness only if it specifies “differences that make a difference” within a system. That is, a mechanism in a state generates information only if it constrains the states of a system that can be its possible cause and effect repertoire. The more selective the possible causes and effects, the higher the cause–effect information specified by the mechanism.
- Integration: a mechanism can contribute to consciousness only if it specifies a cause–effect repertoire (information) that is irreducible to independent components. Integration/irreducibility φ is assessed by partitioning the mechanism and measuring what difference this makes to its cause–effect repertoire.
- Exclusion: a mechanism can contribute to consciousness at most one cause–effect repertoire, the one having the maximum value of integration/irreducibility φMax. This is its maximally irreducible cause–effect repertoire (MICE, or quale sensu stricto (in the narrow sense of the word)). If MICE exist, the mechanism constitutes a concept.

Finally, identities are posited between phenomenological properties and informational/causal aspects of systems. IIT identifies the central identity as follows: “An experience is a maximally integrated conceptual information structure” [53]. 

The maximally irreducible conceptual structure (MICS) generated by a complex of elements is identical to its experience. The constellation of concepts of the MICS specifies the quality of the experience (its quale “sensu lato”; i.e., in the broad sense of the term). Its irreducibility ΦMax specifies its quantity. The maximally irreducible cause–effect repertoire (MICE) of each concept within a MICS specifies what the concept is about (how it contributes to the quality of the experience, i.e., its quale sensu stricto, in the narrow sense of the term), while its value of irreducibility (φMax) specifies how much the concept is present in the experience. The experience is thus an intrinsic property of a complex of mechanisms in a state. In other words, the maximally irreducible conceptual structure specified by a complex exists intrinsically (from its own intrinsic perspective), without the need for an external observer.

#### 3.2.18. Layered Reference Model of the Brain (LRMB)

Wang et al. developed the LRMB theory [127], which affirms the hierarchical life functions of the brain as being divided into two categories. The first one is the subconscious life functions encompassing the layers of sensation, action, memory, and perception. These functions are provided by layers 1 to 4, which are inherited, fixed, and relatively matured when a person is born. The conscious functions include the layers of metacognition, inference, and cognitive functions sustained by layer 5–7. These functions are acquired, highly plastic, and intentionally controlled based on willingness, goals, and motivations [58]. In the model, the thalamus and cerebellum are considered key structures for consciousness. The role of the cerebellum in the cognitive and logical approach to consciousness was analyzed by the author who declared that it has a functional role of conscious status memory (CSM), which is a “new type of memory identified in the brain, it is supplementary to other types of memories such as the short-term, long-term, sensory buffer, and action-buffer memory” [127,128,129,130]. The thalamus, instead, was considered as a processor, empathizing its role of switching center through the number of connections to almost all parts of the brain such as the cerebral cortex, eyes, and visual cortex [131,132,133,134].

Analyzing the mathematical, cognitive, behavioral, computational, and structural models of consciousness, Wang et al. described the cognitive process of consciousness using denotational mathematics. They affirmed that a conscious state is a function (fc) that maps a Cartesian product of entire events (E) and the current status of conscious status memory (CSM) into an updated state of the memory in CSM. Entire events in an individual’s brain can be classified as external stimuli (S) and∕or internal motivations (M), E =S u M. Thus, external and internal sub-functions of consciousness (fc-e and fc-i) can be expressed using individual or combinatorial expressions.

#### 3.2.19. The Memory Consciousness and Temporality Theory (MCTT)

Dalla Barba and Boissè [59] redefined the concept of consciousness in the framework of the MCTT. Specifically, they claimed that consciousness is always related to a certain object. In other words, when someone is conscious, he/she is always conscious of something. Furthermore, they distinguished between temporal and the knowing consciousness. The latter refers to specific features of an object; consequently, there are many knowing consciousnesses that refer to the same object. Temporal consciousness, instead, encompasses three other different sub-categories of consciousness. The first one is represented by past consciousness, which corresponds to the remembering of something. This is strictly related to the self; it is not a piece of generic semantic information related to the past. The second category is represented by present consciousness, which is different from other constructs, such as the “objective present” and the “psychological present”, as postulated by William James. Present consciousness encompasses all the present events, perceptions, and sensations related to the self. The third component is represented by future consciousness, which encompasses the future possibility of being (taking into consideration the past and the present). The three components altogether (i.e., the temporal consciousness) make it possible for the temporal existence for the subject. Furthermore, the temporal consciousness allows one to perceive the uniqueness of an object against its multiplicity, which is, in contrast, related to knowing consciousness (e.g., I perceive one specific pen as mine rather than a generic object belonging to the category “pen”). For what concerns the neural correlates of consciousness, the authors claimed a key role of the mediotemporal lobe and its related structures in determining the temporal consciousness. However, their hypothesis was developed to better frame the confabulatory behavior of patients suffering from brain damages.

#### 3.2.20. The Mesocircuit Hypothesis

In 2010, Schiff formally described the so-called “mesocircuit hypothesis”, which considers the thalamus (and globus pallidus) as the key structure for conscious states due to its widespread direct and indirect connections with both brainstem and frontal areas [60]. The author notes that in normal conditions medium spiny neurons (MSN) in the striatum inhibit the globus pallidus interna, thus allowing the thalamus to communicate with the frontal brain areas, which, in turn, project to the striatum. If the inhibitory mechanism of the striatum ceased, the globus pallidus interna inhibit the thalamus, thus shutting down the necessary connections and activity needed to sustain a conscious state. Consequently, the entire network would appear dysregulated, and, without thalamic input, the frontal areas would not be able to communicate and regulate the striatum activity anymore. This theory accounted for both evidence from patients with disorders of consciousness following thalamic damages (see for example [135]), from neuromodulation studies [136,137,138] and the mechanisms underlying the paradoxical effect of the Zolpidem in restoring the level of consciousness, thanks to its inhibitory action on the globus pallidus interna.

#### 3.2.21. The Min’s Model

In 2010, Min defined consciousness as “a mental state embodied through thalamic reticular nucleus (TRN)-modulated synchronization of thalamocortical networks” [61]. More specifically, it “consists of each mental unit, which is an individual thalamocortical looping mechanism, no matter what cognitive stages it involves” [61]. According to the present theory, the TRN could act as a mediator for the signals derived from the other thalamic nuclei to the cortex and vice versa, exercising in this way a sort of “control” over the thalamic activity in a feed-forward fashion. Specifically, the neural synchronization is considered as a prerequisite to neural signal association, and it seems to be at the base of conscious events. In physiological terms, the synchronization takes place thanks to the inhibitory GABAergic neurons, which allow for the detection of classic gamma range signal (>30 Hz) (in general, the gamma range signal indicates the presence of information processing within a network). Because the TRN is equipped with GABAergic neurons, it is plausible that it is responsible for the synchronization of the cortical and thalamic neural activities given the rise of information processing and, hence, consciousness. In other words, the author’s hypothesis considered that the emergence of consciousness due to the thalamocortical activation above a specific threshold level could determine the synchronization of neural activity. Thus, it is predicted that when the system does not reach the threshold level of activation, the sensory stimuli simply pass through the thalamus without becoming conscious. Furthermore, the contents of consciousness are supposed to be determined by the degree and the distribution of the cortical activity. Indeed, given the connections between the TRN and the cortex, the presence of an attentional top-down modulation operated by the prefrontal cortex can be postulated. Specifically, the attention could act as a signal amplifier whilst it eliminates unnecessary information. At the same time, the working memory retains the elements which are processed during the so-called “attended conscious awareness” [61]. Even if the attentional process is strictly linked to the prefrontal cortex’ activity, this theory assumes the pivotal role of TRN in triggering prefrontal cortex activity. However, the attentional process is considered optional to the so-called “conscious awareness” and insufficient for the emergence of consciousness. The theory takes into account a background condition to be conscious, which is represented by arousal. Arousal is indeed considered a necessary condition for consciousness and, even in this case, it is assumed that it serves pivotal role for TRN due to its connections with the brainstem [61]. Finally, given the TRN’ modular structure (i.e., different sensory-modality maps exist within the TRN), the emergence of a unitary consciousness awareness is explained via a characterization of shagginess across the boundaries of the receptive fields of the TRN neurons [61].

#### 3.2.22. The Network Inhibition Hypothesis (NIH)

The NIH aims to explain consciousness from the evidence reported in epilepsy studies [5]. The idea is that the so-called “conscious system” is analogous to other systems (e.g., motor, limbic, etc.) by involving different cortical and sub-cortical structures and that consciousness system must include neural areas that govern functions such as attention (i.e., a prerequisite for consciousness), awareness, and an alert state. In this sense, the medial thalamus, upper brain stem, hypothalamus, interhemispheric regions (including medial frontal cortex, cingulate cortex, and precuneus), lateral frontal, and temporal-parietal associative cortices seem to be important structures involved in the loss of consciousness due to the absence of seizure. Therefore, they could have a role in the appearance of consciousness [5]. The authors observed that the high-frequency neural activities in the temporal lobe during seizures reach the midline sub-cortical neurons and are correlated to an inhibition of frontal and parietal activities with a decrease in cerebral blood flow (CBF), as shown by studies that used single photon emission computed tomography (SPECT; [139,140]). The NIH hypothesized that loss of consciousness is a secondary effect due to the dysregulation of the networks described above and that focal limbic seizures propagate subcortical structures involved in the arousal systems. This could be correlated due to decreased activity in the fronto-parietal association cortices [141] caused by an increase in effective inhibition, or a decrease in effective excitation, within these networks [141].

The NIH affirmed that the upper brainstem interacts with the cerebral cortex to maintain wakefulness. However, during seizures, if they remain confined in the mesial temporal lobe, then a simple-partial seizure occurs without impairment of consciousness. In the case of seizure activity from the temporal lobe to midline subcortical structures, the propagation of the contralateral temporal lobe is possible. Finally, the NIH hypothesized that temporal lobe seizures inhibit the subcortical arousal systems leading to activity decrement in the bilateral fronto-parietal associative cortex, causing a deep sleep-like activity that is followed by the loss of consciousness.

#### 3.2.23. O’Doherty’s Theory

In O’Doherty’s view, “Consciousness represents the storage of past events for use in future situations and it is altered by external experience of the organism”, resulting “from the gradual evolutionary development of the human information processing function” [62].

The framework adopted by the author encompasses evolution, learning, and behavioral theories. Hence, consciousness can be conceptualized as the product of an interaction between an individual and the environment, rather than something located inside the individual himself/herself. The author’s attempt is to overtake both dualistic and monistic notions of consciousness. Furthermore, language is considered a precursor of consciousness in an evolutionary fashion.

In other words, consciousness could be the result of an evolutionary process involving both learning mechanisms and information processing, which must be conscious (different learning and information processing exist that take place without awareness). Furthermore, the language is supposed to be involved in coherently connecting the experiences with their outcomes, so that language allows the categorization of past events and the access to them. Within this view, memory is considered a prerequisite for language development, as it allows for information storage. Finally, qualia are considered as a key feature of consciousness since an individual cannot be conscious in the absence of an experience/stimulation and its storage. Furthermore, the experience of qualia can be modified through language (which builds links between different stored information) and the interaction of an individual with other individuals and the environment (as it happens, for instance, in sensory illusions).

#### 3.2.24. Passive-Frame Theory (PFT)

The PFT, proposed in 2015 by Morsella et al., represents a framework within which consciousness can be viewed as a phenomenon strictly related to voluntary actions. Indeed, the authors defined consciousness as “a phenomenon associated with perceptual-like processing and interfacing with the somatic nervous system” [63]. In other words, consciousness determines the skeletal muscle outputs to produce adaptive behavior when different motor plans are available and in conflict with each other. This conflict arises due to the awareness of different contents (i.e., what is experienced in a given moment), which requires specific actions to reach the goal. The sum of the conscious contents represents the conscious field, which is continuously updated (through the frame-check process) to select the most adaptive skeletomotor plan in that moment.

As for the neural correlates of consciousness, the authors considered different evidence, concluding that both the sensorium hypothesis [142,143] supporting the pivotal role of perceptual regions of the brain along with subcortical structures, and the hypothesis supporting the pivotal role of cortical circuits, are valid. To disentangle the role of cortical regions and perceptual regions, they suggest future research study simple neural systems such as the olfactory one.

The theory did not analyze the subjectivity dimension which corresponds to the first-person perspective in the authors’ view.

#### 3.2.25. A Psychological Theory of Consciousness (PToC)

Shannon proposed a theory from a psychological and phenomenological point of view [64]. He defines consciousness as a multifaceted phenomenon that includes different forms that be sorted into clusters. Originally, Shannon identified three types of consciousness (identifying consciousness as the region of the internal subjective experience), hierarchically ordered and interdependent among them. They form a well-integrated system, which is defined as a “tripartite system” [64].

The first type of consciousness is the Cons1, the “sensed being or sentience”. It concerns the primitive and elementary aspect of consciousness that distinguishes living from non-living organisms. It has no specific context or structure, it is pervasive, and it is present all our life [144,145]. The Cons2, called “mental awareness”, is typical of higher-order mammals and is related to subjective experience, as well as to the contents and forms of those experiences, such as mental images, ideations, flows of consciousness, and internal verbal monologues. The Cons3, called “meta-mentation”, is the mental ability to take its own productions as object for further reflection. This type of ability has different manifestation, such as meta-observation, reflection, monitoring, and control. The Cons2 and Cons3 are interrelated and someone can go up a level (from 1 to 2) without stepping down onto level 3 [64].

The system of consciousness encloses different regions and each one presents different levels: There is the self (self 1, 2, 3) concerning personal identity, the world (world 1, 2, 3), which is inherent in the relationship between knowledge and world and, finally, the temporality (temp 1, 2, 3) that concerns the temporal part of the experience.

Shannon [64] has identified other non-ordinary levels of consciousness, which are considered “mystical experiences”, namely Cons 4 and 5. Cons 4 involves internal subjective experiences that the person does not feel like the product of his/her own mind (i.e., hallucinations); Cons 5 is considered by Shannon as “the highest state of mind”, according to which the inner subjective experience leads the subject to experience things as coming from something supreme. It is a phenomenon very similar to nirvana and to the bliss that implicates the transcendence of the self and thus becomes ineffable [64].

This theory also provides a structural analysis of consciousness, through the identification of a series of parameters. The first parameter concerns the sense of perception, as well as sensory information recording and perception and how they are considered pertinent to reality; the parameter of the sense of meaningfulness concerns the meaning attributed to the perceptual experience, while the aesthetic sense parameter allows giving organization and composition to experienced things.

Moreover, Shannon [64] described five main features of consciousness: the quality of sentience; having experiences; making experiences the object of reflections and mentations; orientation and disposition towards others; the eventual capacity for transcendence.

The author specified that consciousness is a dynamic system subjected to different operations. The first processes are differentiation and crystallization (from which the different levels of consciousness specified above arise). The process of distancing allows separation from the sentient being between one’s inner world and the surrounding environment and, therefore, permits a distinction between subject and object. This process is more evident towards the highest level of consciousness, even if it tends to decrease in Cons 4 and 5.

For what concerns the internalization, Shannon [64] stated that consciousness creates a mental domain in which the cognizer may operate even when actual action in the real world is not feasible. Moreover, Shannon described the reflexivization as a dynamic mental operation through which the products of mentation, in turn, generate other mentations; externalization, instead, concerns the ability of the mind to create objects that are not part of reality, even if they are experienced as real, e.g., hallucinations. Finally, PToC reported the semantic operations, which are defined as “semantic composition or narrativization, the poesis, metaphoricity, and enhanced creativity encountered in the psychedelic state, the interplay between losing oneself and concentrated control achieved in masterly skilled performance, role playing, and the adoption of modified identity as in the metamorphoses” [64].

#### 3.2.26. Q-Theories

In this section, we summarize the different theories based on hypotheses that implied quantum mechanisms to explain the appearance of consciousness.

The four-dimensional Einstein. Sieb [74] affirmed that conscious experience oriented in space and time is fundamental for a coherent conscious experience. Considering that there are three dimensions of space (length, width, height; when one person analyzes the experience of a room where one is reading this paper, there are many events like light, walls, windows, etc. separated in time) and one dimension of time (e.g., moving the mouse of a PC we observe a series of events separated in time and having cause–effect and past–future relationships), conscious experience can be said to have four dimensions.

Taking into account that space–time is any mathematical model that combines space and time into a single continuum [146], the author considered the notion of space cells [147,148] and time cells of the hippocampus, following the MacDonald idea according to where place cells and time cells reflect fundamental mechanisms by which hippocampal neural networks parse any spatiotemporal context into quantal units of where and when important events occur, thereby organizing elements in a conceptual manner [149].

The authors suggested that the posterior parietal cortex (PPC) and prefrontal cortex (PFC) may extract space-time interval inequality information from the hippocampus in a process of relational integration [74,150]. The authors considered that the sensory neurons involved in this representation of the information, whenever present in the environment, are the same that maintain the information in working memory. In this case, the role of PFC not only contributes to working memory but, instead, manages attentional focus to select information and allows for the executive functioning to control cognitive processes.

In this macro system, quantum mechanical description is close to the classical description, and the authors suggested that conscious experience is when quantum systems and classical systems agree. In other words, when we represent a quantum system in conscious experience, we represent the system as a large (macroscopic) system wherein it is possible to use classical physics to describe it [74]. For example, the authors reported that our visual system transforms a quantum mechanical feature into the classical physics of conscious experience, so that wave-particle duality is reduced to classical physics in conscious experience.

Koehler’s mathematical approach. Koehler’s work was devoted to analyzing the possibility of checking experimentally regarding whether the perceptual process can lead to the collapse of the wave function. The author demonstrated that it is possible to describe the quantum collapse using a new Clifford algebra, obtained by the assignment of a numerical value in A(Si) by our cognition, which determines new commutation rules [68].

The idea is that a mathematical proof of the collapse of the wave function could be given every time we observed a direct cognitive attribution of a numerical value to the basic element of the A(Si). In other words, the author affirmed that the quantum collapse is a transition from a superimposed linear dynamic to a new dynamic in which a semantic act (the direct involvement of information, relating mental entities whose consciousness is the basics representative) is involved. For example, the act of seeing starts when the image is formed on the retina, an image which is not considered as a simple photodetector but, according to this new approach, as part of the brain that allows the perceptual and cognitive processes to be directly involved in the analysis of information following the role of quantum mechanics [68].

A Holoinformational Model of Consciousness. Di Biase [151] proposed a quantum-informational holographic model of brain–consciousness–universe interactions based on the holonomic neural networks of Karl Pribram [152] (found in the holographic quantum theory developed by David Bohm [153]). Pribram developed a neural wave equation, demonstrating that the cerebral cortex is the site of a holographic information process called a multiplex neural hologram, which is dependent on local circuits of neurons without long fibers that do not transmit ordinary nervous impulses. “These neurons function in the undulatory mode and are above all responsible by the horizontal layer connections of the neural tissue where holographic interference patterns can be built” [154]. Therefore, the author’s model affirmed that the “quantum holographic brain dynamic patterns are conceived as an active part of the universal quantum-holographic informational field, and capable of generating an informational field interconnection that is simultaneously nonlocal (quantum-holistic) and local (Newtonian-mechanistic), i.e., holoinformational”. In this conception is the holoinformational non-local flux that permits the interaction of holonomic informational quantum brain dynamics—this theory considers the viewpoint of Eccles [155] extended dendrons with the quantum-holographic nature of the universe.

The orchestrated objective reduction (Orch OR) theory. The Orch OR theory [71] is grounded on quantum physics. Penrose noted that one of the fundamental questions in the study of consciousness is “how it is that any physical structure whatsoever can give rise to the puzzling phenomenon of awareness?” [156]. The theory, in contrast to strong artificial intelligence positions, which argue that all mental processes can be reduced to computational models, affirmed that consciousness depends on processes of the general nature of quantum computations that occur in the brain.

Using the argument of Gödel’s theorem [157], Penrose supports the view that consciousness is not a feature of computational activity, thus taking into consideration the mathematical possibility that non-computational activity could be the base for the appearance of consciousness [158].

Consequently, Stuart Hameroff and Sir Roger Penrose developed a new theory called “orchestrated objective reduction” (‘Orch OR’), in which the main actors are (i) the “objective reduction” (OR) and (ii) the brain microtubules as anatomical structures in which the quantum computation occurred.

The idea starts from the measurement problem derived from the two fundamental procedures in quantum physics. The first concerns the deterministic evolution of a quantum state linked to the Schrödinger equation [159] whilst the second is related to the collapse of the wave function, where probabilistic rules seem to be involved when a measure of a quantum state in a system is needed (measurement problem). Penrose supposed that an objective form of quantum state reduction (OR) could be a solution of this problem, hypothesizing that consciousness could be dependent by quantum computation present in the brain that terminated OR in some form.

As reported by the author, the “orchestrated objective reduction is a theory which proposes that consciousness consists of a sequence of discrete events, each being a moment of ‘objective reduction’ of a quantum state” (according to the Diósi–Penrose objective reduction scheme, where it is taken that these quantum states exist as parts of a quantum computations) [160]. This is primarily carried out on in neuronal structures. In Orch OR, consciousness is a collapsed, self-organized process on the edge between quantum and classical realms as reported by the authors [71].

The authors sought out structures in the brain that support such putative non-computational actions at the borderline between quantum and classical physics, which could plausibly have important influence on brain activity. In this sense, Hameroff suggested the role of microtubules (i.e., polymers with cylindrical forms of around 25 nanometers of diameters, a length of a few hundred nanometers, probably up to meters in long nerve axons) as one of the major components of the structural skeleton prevalent in neurons.

The theory affirmed that for OR to be operative in the brain, a super position of sufficient amounts of material accounting for gravitational self-energy is needed, undisturbed by environmental entanglement, where this reduction occurs following the above OR scheme in a specific time scale in line with conscious experience. Pragmatically, authors affirmed that a coherent quantum super position/computation evolves during the integration phases in integrate-and-fire brain neurons (the most logical place is supposed to be the post-synaptic dendrites and somata). This causes an increase in quantum super position until the threshold is met at time τ≈¯h/EG (where τ is the time until OR occurs and ¯h is the reduced Planck constant), when a conscious moment occurs. Each OR selects microtubules’ output states that govern axonal firings and regulate synapses. Synaptic inputs ‘orchestrate’ these quantum computations, from which they derived the name “orchestrated objective reduction” (Orch OR), a process that, in a progressive large scale, could be the base for consciousness.

The Quantum no go theorems and consciousness. Georgiev’s viewpoint is related to two problems intrinsically linked to theories of consciousness [6]. The first consists of the fact that the material world in classical deterministic physical theories is causally closed [161]. In other words, if brain states produce conscious experiences, then these experiences cannot possibly have an effect upon brain dynamics, which is already fully determined by the fundamental physical quantities of the brain such as mass, charge, length, and time.

The second problem is represented by the illusion that consciousness is able to make a choice between alternatives as a “free” system, since the deterministic frameworks make it an illusion produced by our subjective brain [162].

The author considered that consciousness should work in a real short time window, and that decoherence time for quantum states within cytosketal proteins was estimated to be around 0.1 picosecond [163]. This could be coherent with consciousness process, considering that minds operate on a millisecond timescale and that a theory of consciousness consider this issue.

Moreover, the author provided mathematical proofs on the first two no-go theorems [164,165], positing that they invalidate Frankfurt’s celebrated argument on the principle of alternate possibilities [166,167]. Georgiev’s main idea is related to the fact that “because there is no measurement of q-bit A that would allow one to distinguish unequivocally between any pair of non-orthogonal states, it follows that unambiguous determination of an individual quantum state jWAi of a q-bit A is impossible by only measuring the q-bit A” and subsequently that “an unknown quantum state jWAi of a q-bit A cannot be cloned to another q-bit B”. This implies that quantum theories of mind are the best candidates to explain consciousness, rather than the classical determinist theories of mind, as they avoid some paradoxes, such as the possibility to create perfect copies of one’s mind (and brain), the non-existence of self-identity, and Frankfurt’s thesis stating that free will exists in cases where the subject could not have done otherwise, guaranteeing the privacy of the mental state and our free will in relation to the presence of multiple choices [6].

The single particle consciousness hypothesis. Argonov [69] studied the unity of consciousness as one of the most important problems when approaching a consciousness theory. How spatiality and time unities to form stream of consciousness is a main topic. The author does not support the idea of macroscopic coherency as a collective quantum phenomenon working like a single super-particle [158], as well as the idea that microscopic intracellular collective quantum effects are more realistic than the macroscopic ones. Specifically, the author suggested that each electron has its consciousness, following the idea of Bohm and Hiley who said that “in some sense a rudimentary mind-like quality is present even at the level of particle physics. As we go to subtler levels this mind-like quality becomes stronger and more developed” [153].

The fundamental issues of this hypothesis are as follows: “Each electron (or fermions) is the subjective ‘observer’ of its quantum dynamics (energy, momentum, ‘shape’ of wave function). Each electron ‘feels’ its quantum dynamics as ‘own’ subjective sensations and volition” [69]. Most of the electrons (or fermions) in the universe have a primitive consciousness. However, some particles in biological cells (especially in the brain) have complex consciousness due to complex dynamics in complex organic environment. Animals are hierarchical structures of particles. Some chemically active electrons in animal brains are “on the top” of the hierarchy. Their dynamics are directly influenced by sensory data and have a direct influence over animal behavior. The author called them “pontifical particles (PPs)”.

These electrons work following synchronized dynamics in one brain and they “feel” the same performance and volitional act. The human brain might have many observers that share a similar “human” mind.

Argonov supposed that PPs or pontifical syncytium (i.e., networks of neuron connected, with common cytoplasm and many nuclei) [168] may be placed in voltage-gated ion channels (chemically active electrons in carboxyl groups of amino-acid molecules), which play a functional role in axonal signal transmission involved in different activities, such as thinking.

The three-layer model. The idea of this model is that there are different kinds of consciousness sustained by different neural correlates [73]. The proposed model considers communication among preconscious states, from maps to a single consciousness [169], postulating independent and distributed neural structures at three hierarchical levels: “The lowest Level 1, which may include subliminal and preconscious nodes. The nodes in the next higher Levels, 2 and 3, do not possess specific neuronal correlates and there is aggregation of several of the lowest consciousness and the unitary consciousness is defined at the highest Level 3.” Authors suggested that the “nature of the conscious experience would depend on what other nodes are accessible to the node at Level 3” [170]. The mind selects memories that emerge from any conscious state and are based on the stream of previous conscious information. Thus, states of consciousness depend on the degree to which preconscious and memory states are accessible to awareness [73]. The mediated variable of this hierarchical model are languages and metalanguages that work at Levels 1 and 2, whereas the highest node is not located in a physical space, but still allows for the unity of consciousness. Nonlocality, entanglement, and coherent behavior are required to model memories as quantum objects [171], which is an explanation for long range correlations inside the brain and in behavior. It has been proposed that the cognitive system, through its evolution, veils the nonlocal processes of the quantum interactions amongst consciousness states by creating classical narrative for them, suggesting that the entropy associated with the consciousness states is a projection of three-dimensional reality.

Timeless and spaceless. Li’s theory [70] focuses on a pre-space/time dimension starting from 4 principles. (1) “The quantum superposition principle is assumed to be universally correct; (2) any physical property, that is accessible by physical means, is extrinsic, and it is nothing more and nothing less than the relations of the entity to the rest of the world; (3) consciousness can feel or memorize the past largely determined the configuration of the emergent time; (4) equivalent principle is applicable to consciousness”.

The author introduced a super quantum state called Dao, which “contains everything but tells nothing” (similar to other theories of Bohm [172], Mensky [173] and Wheller-Dewitt [174]) but in which time and space are absent. Moreover, if the Dao is a composite number, then it is decomposed as an entangled state of a composite system, generating a smaller quantum state. When Dao is separated into two subsystems (consciousness and universe), time emerged, whereas space is defined in the further creation of subsystems from universe.

The four principles guided the mathematical demonstration of the theory, suggesting that the universe is completely determined and that the relative distance and mass (a negative mass for consciousness was hypothesized) could be defined by the entanglement entropies among physical entities separated from a pure quantum state.

The Conscious Agent Thesis. Hoffman and Prakash [72] focused their attention on the relationship between perception and reality, citing the interface theory of perception [175]. They presented several premises to introduce their model. The authors stated that (i) in evolutionary biology and psychological perspectives, the natural selection has shaped our perception to be an accurate depiction of reality especially of those aspects useful for our survival; (ii) veridical perceptions strategies tuned to the true structure of the world are routinely dominated by non-veridical strategies tuned to fitness. Therefore, strategy favored by selection is best thought as a windows interface of a PC instead of as a window on truth. Consequently, the color and shape of an icon for a text file do not entail that the text file itself has a color or shape, so our perceptions of space–time and objects do not entail that objective reality has the structure of space–time and objects [176,177,178]. Considering these perspectives, authors proposed a rigorous formalism called a conscious agent thesis.

According to the authors’ perspective, consciousness involves three processes: perception, decision, and action. As for perception, “A conscious agent interacts with the world (W) and, in consequence, has conscious experiences. In the process of decision, a conscious agent chooses what actions to take based on the conscious experiences it has. In the process of action, the conscious agent interacts with the world in light of the decision it has taken and affects the state of the world”.

They claim that every property of consciousness can be represented by some property of a conscious agent or system of interacting conscious agents. Then, the authors defined a conscious agent as a six tuble C = ((X,X), (G,G), P,D, A,N)), where: (X, X) and (G, G) are measurable spaces; P: W × X→[0, 1], D: X × G→[0, 1], A: G ×W→[0, 1] are mathematical formalism of Markoviank kernels, and N is an integer.

Regarding the explanation of the generation of a macro-subject in terms of assembling micro-subjects [179], i.e., combination problems, the theory of the conscious agent affirmed that it is necessary to discuss conditional probability of having a specific conscious experience, considering that most of our mental processes are unconscious processes [180]. Then, the theory of the conscious agent provides two ways to combine conscious agents, i.e., undirected combinations and directed combinations, giving a formal solution for the combination problem of conscious experiences explained through theorems. Finally, regarding the theorization of the object (the previous parts were described as a theory of the subject), the authors described the idea that “space–time and objects are among the symbols that conscious agents employ to represent the properties and interactions of conscious agents” [181]. Specifically, they observed that “the harmonic functions of the space–time chain that is associated with the dynamics of a system of conscious agents are identical to the wave function of a free particle”, identifying a formal system and how it works. This part characterizes the conscious agent thesis. The authors affirmed that “one particular object, the quantum free particle, has a wave function that is identical in form to the harmonic functions that characterize the asymptotic dynamics of conscious agents; particles are vibrations not of strings but of interacting conscious agents”. Consequently, this allows one to reinterpret physical properties such as position, momentum, and energy as properties of interacting conscious agents, rather than as pre-existing physical truths.

#### 3.2.27. Reji Kumar’s Theory

Reji Kumar suggested a model where consciousness is the result of the information processing taking place in the mind which, in turn, consists of information accepting, processing, and generating [76]. The information process was assumed to be similar among individuals (even if some variations can exist in internal processing); however, what subjectively changes is the information accepted. Furthermore, the author assumes that if the information’s entry into the mind is blocked, consciousness does not exist [76].

The present theory is grounded on four different axioms: (i) A model represents a piece of information; (ii) the mind compares and classifies different models; (iii) new models can be created starting from the existing models; (iv) the mind attributes a value/preference to each model.

The above-mentioned models consist of three different parameters. The α-models concern the sensory information deriving from the outside world and processed by the mind (e.g., an object in the external environment). They consist in pieces of sensory information that can be processed even without consciousness, and they are also called “pre-linguistic models” [76]. The β-models allow one to express one’s own experience of the external world (e.g., the name of the object in the external environment) and they consist of a one-to-one correspondence [76]. The γ-models are more complex and are useful to explain the experience of mind (e.g., a story or a poem; [76]). In other words, the α-models are supposed to contain raw data, whilst the β- and γ-models are deputed to raw data processing [78]. Furthermore, the models are characterized by a time component that is apparent in models happening in different instances (this means that simultaneous models have the same time component), and a space component which concerns difference in corresponding models [79].

In 2016, the author enlarged his theory by distinguishing the information from the knowledge. Whilst the information corresponds to data deriving from the outside world, the knowledge consists of forms that such information assumes [78]. Furthermore, the author attributed a pivotal role to memory by assuming the existence of temporary memory, which receives information from both the outside world and an individual’s inside world, as well as a permanent memory where information is stored after their processing and integration according to the α-, β-, and γ- models [78].

Moreover, in the first conceptualization of this theory, the author outlined subjectivity as a core component of consciousness [76]. The concept has been further developed by the author. The model could be associated with different realities, thus producing ambiguity and confusion [79]. In these cases, it is necessary to perform a choice by selecting some items among many others in an arbitrary way. This idea has been mathematically developed by the author as follows [79]. Given the individual’s models of consciousness (M ∈CX), the subjectivity is a function (f:CX →[−1, 1]) according to which the individual can like (f (M) > 0) or dislike a model (f (M) < 0). If the function value is equal to 0, the model must be considered objective. Furthermore, subjectivity changes over time and can be computed in different ways by considering the minimum/maximum of the values of the subjectivity of component models (fmin (M) = mini (f (Mi))), and by averaging the subjectivity of all component models (fave(M) = ∑i Mi/N, where N represents the number of sub-models of M). Similarly, the stability of subjectivity over time can be computed via the following formula (S = (x + b) − (x − b)), which can range from 0 (maximum stability) to 1 (maximum instability).

The central role of subjectivity in this theory has been supported by other authors who stated that subjectivity arises when preferences are associated with a specific model [77]; in other words, subjectivity acts when individuals choose among models. The authors assumed that, within the α-models, there is no subjectivity, as these models represent an information registry where stimuli processing is unconscious. When information is associated with an entity within β- models, the perception is subjective and therefore conscious. Similarly, in the γ-models, additional subjective attention is required to perform information-entity association. This is different than the β- models, which allow the association of single information with multiple entities [77].

#### 3.2.28. The Radical Plasticity Thesis (RPT)

In 2007, Cleeremans defined consciousness as “the brain’s theory about itself, gained through experience interacting with the world, and, crucially, with itself” [81].

The author proposed the radical plasticity thesis of consciousness by considering subjective experience as a core component. Experience is defined by the author as “something that takes place not in any physical entity but rather only in special physical entities, namely cognitive agents” [81]. Indeed, the author stated that whenever experience is experienced, something happens in the organism (such as experiencing emotions, retrieving memories, learning something new, and so on) and the organism itself registers its state. Metarepresentations play a pivotal role in this. The individual creates some representations based on the external input, and the metarepresentations inform the individual of his internal status by considering the previously created representations. Subjective experience occurs only if a certain system (i.e., individual) learns its representations. Consequently, consciousness is the awareness of both external and internal status.

Representations are always associated with the activation of neural networks, which can vary across different dimensions, such as the stability of time (i.e., how long a representation can be maintained active; prefrontal cortex involvement), strength (i.e., how many units are involved in a certain representation and how strong is their activation), and distinctiveness (i.e., how different a certain representation is from others; it is a measure inversely related to the degree of overlap among representations, and it characterizes the hippocampal representations compared to the cortical ones). Moreover, the representations can be different considering the extent to which they can influence behavior, the availability to control, and the availability to subjective experience. As such, three different forms of representation are described. The implicit representation is characterized by weakness so that they are out of the individual awareness and, as a consequence, out of control. However, this kind of representation can influence behavior. The second kind of representation is the explicit one characterized by enough strength to be available to individual awareness and thus to the control. They can influence behavior as long as the individual is involved in controlling them. The subjective experience is typical of this kind of representation. The third kind of representation is the automatic one characterized by high strength, which impedes these representations to be controlled. However, differently from the implicit representations, the individual is fully aware of this kind of representation. Within this framework, the availability of the representations to consciousness strongly depends on the representations’ quality; furthermore, the learning process can produce higher quality representations in time (this mechanism is at the base of the development of adaptive behavior). In keeping with this, the role of consciousness is to control those representations strongly enough to affect the behavior but not strongly enough to be considered automatic adaptive representations. From here it derives that (i) consciousness involves the subjective experience along with the control dimension; (ii) the availability to consciousness correlates with the quality of representations; (iii) the development of high-quality representations takes time; and (iv) consciousness allows flexible and adaptive control over behavior.

The metarepresentations have the same features as the representations (strength, stability, and distinctiveness). Furthermore, they have specific functions, allowing for individuals to communicate their status and to make predictions. Cleeremans et al. provided empirical evidence by means of simulation experiments attesting how a higher-order network can be trained to observe the internal states of another network and use this information to perform tasks required to know the internal structure of such internal states [80]. In this way, the core idea of the radical plasticity thesis is that “the brain continuously and unconsciously learns not only about the external world, but about its own representations of it. The result of this unconscious learning is conscious experience, in virtue of the fact that each representational state is now accompanied by (unconscious learned) metarepresentations that convey the mental attitude with which these first-order representations are held” [81].

#### 3.2.29. The Semantic Pointer Competition Theory of Consciousness (SPC)

The phenomenon of consciousness has been studied by the SPC through three different hypotheses. The first one supposes that consciousness is the result of a process within the brain that springs from neural mechanisms [82]. Thus, this hypothesis starts from the assumption that all conscious organisms possess a brain, and therefore complexes of neural processes represent a basic condition for consciousness to arise. Accordingly, consciousness is present in all entities capable of introspection, self-evaluation, and implementing behaviors that are indicative of consciousness itself.

The second hypothesis identifies representation by patterns of firing in neural populations, binding of these representations into semantic pointers, and competition among semantic pointers” [82]. The representations are created thanks to neuronal patterns of activation following the interaction within the environment. Consequently, representations of different natures (e.g., perceptive, motor, verbal) can be created. These kinds of representations are called “semantic pointers” [182,183,184], which, after a binding mechanism, can be combined into a single semantic indicator with symbolic function [185]. As for the neural correlates of consciousness, the present theory does not refer to specific brain areas, but rather considers the pivotal role of cellular systems activation in consciousness.

The third hypothesis assumes that consciousness derives from an interactive competition between semantic pointers after which the winning indicator determines the qualitative aspects of experience (i.e., qualia). The SPC addresses the subjectivity by assuming the existence of different qualitative experiences due to the linkage between different neural representations and semantic indicators. According to the authors, “Semantic pointers bind together neural representations of a situation, physiological changes, and cognitive appraisals to produce a combined representation” [82].

In addition to this qualitative experience, the SPC specifically addresses other issues concerning the main aspects of consciousness, namely onset and cessation, shifts in experience, different kinds of consciousness, unity and disunity of consciousness, and, finally, storage and retrieval. All these aspects are mathematically explained through computer simulations. Starting from the first aspect (i.e., the cessation of consciousness, such as in the case of sleep, anesthesia, stroke, etc.), it is linked to three mechanisms, which depend on the balance between neural excitation and inhibition. Neural inhibition involves a decrease in the number of semantic pointers. Conversely, in the case of epilepsy and convulsions in which neural hyperactivity occurs, the interruption of consciousness is due to the saturation of the semantic pointers network, which interferes with the mechanism of competition that no single semantic pointer can overcome, especially considering the threshold necessary to generate consciousness [82].

Regarding these kinds of consciousness, we must distinguish between a minimal form of consciousness linked to the “here and now” and a form of consciousness characterized by greater complexity that allows for self-representation [186]. The different kinds of consciousness depend on differences in complexity and the extent of the three mechanisms that generate experiences.

The unity of consciousness concerns the reasons why we experience in an integrated way experience and is made possible by the combination of the different mental representations and, therefore, by the association of the semantic pointers. On the contrary, this disunity occurs during the mechanism of competition, where there is no semantic pointer that prevails over the others other than different “winning” semantic pointers [82].

The SPC emphasizes the interactions between consciousness and memory since, during an experience of an event, similar experiences are recalled. This is determined by the storage and retrieval capacity of each semantic pointer [82].

## 4. Discussion

From the 68 analyzed articles, the present systematic scoping review found 29 theories of consciousness that show heterogeneous perspectives. The theories with the highest number of articles focused on them were quantum theories of consciousness (which represent a category including theories that refer to a quantum mechanism to explain consciousness), the IIT, and the GWT. The articles describing these theories were found in almost all of the considered years. This result was predictable considering the number of focus reviews published in the last five years on these theories (Hinterberger, 2015; Owen, 2019; Zhao et al., 2019). However, the total number of articles found is not directly correlated to the level of analysis of each theory on the themes we focused on. Indeed, different theories obtained the maximum scores in at least one dimension analyzed (in alphabetical order: ADT, Agnati et al.’s theory, ART, Bieberich’s theory, COI, CP, CSS, DCT, GWT, HOT/FOR, IIT, LRMB, Min’s model, Rejikumar’s theory, RPT) independently from the number of articles found (among the above-mentioned theories, ADT, Bieberich’s theory, COI, CP, DCT, LRMB, and Min’s model were represented by a single article).

### 4.1. Descriptive Analysis of the Results for Each Dimension

Subjectivity, NCC, and consciousness and cognitive functions were the most debated in the articles we found (i.e., dimensions with the lowest number of 0 scores during the assessment), showing the highest grade of variability.

The NCC dimension was the most studied among the others (the highest number of articles (*n* = 30; see supplementary materials for details) obtained a score of 4 or 5 in the NCC dimension during the assessment). Among the neural correlates of consciousness reported in Table 3, the thalamus, basal ganglia, and the hippocampus were the most reported sub-cortical structures, whereas the cingulate, prefrontal, and temporal areas were the most reported cortical ones. In general, the thalamo-cortical system was the macro system most described in the literature as the system able to sustain consciousness (intended as the neuronal mechanisms jointly sufficient for conscious percept), especially when consciousness is related to representation. However, our results highlighted the widest range of proposals. Consciousness was associated with different structures from a single electron to different brain areas. This is an important point that should be analyzed in the future, especially when studying the relationship between NCC and the “type” of consciousness they refer to. As recently pointed out by other authors [187], there could be a potential convergence among theories on the neural structures involved in the mechanisms that can explain consciousness and its alterations. Therefore, macro-perspectives are needed in the next future.

The dimensions called “consciousness and translation into clinical practice” and “subjectivity” were the two dimensions that obtained the lowest number of 4 and 5 scores during the assessment (3 and 6 articles respectively); in other words, they represent the dimensions with the fewest number of articles focused on them. In the first dimension, we analyzed the translation of theoretical models into procedures able to help patients with disorders of consciousness. The lack of findings in this dimension is likely due to the difficulty in translating the hypothesis made by the authors into clinical practice (i.e., definition of prognostic or diagnostic markers, ad hoc treatments, etc.). An example of these difficulties is represented by the challenges in developing technical systems able to help researchers in testing their hypothesis. Indeed, considering the hypothesis made by authors of the quantum theories of consciousness, it sounds difficult to test how a quantistic phenomenon can be observed/reproduced in a “wet” material such as the brain, or how we can experimentally modify the activity of specific cell populations (e.g., the glia), or to analyze the effect of vibrations present in some neural microstructures on human consciousness. However, this does not imply that this perspective should not be studied by a new generation of researchers able to match neurological and quantistic viewpoints. This reflects a real challenge for researchers and clinicians. Future research programs should be developed to foster new approaches that can help patients with disorders of consciousness through new therapeutic tools (e.g., neural stimulation programs) in parallel to new measures of consciousness (13 articles obtained a score of 4 or 5 in the Quantitative measures of consciousness dimension). This would allow for quantitative and qualitative evaluations of the outcomes.

Finally, we found 11 articles that obtained a score of 4 or 5 for the association between consciousness and other cognitive functions. As in the NCC dimension, we found interesting perspectives that ranged from the complete overlap of consciousness with other functions (e.g., memory and attention in the learning process) [22,23] to the definition of consciousness as a sort of metacognition in layered models (see [58]), which is debated in some articles that considered metacognition equivalent to consciousness (published after the time range of this review) [188,189].

### 4.2. A Definition of Consciousness: Problems and Perspectives

The number of articles that propose a definition of consciousness was quite high and almost all theories reported a main definition, offering several arguments and interpretation of the results that should be analyzed in the future. Indeed, the sentences analyzed, such as “consciousness is […]”, were supported by many interpretations—i.e., defining consciousness as “being aware of” something or as the “subject experience”. We also found definitions of “awareness” and “experience”, and, of course, some clear statements on what “awareness” and “experience” is not. However, we found that four terms— “(subjective) experience”, “information”, “processes”, and “(mental) state”—were used in most of the analyzed articles (Table 2) to define consciousness. These terms imply several reasons regarding their nature/structure and the ontology behind them. For example, “information” is a term that has been debated in the scientific literature over the last few decades [190,191]. New researchers can find many references sustaining a specific ontology linked to this term [192]. Although everyone can affirm to have an idea of what “experience” is, it is difficult to find a clear definition of what it is not. What experience is it? What is the structure of an experience? Is consciousness synonymous with experience or do we have to think about a tailored association between the structure of experience and the structure of consciousness? Some theories attempted to answer these questions, but the debate is still open and thus these questions still represent issues for the science of consciousness that researchers will face in the future.

We also found many new definitions and new subcategorizations of consciousness, such as “knowing consciousness” [59], “unreflected consciousness” [65], or “meta-mentation” [64], as reported in Table 1. These definitions constitute a new way to develop ad hoc perspectives and measurable concepts. Our impression is that authors are developing new models in which consciousness is not a monolithic meaning and can be sub-divided into different components (working hierarchically or in parallel), offering a test in experimental settings whenever possible.

### 4.3. Limitations

This scoping review does have limitations. Firstly, during the full-text analysis, we opted for a very strict selection of the articles to be included. Consequently, one of the articles’ criteria for selection was consciousness as the main object of an article. This rigid criterion implied that raters excluded records on topics related to other arguments such as body-representation, bodily-self, etc., or describing the elaboration of conscious vs. unconscious stimuli using fMRI or EEG paradigms (e.g., the existence of unconscious working memory, memory processes linked to subliminal stimuli only, etc.). Moreover, we excluded general theories that identified basic rules for life generation that explain consciousness indirectly, as well as papers that did not report clearly the original underlying theory.

We acknowledge that all of these arguments are linked to consciousness models and that our article selection process could have excluded works that contributed to the study of consciousness but, considering the difficulty of analyzing each collected model, we decided to limit our work to articles completely related to a consciousness model itself as the main topic, analyzing papers on these arguments only if they were linked specifically to a general theory of consciousness. The choice to include articles using strict inclusion criteria could have caused a loss of information, but we preferred to make this choice in order to offer a well-structured overview of the topic without diverging from the main aim of this scoping review. Another limitation was that we analyzed only the text of the articles included in the review found in the databases reported in the methodological section. As a matter of fact, details on some theories could be described in other papers published before the years considered in the present work or published in other online databases. Consequently, some missing information about either each dimension or definitions of consciousness may be due to the adopted methodology rather than to a real lack of information (this is the main reason why we inserted the Table 1 note “nf” (i.e., not found)). Moreover, the choice to search articles in “medical” literature databases could have caused the loss of important works marked by the conceptual (philosophical) perspectives on consciousness simply because they are not contained in those repositories. For this reason, we avoided distinguishing articles included as “conceptual” or “empirical” because many works contained both parts, as shown by the dimensional model analysis. Therefore, the limitation on the medical dataset and time range deployed in the search strategy determined that several articles that we knew to be important for a debate on consciousness models were not included in our analysis. There is an increasing amount of literature in computer science on the relationships between intelligence and consciousness (e.g., Drigas et al. presented a new layered model of consciousness that combines the dominant theories of intelligence adapted to recognized models of telecommunications and computer science [188,189]), affirming that cognitive processes contribute to the representation of knowledge. Some important metacognitive components are transformed into superior levels of consciousness (see [193]). Another example is provided by other studies that sought to define a precise formal definition of a conscious Turing machine, also called a conscious AI, [194,195]. We recognize that the analysis of a limited set of databases represents a limitation but we hope that this paper can be a useful tool for researchers to increase the debate on the frameworks underlying the various theories of consciousness, as noted by other authors (e.g., [187]). It could also be considered as a general framework from which the readers can start to analyze some arguments that are only cited in this review.

Finally, considering the different areas of study of the authors who wrote the analyzed articles, as well as the difficulty derived from the use of different technical languages, the probability that there are some theoretical aspects insufficiently treated in the summaries is high. To comply with the formatting guidelines, we had to limit the amount of information in the description of each theory, and this may have resulted in the above-mentioned limitations. Moreover, the Likert score attributed to each article in relation to the evaluation on how each dimension was debated in the text should not be intended as a qualitative score of the theories. For example, an article can debate many dimensions, such as the neural correlates that offer less “clear” messages on the structures involved in consciousness rather than an article that explicitly debated this point. The aim of this review was not to compare the different theories found or to discuss them in a critical manner (debating for one hypothesis or another one) but to synthesize them to provide a general overview on models that explain one of the most difficult topics known to humanity. Considering the limits of our work, this overview, overall, can be useful for readers and future researchers both to reflect on the variabilities that exist on the various topics related to consciousness in the scientific literature and to develop new ideas and collaborative research groups starting from the theories presented.

## 5. Conclusions

In conclusion, we found 29 theoretical models that described different perspectives on consciousness. Among the dimensions analyzed, the NCC was the most reported in the full-text collected and we found a very heterogeneous set of neural correlates of consciousness, ranging from the single electron to the whole brain. Moreover, we found fewer articles that focused on the development or implementation of new tools/strategy/markers for clinical practice in settings where consciousness levels were altered. Regarding the definition of consciousness, the review highlighted that the words “information”, “experience”, and “state” were often reported within definitions, and that several new terms/labels related to sub-categorization of consciousness (more than twenty terms) were also proposed. The results of this scoping review allows us to present the state-of-the-art research on consciousness studies and can offer a global vision on this issue for researchers all over the world. Because consciousness is one of the most ancient questions considered by human beings, this accomplishment is no small feat.

## Figures and Tables

**Table 1:** Consciousness definitions found in the collected articles.

Theory	Authors (year)	Main Definition	Other Terms/Subcategorization Related to Consciousness (Definitions Reported If Available)
**ADT**	LaBerge and Kasevich (2007) [18]	Consciousness is an activity that is extended in time and typically continues from the time we awake to the time we fall asleep.	- Background consciousness: some processes in the brain create “cognitive event that may be called ‘having an impression’ of something”. - Elevated consciousness for selected aspects of background consciousness is assumed to arise when sustained activity of a primary sensory area is sent to higher sensory areas, and where a selected part of the sensory scene is amplified by attentional activity controlled from the frontal lobes. - Foreground consciousness: the elevated attentional activity of a part of the sensory scene in higher sensory areas. - Content of consciousness: the simultaneous impressions from both foreground and background consciousness together;
**Agnati et al.’s proposal**	Cook (2008) [19]	nf	
	Agnati et al. (2012) [20]	Consciousness may be thought as the global result of integrative processes taking place at different levels of miniaturization in plastic mosaics	
**AIM**	Hobson (2009) [21]	Waking consciousness can be defined as the awareness of the external world, our bodies, and ourselves (including the awareness of our awareness) that humans experience when awake.	- Primary consciousness can be defined as simple awareness that includes perception and emotion. - Secondary consciousness depends on language and includes such features as self-reflective awareness, abstract thinking, volition, and metacognition.
**ART**	Grossberg (2007) [22]	nf	
	Grossberg (2017) [23]	Consciousness is not just a whir of information-processing.	- Core consciousness (see Damasio’s theory)
**AST**	Graziano and Kastner (2011) [24]	Consciousness is not an emergent property or a metaphysical emanation but is itself information computed by an expert system. […] Consciousness=awareness (i.e., a perceptual model of attention).	
**Bieberich’s theory**	Bieberich (2012) [25]	Consciousness determines what is perceived as reality.	
**COI**	Kriegel (2007) [26]	When a subject has a higher-order representation that is unified with the first-order representation it represents, their representational unity constitutes a conscious state. Consciousness determines what is perceived as reality.	
**CP**	Merker (2007) [27]	Consciousness may be regarded most simply as the “medium” of all possible experience. Consciousness as the state or condition presupposed by any experience whatsoever.	- Reflective consciousness: is one of many contents of consciousness available to creatures with sophisticated cognitive capacities; it is “a luxury of consciousness on the part of certain big-brained species, and not its defining property”; self-consciousness, unselfconsciously.
**CSS**	Berkovich-Ohana et al. (2014) [28]	Consciousness is an experienced property of mental states and processes, which is lost during a dreamless deep sleep, deep anesthesia, or coma	- Minimal self (MS): a self that is short of temporal extension and endowed with a sense of agency, ownership, and non-conceptual first-person content. - Narrative self (NS): it involves personal identity and continuity across time, as well as conceptual thought. - Core consciousness (CC): it supports the MS; its scope is in the here and now. - Extended consciousness (EC): it supports the NS and involves memory of past, imagination of future, and verbal thought. It deals with holding in mind, overtime, a multiplicity of neural patterns that describe the autobiographical self.
	Berkovich-Ohana et al. (2017) [29]	nf	
**Damasio’s theory**	Bosse et al. (2008) [30]	Proto-self: The proto-self is a coherent collection of neural patterns which map, moment by moment, the state of the physical structure of the organism in its many dimensions. Core consciousness (or feeling a feeling) is what emerges when the organism detects that its representation of its own body state (the proto-self) has been changed by the occurrence of the stimulus. Thus, it becomes (consciously) aware of the feeling, i.e., extended consciousness;	
**DCT**	Ward (2011) [31]	Phenomenal consciousness is generated by synchronized neural activity in the dendritic trees of dorsal thalamic neurons: a thalamic dynamic core.	Meta-consciousness and Self-consciousness are viewed as contents of consciousness that are built from recursive application of primary consciousness to conscious contents.
**EMI/CEMI**	McFadden (2007) [32]	Consciousness is that component of the brain’s electromagnetic information field that is downloaded to motor neurons and is thereby capable of communicating its state to the outside world. Consciousness is what electromagnetic field information feels like from the inside.	
	Pockett (2007) [33]	nf	
	Lewis and MacGregor (2010) [34]	Consciousness itself is a dispositional form of energy that may relate to physical forms of energy as the phase state of a gas relates in a material way to its liquid phase.	
**Gelepithis’ theory**	Gelepithis (2014) [35]	nf	
**Gurwitsch’s theory**	Yoshimi (2011) [36]	nf	
	Yoshimi and Vinson (2015) [37]	Consciousness is the totality of co-present data […] Any field of consciousness can be parsed into these three co-present domains of data: theme, thematic field, and margin.	Marginal consciousness: unattended data not relevant to the theme. Theme: data at the focus of attention organized according to Gestalt law. Thematic field: unattended data relevant to the theme.
**GWT/GNW**	Baars and Franklin (2007) [38]	Consciousness serves as a lookout function to spot potential dangers or opportunities so that there is a particularly close relationship between conscious content and significant sensory input.	
	Prakash et al. (2008) [39]	nf	
	Baars and Franklin (2009) [40]	nf	
	Raffone and Pantani (2010) [41]	nf	Phenomenal consciousness consists in phenomenally conscious states that are not cognitively accessible. Access consciousness represents a content information which is ‘broadcast’ in the GW.
	Dehaene and Changeux (2011) [42]	The availability of information is what we subjectively experience in a conscious state. “Conscious” is an ambiguous word. In its intransitive use (e.g., “the patient was still conscious”) it refers to the state of consciousness, also called wakefulness or vigilance, which is thought to vary continuously from coma and slow-wave sleep to full vigilance. In its transitive use (e.g., “I was not conscious of the red light”), it refers to conscious access to and/or conscious processing of a specific piece of information.	
	Sergent and Naccache (2012) [43]	It is possible to use subjective reports to probe the content of consciousness and therefore define any representation which is not reported by the subject as non-conscious, even when questioned about it, the existence of which can be demonstrated through behavioral and/or functional brain-imaging measures.	Phenomenal consciousness is a much larger domain of conscious contents than the one accessible through reports; perceptual consciousness; micro-consciousness
	Baars et al. (2013) [44]	Conscious experiences reflect a flexible “binding and broadcasting” function in the brain, which mobilize a large, distributed collection of specialized cortical networks and processes that are not conscious by themselves.	Sensory consciousness; perceptual consciousness.
	Dehaene et al. (2014) [45]	Conscious access is the process by which a piece of information becomes conscious content. Conscious processing refers to the various operations that can be applied to a conscious content (as when multiplying two numbers mentally). Conscious report is the process by which a conscious content can be described verbally or by various gestures. Such reportability remains the main criterion for whether a piece of information is or is not conscious, i.e., I can report something if and only if I am aware of it.	Self-consciousness is a particular instance of conscious accessibility where the conscious ‘spotlight’ is oriented toward internal states […] The state of consciousness, associated with fluctuations in wakefulness or vigilance, refers to the brain’s very ability to entertain stream of conscious contents.
	Bartolomei et al. (2014) [46]	The global availability of information through the workspace is what we subjectively experience as a conscious state.	
**HOT**	Lau (2007) [47]	Perceptual consciousness depends on the representation of the probability distributions that describe the behavior of the internal signal.	
	Lau and Rosenthal (2011) [48]	Consciousness consists in perceptual processes that occur with subjective experience, of which we are aware and about which we can report under normal circumstances. This contrasts with perceptual processes that occur without subjectivity, of which we are unaware and about which we cannot report. The term ‘conscious awareness’ can also apply to thoughts and volitional states of which one is subjectively aware	
	Friesen (2014) [49]	nf	
	Mehta and Mashour (2013) [50]	Consciousness is the story that our perceptual system tells us about the world. Any conscious state is a representation of what it is like to be in a conscious state, which is wholly determined by the content of that representation.	General consciousness: it pertains the levels of consciousness; - Specific consciousness pertains the content of consciousness
**IIT**	Balduzzi and Tononi (2008) [51]	Consciousness has to do with a system’s capacity to generate integrated information. This suggestion stems from considering two basic properties of consciousness: (i) each conscious experience generates a large amount of information by ruling out alternative experiences; (ii) the information is integrated, meaning it cannot be decomposed into independent parts.	
	Tononi (2008) [52]	Consciousness is integrated information and its quality is given by the informational relationships generated by a complex of elements.	The quantity of consciousness generated by a complex of elements is determined by the amount of integrated information it generates above and beyond its parts. The quality of consciousness is determined by the set of all the informational relationships its mechanisms generate.
	Tononi (2012) [53]	Consciousness is what vanishes every night when we fall into dreamless sleep and reappears when we wake up or when we dream. Consciousness is synonymous with experience.	
	Casali et al. (2013) [54]	Conscious experience is both differentiated (i.e., it has many specific features that distinguish it from a large repertoire of other experiences) and integrated (i.e., it cannot be divided into discrete, independent components).	
	Oizumi et al. (2014) [55]	An experience (i.e., consciousness) is thus an intrinsic property of a complex of elements in a state: how they constrain—in a compositional manner—its space of possibilities, in the past, and in the future.	
	Tononi and Koch (2015) [15]	Consciousness is a fundamental property possessed by physical systems having specific causal properties. Consciousness is a fundamental, observer-independent property that can be accounted for by the intrinsic cause–effect power of certain mechanisms in a state—how they give form to the space of possibilities in their past and their future. Consciousness is a fundamental property of certain physical systems, one that requires having real cause–effect power, specifically the power of shaping the space of possible past and future states in a way that is maximally irreducible intrinsically.	
	Tononi et al. (2016) [56]	Consciousness is subjective experience.	The quality or content of consciousness is identical to the form of the conceptual structure specified by the PSC. The quantity or level of consciousness corresponds to its irreducibility (integrated information Φ).
	Tsuchiya et al. (2016) [57]	Consciousness usually refers to either level or contents of consciousness.	The level of consciousness ranges from very high in the aroused awake state to low as in coma, vegetative states, deep dreamless sleep, and deep general anesthesia. At a given level of consciousness, every experiential moment contains various contents of consciousness. The contents of consciousness are synonymous with the other concepts such as qualia (its singular is a quale) or phenomenal consciousness.
**LRMB**	Wang (2012) [58]	Consciousness is a collective mental state of self-awareness that represents the bodily and mental status and their relations to the external environment, which is inductively generated or synthesized from the levels of metabolic homeostasis, unconsciousness, subconsciousness, and consciousness from the bottom-up. Consciousness is the basic characteristic of life and the mind, which is the state of being awareness of oneself, of perception to both internal and external worlds, and of responsive to one’s surroundings. Consciousness is a collective state at the perception layer of the 7-layer LRMB model, such as the sensation, action, memory, perception, metacognition, inference, and cognitive layers from the bottom-up. Consciousness is the sense of self and sign of life in natural intelligence. Consciousness is a collective and general state of advanced living organisms encompassing all cognitive attributes, which is generated based on the physiological structures, memory, and the brain’s power for acquiring and manipulating sensory and mental information.	- Subconsciousness
**MCTT**	Dalla Barba and Boissè (2010) [59]	Consciousness is not a generic and a specific dimension that then becomes the consciousness of its object. It is immediately conscious of something. We would also add that consciousness is always consciousness of something in a certain way. This means that consciousness takes a certain point of view of its object and of the same object consciousness can take various points of view.	Knowing consciousness (KC) describes what is usually referred to as semantic memory. KC is temporal since it is a synthesis of what I have been. At the same time, KC is also atemporal in the sense that the time of which it is made is unrecognizable. Temporal consciousness is an organized, original, and irreducible form of consciousness for addressing the world. Unlike KC, TC transcends the mere presence of the object to set it in time.
**Mesocircuit Hypothesis**	Schiff (2010) [60]	Consciousness is the combination of different components: arousal, which relates to behavioral and physiological observations and establishes a threshold level that allows other aspects of higher consciousness to occur; awareness and motivation, which presupposes the will to interact with the environment and therefore can be intense as a “tension towards something”.	
**Min’s Model**	Min (2010) [61]	Consciousness is a mental state embodied through TRN-modulated synchronization of thalamocortical networks. Consciousness consists of each mental unit, which is an individual thalamocortical looping mechanism, no matter what cognitive stages it involves. Consciousness is referred to as thalamocortical response modes controlled by the TRN and is embodied in the form of dynamically synchronized thalamocortical networks ready for upcoming attentional processes	Awareness: conscious perception of an attended mental representation by strengthening relevant neural networks through thalamocortical reiterating.
**NIH**	Yu and Blumenfeld (2009) [5]	Consciousness is the ability to maintain an alert state, attention, and awareness of self and environment	
**O’Doherty’s theory**	O’Doherty (2013) [62]	Consciousness represents the storage of past events for use in future situations and it is altered by external experience of the organism. Consciousness results from the gradual evolutionary development of the human information processing function. Consciousness is a phenomenon resulting from interactions between organisms rather than being located within an organism. Consciousness may consist of higher level of signal detection that has evolved through categorization and storage of past events.	
**PFT**	Morsella et al. (2015) [63]	Consciousness is a phenomenon associated with perceptual-like processing and interfacing with the somatic nervous system.	
**PToC**	Shannon (2008) [64]	Consciousness is the subjective experience and there are three types of consciousness: the sensed being, mental awareness, and meta-mentation.	Sensed being or sentience concerns the primitive and elementary aspect of consciousness that distinguishes the living from inanimate organisms. It has no specific context or structure; it is pervasive, and it is present during all our life. Mental awareness, typical of higher-order mammals, relates subjective experience that is distinct and differentiated, and the contents and forms of those experiences, such as mental images, ideations, flows of consciousness, and internal verbal monologues. Meta-mentation is the mind’s ability to take its own productions as object for further reflection. This type of ability has different manifestation like meta-observation, reflection, monitoring, and control.
**Quantum theories**	Klemm and Klink (2008) [65]	Consciousness is the capacity of a system to opt among presented alternatives.	Unreflected consciousness refers to conscious acts and meanings in their immediate experiential and direct givenness.
	Das (2009) [66]	Consciousness is a property of the Nambu-Goldtone bosons created by Yukawa coupling between the Namubu-Goldstone boson scalar field and the electron Dirac field.	
	Di Biase (2009) [67]	Consciousness is non-local information with a status equal to matter and energy.	
	Koehler (2011) [68]	nf	
	Argonov (2012) [69]	Consciousness in the phenomenal sense is a synonym of “subjective reality”, i.e., perception, volition, mental images, and emotions	
	Georgiev (2013) [6]	Consciousness is a collective term that refers to the subjective character of our mental states, and of our ability to experience or to feel. A conscious state is a state of experience.	
	Li (2013) [70]	nf	
	Hameroff and Penrose (2014) [71]	Consciousness consists of a sequence of discrete events, each being a moment of ‘objective reduction’ (OR) of a quantum state (according to the DP scheme), where it is taken that these quantum states exist as parts of quantum computations carried on primarily in neuronal microtubules. Such OR events would have to be ‘orchestrated’ in an appropriate way (Orch OR), for genuine consciousness to arise.	
	Hoffman and Prakash (2014) [72]	A conscious agents as a six tubules C = ((X,X), (G,G), P,D, A,N)), where: (X, X) and (G, G) are measurable spaces; P: W × X→[0, 1], D: X × G→[0, 1], A: G ×W→[0, 1] are mathematical formalism of Markoviank kernels, where N is an integer.	
	Kak et al. (2016) [73]	States of consciousness are mediated by languages and metalanguages with varying capacity or ability to recruit memories.	
	Sieb (2016) [74]	Conscious experience is defined as the direct observation of conscious events. A conscious event [...] is the fundamental entity of conscious experience (observed physical reality) represented by three coordinates of space and one coordinate of time in the space–time continuum: conscious event that consist of a set of qualia; conscious experience that is intimately tied to perception; postulated for conscious experience; conscious experience that is essentially an orientation in space and time.	
	Brabant (2016) [75]	nf	
**Reji Kumar’s theory**	Reji Kumar (2010) [76]	Human consciousness is built with models and these models are involved in the development of consciousness at a very basic level. As such, consciousness is subject to all advantages and limitations of models. Consciousness is the result of all information processing activities that occur in the mind.	
	Ahmad and Khan (2012) [77]	Consciousness is the core component of the mind. Consciousness can be defined as the spirit of consciousness and the transitivity of an information system. Transitivity can be defined as a certain correspondence between the system of information and outside matters. Consciousness is the final output of all information processing activities that occur in the mind.	
	Reji Kumar (2016) [78]	Consciousness is the result of all processes that occur in the brain.	
	Reji Kumar (2016) [79]	Consciousness is the totality of all effects of the functions of the brain and the supporting nervous system that produces the feeling of the objective world and the subjective mind or self	
**RPT**	Cleeremans et al. (2007) [80]	Consciousness is the brain’s theory about itself, gained through experience interacting with the world, and, crucially, with itself […] Conscious experience occurs if and only if an information processing system has learned about its own representations of the world […] Experience is, almost by definition (“what it feels like”), something that takes place not in any physical entity but rather only in special physical entities, namely cognitive agents.	
	Cleeremans (2008) [81]	nf	
**SPC**	Thagard and Stewart (2014) [82]	Consciousness is a neural process resulting from three mechanisms: representation by firing patterns in neural populations, binding of representations into more complex representations called semantic pointers, and competition among semantic pointers to capture the most important aspects of an organism’s current state.	

Note. The table enlists the qualitative data retrieved from each article included in the review grouped by theory (first column). The main definitions of consciousness are quoted from the original articles (please see Table S3 for other definitions of consciousness and related terms). nf = not found in the article analyses.

**Table 2:** Number of citations. The table shows how many times (second column) a specific term (first column) was used when referring to consciousness by theories (third column).

Terms	N. of Citations	Theory
**Activity**	4	ADT, DCT
**Awareness**	13	AIM, AST, HOT, LMRB, Mesocircuit Hypothesis, NIH, PToC
**Brain**	6	EMI/CEMI, GWT/GNW, LRMB, Reji Kumar’s theory, RPT
**Cognitive**	4	LRMB, Min’s Model, RPT
**Collective**	6	LRMB, Quantum theories
**Component**	4	EMI/CEMI, IIT, Mesocircuit Hypothesis, Reji Kumar’s theory
**Content**	7	GWT/GNW, HOT, IIT
**Events**	7	O’Doherty’s theory, Quantum theories
**Experience**	26	AIM, CP, CSS, GWT/GNW, HOT, IIT, O’Doherty’s theory, PToC, Quantum theories, RPT
**External**	4	AIM, LRMB, O’Doherty’s theory
**Feeling**	7	Damasio’s theory, Reji Kumar’s theory
**Field**	6	EMI/CEMI, Gurwitsch’s theory, Quantum theories
**Form**	4	EMI/CEMI, IIT, Min’s Model
**Function**	5	GWT/GNW, O’Doherty’s theory, Reji Kumar’s theory
**Information**	20	ART, AST, EMI/CEMI, GWT/GNW, IIT, LRMB, O’Doherty’s theory, Reji Kumar’s theory, RPT
**Integrated**	5	IIT
**Level**	6	Agnati et al.’s proposal, IIT, LRMB, Mesocircuit Hypothesis, O’Doherty’s theory, Reji Kumar’s theory
**Mental**	10	CSS, GWT/GNW, LRMB, Min’s Model, Quantum theories
**Mind**	5	LRMB, Reji Kumar’s theory
**Neural**	4	Damasio’s theory, DCT, SPC,
**Object**	5	MCTT, Quantum theories, Reji Kumar’s theory
**Organism**	7	Damasio’s theory, LRMB, O’Doherty’s theory, SPC
**Past**	5	IIT, O’Doherty’s theory
**Perception**	5	LRMB, Quantum theories
**Perceptual**	5	AST, GWT/GNW, HOT, PFT
**Physical**	7	AST, Damasio’s theory, EMI/CEMI, IIT, Quantum theories, RPT
**Possible**	5	CP, GWT/GNW, IIT
**Power**	4	IIT, LRMB
**Processes**	17	Agnati et al.’s proposal, CSS, GWT/GNW, HOT, Min’s Model, Reji Kumar’s theory
**Property**	9	AST, CSS, IIT, Quantum theories
**Reality**	4	Bieberich’s theory, COI, Quantum theories
**Representation**	12	COI, Damasio’s theory, GWT/GNW, HOT, RPT, SPC
**Something**	5	GWT/GNW, MCTT, Mesocircuit Hypothesis, RPT
**Space**	6	IIT, Quantum theories
**State**	28	CP, Damasio’s theory, EMI/CEMI, GWT/GNW, HOT, LRMB, Min’s Model, Quantum theories
**Subjective**	14	GWT/GNW, HOT, IIT, PToC, Quantum theories, Reji Kumar’s theory
**System**	11	AST, HOT, IIT, PFT, PToC, Reji Kumar’s theory, RPT,
**Thalamocortical**	4	Min’s Model
**World**	7	AIM, EMI/CEMI, HOT, LRMB, Reji Kumar’s theory, RPT

**Table 3:** Main neural correlates of consciousness reported in the various theories. In the second column, the percentage of theories referring to a specific neural structure is reported. The third column contains the name of theories that were reported for each neural structure. The fourth column indicates the percentage of theories that refer to either micro-, subcortical-, cortical-structures, or entire systems as neural correlates of consciousness.

Brain Structures	Theories Percentage Single Structure	Theories	Theories Percentage
**Micro-structures**			
Single Neuron	3.44%	Agnati’s theory	24%
Pyramidal neurons	6.89%	ADT, Bieberich’s Theory	
GABAergic inhibitory neurons	3.44%	Min’s model	
Periaqueductal gray neurons	3.44%	CP	
Astroglial-pyramidal cells	3.44%	CEMI	
Glia cells	3.44%	Agnati’s theory	
Dendritic cells	10.34%	ADT, Bieberich’s Theory, Quantum Theories	
Neural Microtubules	3.44%	Quantum Theories	
Ionic channels	10.34%	Agnati’s theory, Bieberich’s Theory, Quantum Theories	
Cortical minicolumns	3.44%	ADT	
**Cortical single structures**			
Prefrontal cortex (PFC)	24.13%	Agnati’s theory, CP, GWT, HOT, Min’s model, Quantum Theories, RPT	48%
Orbitofrontal areas	3.44%	ART	
Dorsolateral prefrontal cortex (dlPFC)	6.89%	COI, CSS	
Medial pre-frontal cortex (mPFC)	10.34%	AST, COI, CSS	
Cingulate cortex	13.79%	CSS, Damasio’s theory, GWT, NIH	
Anterior cingulate cortex	13.79%	Agnati’s theory, COI, CSS, GWT	
Experidorsal anterior cingulate cortex (dACC)	3.44%	CSS	
Medial cingulate gyrus	3.44%	CP	
Posterior cingulate cortex (PCC)	3.44%	CSS	
Precuneus	6.89%	CSS, NIH	
Medial frontal cortex	3.44%	NIH	
Supplementary motor area (SMA)	3.44%	CSS	
Premotor cortex	6.89%	CSS, GWT	
Parietal cortex	10.34%	CSS, GWT	
Superior parietal lobule	3.44%	CSS	
Inferior parietal lobule	3.44%	CSS	
Posterior parietal cortex (PPC)	10.34%	CP, HOT, quantum theories	
Temporoparietal junction	6.89%	AST, CSS	
Temporal lobe	17.24%	ART, AST, CSS, GWT, MCTT	
Occipital cortex	3.44%	GWT	
**Subcortical single structures**			
Basal ganglia	10.34%	ART, CP, quantum theories	52%
Nucleus accumbens	3.44%	Agnati’s theory	
Claustrum	3.44%	Agnati’s theory	
Insula	6.89%	Agnati’s theory, CSS	
Amygdala	3.44%	ART	
Hippocampus	17.24%	ART, CSS, GWT, quantum theories, RPT	
Cerebellum	10.34%	GWT, LRMB, Reji Kumar’s theory	
Thalamus	24.13%	Agnati’s theory, CP, Damasio’s theory, DCT, GWT, HOT, LRMB	
Anterior Pretectal nucleus	3.44%	DCT	
Medial thalamus nuclei	3.44%	NIH	
Thalamic reticular nucleus	6.89%	DCT, Min’s model	
Pulvinar	3.44%	DCT	
Hypothalamus	6.89%	ART, CP	
Midbrain	3.44%	CP	
Superior colliculus	6.89%	CP, Damasio’s theory	
Ventral tegmental area	3.44%	Agnati’s theory	
Brain stem	3.44%	NIH	
Raphe nuclei	3.44%	Agnati’s theory	
	**Brain systems/regions**		
Cortico-thalamic system	17.24%	ART, AIM, IIT, Mesocircuit hypothesis, quantum Theories	28%
Fronto-parietal areas	6.89%	CSS, GWT	
Sensory cortical areas	3.44%	GWT	
Perceptual regions	3.44%	PFT	
Limbic system	3.44%	AIM	
Striate regions	3.44%	GWT	

## Data Availability

This is a review article. Data available at https://doi.org/10.5281/zenodo.4671955.

## Supplementary Materials

The following are available online at https://www.mdpi.com/article/10.3390/brainsci11050535/s1. Table S1: Search strategy; Table S2: Maximum score obtained in each dimension by the theories analyzed; Table S3: Secondary definitions and definitions of related terms and features of consciousness. Table S4: Abstracts of each theory analyzed.
