# Supplementary material for: Theoretical Models of Consciousness: A Scoping Review
Source: Brain Sci. 2021 Apr 24;11(5):535. doi: 10.3390/brainsci11050535 (PMC8146510; doi:10.3390/brainsci11050535)
Supplement: Supplementary file 1 [file brainsci-11-00535-s001.zip › brainsci-1199943-SI.pdf]

Supplemental materials

Theoretical Models of Consciousness: A Scoping Review

Davide Sattin[1,2,*], Francesca G. Magnani[1], Laura Bartesaghi[1], Milena Caputo[1], Andrea V. Fittipaldo[3], Mario Picozzi[4], Matilde Leonardi[1]

1 Neurology, Public Health, Disability Unit – Scientific Department, Fondazione IRCCS Istituto Neurologico Carlo Besta, Milan, Italy
2 Experimental medicine and medical humanities-PhD program, Biotechnology and Life Sciences Department and Center for Clinical Ethics, Insubria University, Varese, Italy
3 Oncology Department, Mario Negri Institute for Pharmacological Research IRCCS, Milan, Italy
4 Center for Clinical Ethics, Biotechnology and Life Sciences Department, Insubria University, Varese, Italy.
* Correspondence: davide.sattin@istituto-besta.it ; Tel.: +39 02 2394.2709

Table S1. *Search strategy*

**MEDLINE**	**N°**
**Main Search strategy**	
((((((theor*[Title/Abstract]) OR ("theoretical model"[Title/Abstract] OR "theoretical models"[Title/Abstract] OR "theoretic model"[Title/Abstract] OR "theoretic models"[Title/Abstract])) OR "Models, Theoretical"[Mesh])))) AND (((consciousness[MeSH Terms]) OR "consciousness"[Title/Abstract]) OR conscious*[Title/Abstract])	4635
**Combination of the main search strategy AND search strategy for each single dimension**	
(((((((consciousness[MeSH Terms]) OR conscious*[Title/Abstract])))))) AND ((((((("neural correlates of consciousness"[Title/Abstract]) OR neuroanatomy[Title/Abstract]) OR "neural differentation and integration") OR "brain mapping"[Title/Abstract]) OR physiology[Title/Abstract])))	504
(((((((consciousness[MeSH Terms]) OR conscious*[Title/Abstract]))))) AND (((("Cognitive Function" OR "Cognitive Functions" OR "Function, Cognitive" OR "Functions, Cognitive")) OR ("Cognitive Sciences" OR "Science, Cognitive" OR "Neuroscience, Cognitive"))) AND (Attention* OR Concentration OR Concentrations OR language* OR memor*)	163
(((((("Quantitative Evaluation"[Title/Abstract] OR "Evaluation, Quantitative"[Title/Abstract] OR "Evaluations, Quantitative"[Title/Abstract] OR "Quantitative Evaluations"[Title/Abstract]) OR Evaluation*[Title/Abstract] OR "Evaluation Indexes"[Title/Abstract] OR "Indexes, Evaluation"[Title/Abstract]) AND Humans[Mesh])) AND (((((((consciousness[MeSH Terms]) OR conscious*[Title/Abstract])))))))	2081
((((consciousness[MeSH Terms]) OR conscious*[Title/Abstract]))) AND ((perception*[Title/Abstract] OR "Autonomic Nervous Systems"[Title/Abstract] OR "Nervous System, Autonomic"[Title/Abstract] OR "Nervous Systems, Autonomic"[Title/Abstract] OR "System, Autonomic Nervous"[Title/Abstract] OR "Systems, Autonomic Nervous"[Title/Abstract] OR "Visceral Nervous System"[Title/Abstract] OR "Nervous System, Visceral"[Title/Abstract] OR "Nervous Systems, Visceral"[Title/Abstract] OR "System, Visceral Nervous"[Title/Abstract] OR "Systems, Visceral Nervous"[Title/Abstract] OR "Visceral Nervous Systems"[Title/Abstract] OR "Vegetative Nervous System"[Title/Abstract] OR "Nervous System, Vegetative"[Title/Abstract] OR "Nervous Systems, Vegetative"[Title/Abstract] OR "System, Vegetative Nervous"[Title/Abstract] OR "Systems,	2434

Vegetative Nervous"[Title/Abstract] OR "Vegetative Nervous Systems"[Title/Abstract] OR "Olfactory Perceptions"[Title/Abstract] OR "Perception, Olfactory"[Title/Abstract] OR "Perceptions, Olfactory"[Title/Abstract] OR "Perception, Visual"[Title/Abstract] OR "Perceptions, Visual"[Title/Abstract] OR "Visual Perceptions"[Title/Abstract] OR "Auditory Perceptions"[Title/Abstract] OR "Perception, Auditory"[Title/Abstract] OR "Perceptions, Auditory"[Title/Abstract] OR "Perception, Taste"[Title/Abstract] OR "Perceptions, Taste"[Title/Abstract] OR "Taste Perceptions"[Title/Abstract] OR "Gustatory Perception"[Title/Abstract] OR "Gustatory Perceptions"[Title/Abstract] OR "Perception, Gustatory"[Title/Abstract] OR "Perceptions, Gustatory"[Title/Abstract] OR "Perception, Touch"[Title/Abstract] OR "Perceptions, Touch"[Title/Abstract] OR "Touch Perceptions"[Title/Abstract] OR "Tactile Perception"[Title/Abstract] OR "Perception, Tactile"[Title/Abstract] OR "Perceptions, Tactile"[Title/Abstract] OR "Tactile Perceptions"[Title/Abstract]))	
((((consciousness[MeSH Terms]) OR conscious*[Title/Abstract]) AND Humans[Mesh])) AND (((subjectivity OR "first person" OR "self report" OR "self psychology" OR "self evaluation")))	539
**EMBASE**	
Queryconsciousness:ti,ab AND 'theory' NEAR/4 'consciousness' OR consciousness NEAR/4 ('model' OR 'theoretical model' OR 'theoretical study' OR 'theoretical studies' OR 'theoretical models') AND [english]/lim AND [humans]/lim	521
**SCOPUS**	
(key (consciousness) or title-abs-key (conscious*) and title-abs-key (((model*) or (theor*))) and abs (human)) and (exclude (subjarea , "agri") or exclude (subjarea , "envi") or exclude (subjarea , "busi") or exclude (subjarea , "econ") or exclude (subjarea , "eart") or exclude (subjarea , "mate") or exclude (subjarea , "ceng") or exclude (subjarea , "vete") or exclude (subjarea , "dent"))	4603
**WOS**	
(TS=(consciousness) OR TS=(conscious)) AND (TS=(theoretical model*) OR TS=(theoretic model*) OR TS=(model*) OR TS=(theor*))	3879
**PsychINFO**	

(((((((ab(consciousness) OR ab(conscious*) OR ti(consciousness) OR ti(conscious*) AND ab((theoretic model* OR theoretical model* OR theor*))	4671
Filters: “Human” AND “Language”	

Note: The table reports the search strategies used to perform the present review. The numbers on the right represent the total number of article found adopting a specific strategy.

Table S2. *Maximum score obtained in each dimension by the theories analyzed.*

Theory	*Authors*	*Year*	*Neural correlates*	*Association between consciousness and other cognitive functions*	*Translation from theory to clinical practice*	*Quantitative measures of consciousness*	*Consciousness, sensory processes and the autonomic nervous system*	*Subjectivity*
**ADT**	LaBerge and Kasevich	2007	5	3	0	3	4	3
**Agnati et al.'s proposal**	Agnati et al.	2012	5	1	0	0	3	1
	Cook	2008	5	0	1	0	0	5
**AIM**	Hobson	2009	4	0	0	0	2	1
**ART**	Grossberg	2007	4	5	0	0	3	0

	Grossberg	2017	4	5	0	0	5	2
**AST**	Graziano and Kastner	2011	3	4	0	0	4	2
**Bieberich ’s theory**	Bieberich	2012	5	3	0	3	2	0
**COI**	Kriegel	2007	5	2	0	0	5	0
**CP**	Merker	2007	5	2	0	0	3	0
**CSS**	Berkovich-Ohana et al.	2014	4	5	0	0	2	1

	Berkovich-Ohana et al.	2017	1	2	0	0	4	1
**Damasio's theory**	Bosse et al.	2008	2	0	0	0	1	2
**DCT**	Ward	2011	5	3	5	0	2	1
**EMI/CEMI**	McFadden	2007	0	0	0	0	0	0
	Pockett	2007	0	1	0	1	0	0
	Lewis & MacGregor	2010	4	3	0	0	2	1
**Gelepithis' theory**	Gelepithis	2014	0	1	0	0	1	2

**Gurwitsch's theory**	Yoshimi	2011	0	0	0	4	0	1
	Yoshimi and Vinson	2015	4	4	0	0	1	2
**GWT/GNW**	Dehaene and Changeux	2011	4	2	3	0	3	1
	Dehaene et al.	2014	2	2	0	0	3	0
	Baars and Franklin	2007	0	4	0	0	2	0

Prakash et al.	2008	5	3	0	0	1	0
Baars and Franklin	2009	0	4	0	0	0	0
Raffone and Pantani	2010	4	5	0	4	4	0
Sergent and Naccache	2012	5	1	1	0	3	3
Baars et al.	2013	5	5	0	0	4	1
Bartolomei et al.	2014	4	1	3	0	0	0

	Lau	2008	0	2	0	0	4	0
**HOT**	Lau and Rosenthal	2011	4	2	0	0	4	3
	Friesen	2014	0	0	0	0	5	1
	Mehta and Mashour	2013	5	2	0	0	4	0
	Balduzzi and Tononi	2008	2	0	0	5	0	2

	Tononi	2008	4	0	0	4	0	4
	Tononi	2012	3	0	0	4	0	0
**IIT**	Casali et al.	2013	2	0	5	4	0	1
	Oizumi et al.	2014	2	0	0	4	0	1
	Tononi and Koch	2015	4	1	2	0	0	2
	Tononi et al.	2016	5	0	0	0	0	1

	Tsuchiya et al.	2016	2	0	0	1	0	2
**LRMB**	Wang	2012	4	4	1	5	4	2
**MCTT**	Dalla Barba and Boissè	2010	3	2	0	0	0	2
**Mesocircuit Hypothesis**	Schiff	2010	4	1	4	0	1	0
**Min's Model**	Min	2010	5	3	0	0	5	0
	Yu and							

**NIH**	Blumenfeld	2009	4	2	0	0	0	0
**O’Doherty’s theory**	O'Doherty	2013	0	5	0	0	0	1
**PToC**	Shanon	2008	0	2	0	2	2	3
	Klemm & Klink	2008	0	1	0	0	0	3
	Das	2009	0	0	0	2	0	0
**Quantum**	Di Biase	2009	1	1	0	0	0	0

**theories**							
	Koehler	2011	0	0	0	4	1	0
	Argonov	2012	3	0	0	0	0	0
	Baars and Edelman	2012	1	1	0	0	0	2
	Georgiev	2013	0	0	0	0	0	2
	Li	2013	0	0	0	4	0	1
	Hameroff & Penrose	2014	4	2	0	3	0	1

	Arushi et al.	2016	1	3	0	1	1	1
	Sieb	2016	3	3	0	4	2	1
	Brabant	2016	0	0	0	0	0	1
	Reji Kumar	2010	0	1	0	0	1	4
**Reji Kumar's theory**	Ahmad and Khan	2012	0	1	0	5	3	4
	Reji Kumar	2016	0	0	0	5	0	5

	Reji Kumar	2017	1	3	0	3	0	2
**RPT**	Cleeremans et al.	2007	0	0	0	0	1	3
	Cleeremans	2008	2	0	0	0	1	5
**SPC**	Thagard and Stewart	2014	4	2	0	4	1	2

Notes: The table reports the scores achieved by each article in the dimensional model. The first column represents the theory each article belongs to. In the second and third columns, authors and year of publication are reported. The following columns refer to the six dimensions considered in the dimensional. model. ADT: Apical dendrite theory; AIM: Activation/information/mode-synthesis hypothesis; ART: Adaptive Resonance Theory; AST: Attention Schema Theory; COI: Cross-order integration theory of consciousness; CP: Centrencephalic proposal; CCS: Consciousness state space model; DCT: Dynamic Core Theory; EM/CEMI: Electromagnetic field Hypothesis /Consciousness Electromagnetic Information field theory; GWT: Global workspace theory; GNW: Global Neural Workspace; HOT/FOR: Higher-order theories of consciousness/First order representational theory;

IIT: Integrated information theory; LRMB: Layered Reference Model of the Brain; MCTT: Memory Consciousness and Temporality Theory; NIH: Network inhibition hypothesis; PToC: Psychological theory of consciousness; Q-Theories: Quantum Theories; RPT: Radical plasticity thesis; SPC: Semantic pointer competition theory of consciousness.

Theory	*Authors (year)*	*Related terms/features*
**ADT**	LaBerge and Kasevich (2007)	Thinking self; Feeling self
**Agnati et al.'s proposal**	Agnati et al. (2012)	Sentience; subjective awareness; self-awareness
	Cook (2008)	Sentience; neural sentience
**AIM**	Hobson (2009)	--
**ART**	Grossberg (2007)	*Resonant state:* A resonant state emerges through sustained feedback between the attended bottom-up signal pattern and the active top-down expectation as they reach a consensus between what is expected and what is there in the outside world;
	Grossberg (2017)	--
**AST**	Graziano and Kastner (2011)	--
**Bieberich's theory**	Bieberich (2012)	- *Sentyons* are conscious particles;<br>- The *endospace* is a space embedding a set of elements perceived in consciousness
**COI**	Kriegel (2007)	--

**CP**	Merker (2007)	A *conscious mode* of functioning is dependent upon quite specific neural arrangements creating interfaces of particular kinds between specific domains of neural function, rather than a result of a general increase in informational capacity or complexity achieved by expansion of a structural substrate which below a certain size does not support consciousness.
**CSS**	Berkovich-Ohana et al. (2014)	Consciousness would be inconceivable without temporality; self-consciousness
	Berkovich-Ohana et al. (2017)	--
**Damasio's theory**	Bosse et al. (2008)	--
**DCT**	Ward (2011)	*Metaconsciousness and Self-consciousness* are viewed as contents of consciousness that are built from recursive application of primary consciousness to conscious contents
**EMI/CEMI**	McFadden (2007)	--
	Pockett (2007)	--
	Lewis & MacGregor (2010)	--
**Gelepithis' theory**	Gelepithis (2014)	--
**Gurwitsch's theory**	Yoshimi (2011)	--
	Yoshimi and Vinson (2015)	*Self-awareness* is our awareness of our own inner thoughts, a stream of ideas, concepts and inner speech constantly running through our

		minds; ''*somatic*'' self: 'There is no moment in conscious life when we are completely<br>unaware of our bodily posture, of the fact that we are walking, standing, sitting, lying down, etc.;
**GWT/GNW**	Dehaene and Changeux (2011)	Preconscious
	Dehaene et al. (2014)	--
	Baars and Franklin (2007)	*Conscious cognition; Functionally conscious agent*: is an autonomous agent which implements a Global Workspace Theory for the role of consciousness in a Working Model of Cognition (WMC), to facilitate the performance of real-world tasks
	Prakash et al. (2008)	--
	Baars and Franklin (2009)	--
	Raffone and Pantani (2010)	--
	Sergent and Naccache (2012)	--
	Baars et al. (2013)	Conscious cognition
	Bartolomei et al. (2014)	--
	Lau (2008)	--

**HOT**	Lau and Rosenthal (2011)	Metacognition; Sensory metacognition
	Friesen (2014)	--
	Mehta and Mashour (2013)	--
**IIT**	Balduzzi and Tononi (2008)	--
	Tononi (2008)	--
	Barros (2010)	--
	Tononi (2012)	--
	Casali et al. (2013)	--
	Oizumi et al. (2014)	--
	Tononi and Koch (2015)	--
	Tononi et al. (2016)	Phenomenal consciousness
	Tsuchiya et al. (2016)	Phenomenal consciousness
**LRMB**	Wang (2012)	Unconsciousness; Subconsciousness
		Past consciousness; Present consciousness; Future consciousness

**MCTT**	Dalla Barba and Boissè (2010)	
**Mesocircuit Hypothesis**	Schiff (2010)	--
**Min's Model**	Min (2010)	*Awareness*: Conscious perception of an attended mental representation by strengthening relevant neural networks through thalamocortical reiterating;
**NIH**	Yu and Blumenfeld (2009)	--
**O'Doherty's theory**	O'Doherty (2013)	--
**PToC**	Shanon (2008)	--
**Quantum theories**	Klemm & Klink (2008)	- *Subjectivity* means the principle of the individualized origin point and owner of experiences as "mine." Subjectivity has both empirical and transcendental dimensions;<br>- The *empirical subject* is the immediate subject of first-order empirical experiences, in which "I" relate to things, other persons, my own mental states, or even reflective objects such as truth, goodness, or beauty;<br>- The *transcendental subject* is the subjective principle of the unity of consciousness over time in relating to its own first-order experiences. The transcendental<br>I relates not to first-order things, persons, and so forth but rather as a second-order possibility to its own relations to things (for example, as theoretical, practical, or aesthetic relations). It is the condition of the possibility

		of unifying diverse conscious experiences into a continuous narrative account that is "mine"
	Das (2009)	--
	Di Biase (2009)	--
	Koehler (2011)	--
	Argonov (2012)	--
	Baars and Edelman (2012)	--
	Georgiev (2013)	--
	Li (2013)	--
	Hameroff & Penrose (2014)	--
	Kak et al. (2016)	--
	Sieb (2016)	- *Qualia* are qualities or properties perceived or experienced by a person;
	Brabant (2016)	--
**Reji Kumar's theory**	Reji Kumar (2010)	--
	Ahmad and Khan (2012)	--
	Reji Kumar (2016)	*Model* represents the preliminary mental representation of data and information
	Reji Kumar (2016)	--
	Cleeremans et al. (2007)	--

**RPT**	Cleeremans (2008)	--
**SPC**	Thagard and Stewart (2014)	--

Notes: ADT: Apical dendrite theory; AIM: Activation/information/mode-synthesis hypothesis; ART: Adaptive Resonance Theory; AST: Attention Schema Theory; COI: Cross-order integration theory of consciousness; CP: Centrencephalic proposal; CCS: Consciousness state space model; DCT: Dynamic Core Theory; EM/CEMI: Electromagnetic field Hypothesis /Consciousness Electromagnetic Information field theory; GWT: Global workspace theory; GNW: Global Neural Workspace; HOT/FOR: Higher-order theories of consciousness/First order representational theory; IIT: Integrated information theory; LRMB: Layered Reference Model of the Brain; MCTT: Memory Consciousness and Temporality Theory; NIH: Network inhibition hypothesis; PToC: Psychological theory of consciousness; Q-Theories: Quantum Theories; RPT: Radical plasticity thesis; SPC: Semantic pointer competition theory of consciousness.

Table S4 – Abstract of each theory analyzed.

Theory	*Authors (year)*	*Synthesis*
**ADT**	LaBerge and Kasevich (2007)	The Apical Dendrite Theory focused on the input-processing which sustained consciousness. The theory analyzed two main circuits of the minicolumn (ministructures composed of major pyramidal neurons, stellate neurons, inhibitory neurons and axons): The Shell and the Axis circuit. The theory assumed that sustained activity in a primary sensory area provides the ongoing activities of a background consciousness for that particular sense. Elevated consciousness for selected aspects of background consciousness is assumed to arise when sustained activity of a primary sensory area is sent to higher sensory areas, where a selected part of the sensory scene is amplified by attentional activity controlled by the frontal lobes.
**Agnati et al.'s proposal**	Agnati et al. (2012)	The authors mainly referred to Cook and Sevush's idea of a single-neuron theory of consciousness. The authors claimed that both cognition and sensitivity could be present in the glial cells (other than neurons), which could represent in this way a prerequisite for consciousness and subjectivity. Furthermore, it is postulated the existence of the so-called functional modules representing microcircuits of neurons and astrocytes well-organized in specific patterns to carry out a specific process. These functional modules are characterized by both vertical and horizontal organizations. Regarding neural correlates, the authors focused on the key role of the thalamus in receiving and re-directing both sensory and cortical input
	Cook (2008)	--
**AIM**	Hobson (2009)	This hypothesis gives particular attention to the cortical-thalamic and limbic systems, which are active in waking and REM sleep, for conscious experience. The author suggested that REM is one of the first signals indicating the brain is preparing itself for gradual development of integrative work among different functions, one of which is consciousness. The theory considered three factors that may be computerized: Activation (A), Input–output gating (I) and Modulation (M).
**ART**	Grossberg (2007)	--
	Grossberg (2017)	The author started from the already existing Adaptive Resonance Theory (ART), where resonance consists of dynamical states represented by the amplification of neuronal firing and interactions between top-down and bottom-up processes. The resonant states are bind together to build coherent world's representations. The bottom-up categories and the top-down expectations are bound only if there

		is a match between them. The matching between bottom-up external input and the top-down expectation results in resonant states.
**AST**	Graziano and Kastner (2011)	Authors affirmed that consciousness is not an emergent property but is itself information computed by an expert system. For this theory, awareness is a form of meta-social intelligence reconstructing someone else's thoughts, beliefs, or emotions, which also determines the state of someone else's attention. According to the theory, when a Subject constructs a perceptual model of someone else's attention (e.g. "the person A is aware of X"), he/she also computed second information like "I am aware of X".
**Bieberich's theory**	Bieberich (2012)	In the author's view, the binding mechanism is at the base of reality perception, and it should be operated at the single neuron level. However, in the neural space there would be only a summation process without any integration mechanism leading to conscious perception. By assuming a refractal structure and by applying a downscale computation, it would be possible to describe both the macro (neural network) and the micro-neural structures. These mechanisms define the Recurrent Fractal Networks that are supposed to underlie the emergence of consciousness.
**COI**	Kriegel	One of the COI assumptions is that psychological events and states are representations in the brain and that some of these representations can be conscious whilst others can remain non-conscious, but we can envisage both a conscious and non-conscious representation of the same stimulus. Conscious states arise from the integration, or unification (two representations become a single representation. The author identified three levels: (i) a floor-level representation which determines the specific contents of consciousness, (ii) a higher-order representation of that floor-level representation and (iii) the functional integration of these two representations. According to COI, the second and third elements of the triad, the functional integration with the higher-order representation, constitutes the correlate of consciousness as such and not the content of consciousness.
**CP**	Merker (2007)	The theory postulated the existence of the so-called "triangle selection" encompassing (i) target selection, (ii) action selection and (iii) motivational ranking. It is assumed that when anyone is conscious, the above-mentioned interaction happens in an ego-centric reference frame which could be biased by the motivation. The neural structures involved in such interaction are represented by a system ranging from the colliculus to the brainstem system. By using Merker's words "*the way this simulation is structured constitutes a conscious mode of function*".
**CSS**	Berkovich-Ohana et al.	The Consciousness State Space (CSS) represents a three-dimensional neurophenomenological model composed of three dimensions, namely Time, Awareness and Emotion. The consciousness is fragmented into two different but interrelated categories: the first one

	(2014)	is the Core Consciousness (CC) which is related to the here and now and the second one is represented by the Extended Consciousness (EC) which is related to both episodic and prospective memory, as well as to verbal thoughts. These two categories are organized in an embedded way as the CC represents a ‘low-order’ category being strongly related to the body-sense, whilst the EC is far away from the body and it is related to higher-order cognitive processes. Furthermore, each category is organized along a continuum for each of the three above-mentioned dimensions. The awareness dimension extended from an absence of the awareness to a full-awareness state (included in the EC category) passing through a sensory and subliminal bodily awareness (included in the CC category).
	Berkovich-Ohana et al. (2017)	--
**Damasio’s theory**	Bosse et al. (2008)	The authors took into account three components as conceptualized by Damasio: (i) emotion as neural representation; (ii) feeling as body change perception; (iii) core consciousness as the detection of the body change after a stimulus has occurred. In order to create a simulation model of Damasio’s theory, the authors referred to the Temporal Trace Language which allows computing a change of the state given some temporal parameters. By using this model, an emotion is the result of external stimulation which is detected and it induces an internal representation which, in turn, prepares the body to act. The feeling is related to two different dynamics in the model: the “body loop” and the “as if body loop”. The first one represents the real change in the body, the second one represents a cognitive change related to the body instead of a real change in the body. The concept of “core consciousness” (i.e. feeling a feeling) and “extended consciousness” were then explained in that perspective.
**DCT**	Ward (2011)	The DCT focused on the synchronous firing of large cortical neuronal population. However, differently from other theories, the author affirmed that neither a specific cerebral structure nor a single specific kind of neural activity is both necessary and sufficient to explain the conscious states, because the theory assumes the existence of consciousness thanks to a distributed neural activity with a central role of the synchronized neural activity in cortico-thalamic circuits. A novelty in this theory is represented by the distinction of two different neuronal populations within the thalamus, the “core neurons” and the “matrix neurons” that integrate information deriving from the cortical computation allowing consciousness.
**EMI/CEMI**	McFadden (2007)<br>Pockett (2007)<br>Lewis & MacGregor (2010)	The basic idea of this group of theories is that when any neuron receives a signal from upstream neurons, synaptic transmitters stimulate ion pumps that cause the membrane to become more or less negatively polarized. This massive membrane depolarization generates an electromagnetic field perturbation that will influence the probability of firing of adjacent neurons, so the electromagnetic fields of adjacent neurons will not be discrete but form a complex

		overlapping field composed of the superposition of the fields of millions of neurons. The key feature of this superposition field is that it is capable of integrating vast quantities of information into a single physical system and hence it can account for the binding of information for consciousness generation.
**Gelepithis' theory**	Gelepithis (2014)	Gelepithis formulated a theory based on seven definitions that compose a definitional system.
**Gurwitsch's theory**	Yoshimi (2011)<br>Yoshimi and Vinson (2015)	Gurwitsch proposed a theory of the "*articulation of the total field of consciousness and the patterns and forms in which co-present data are organized with respect to each other*". According to the theory, any field of consciousness is organized according to the following three domains: Theme, Thematic Field and Marginal Consciousness. Yoshimi and Vinson, in 2015, proposed an extension of this theory focusing on three points: peripheral experience, "predictive relevance" and ''variable size'', showing as phenomenological and connectionism approaches could be applied to solve some mathematical problems to explain consciousness generation.
**GWT/GNW**	Dehaene and Changeux (2011)<br>Dehaene et al. (2014)<br>Baars and Franklin (2007)<br>Prakash et al. (2008)<br>Baars and Franklin (2009)<br>Raffone and Pantani (2010)<br>Sergent and Naccache (2012)<br>Baars et al. (2013)<br>Bartolomei et al. (2014)	The core idea grounding the Global Workspace Theory (GWT) is the presence of an interaction between bottom-up and top-down attentional modulation mechanisms which, throughout a broadcasting process, allows a specific percept/event to became conscious. The (GNW) hypothesis proposes that associative perceptual, motor, attention and memory areas interconnect to create a unified space where information is shared in a global blackboard and it is sent back to lower-level processors. The neuronal workspace is anatomically formed by different structures, from cortical pyramidal cells in layers II/III with long-range excitatory axons, particularly dense in prefrontal, cingulate and parietal regions, together with the relevant thalamocortical circuits. For GNW, what we subjectively experience as conscious access is the selection, amplification and global broadcasting of a single piece of information selected for its salience or relevance to current goals, while the rest of the neurons composing the GNW are inhibited.
	Lau (2008)<br>Lau and Rosenthal (2011)	The representational theories affirmed that consciousness is directly linked to "mental representations" rather than to the physical state. There are several varieties of HOR but they share common neural correlates at the level of the prefrontal and the parietal cortex, which

**HOR/FOR**	Friesen (2014)<br>Mehta and Mashour (2013)	assumed a crucial role for the creation of high representation linking together different information. Conversely, the FOR performs a distinction between the post-sensory structures, corresponding to the same neural correlates of consciousness in the HOR which determine the so-called "general consciousness" and the sensory-structures corresponding to specific neural sensory areas and sensory thalamic nuclei and deputed to the so-called "specific consciousness", which corresponds to the contents of consciousness.
**IIT**	Balduzzi and Tononi (2008)<br>Tononi (2008)<br>Barros (2010)<br>Tononi (2012)<br>Casali et al. (2013)<br>Oizumi et al. (2014)<br>Tononi and Koch (2015)<br>Tononi et al. (2016)<br>Tsuchiya et al. (2016)	The IIT claims that consciousness is determined by its causal properties and it is, therefore, an intrinsic, fundamental property of any physical system. IIT explains both the quantity and the quality of consciousness. The first one corresponds to the amount of integrated information generated by a system and it corresponds to its irreducibility; the second one is identified by the set of informational relationships generated within that complex system. Quality of consciousness could be geometrically represented within a space, called qualia space, in which it is possible to see the complex of informational relationship that determines it. The IIT addresses the hard problem of consciousness by proposing a set of phenomenological axioms, ontological postulates and identities.
**LRMB**	Wang (2012)	The authors developed the theory of the Layered Reference Model of the Brain which affirmed that the hierarchical life functions of the brain can be divided into two categories. The first one is the *subconscious* life functions encompassing the layers of sensation, action, memory and perception. These functions are inherited, fixed and relatively matured when a person was born. The *conscious* functions include the layers of metacognition, inference and cognitive functions sustained. In the model, the thalamus and the cerebellum are considered key structures for consciousness.
**MCTT**	Dalla Barba and Boissè (2010)	The authors redefined the concept of consciousness in the framework of the Memory Consciousness and Temporality Theory. For them consciousness is not a generic and aspecific dimension but it is immediated. They distinguished between the *temporal* and the *knowing consciousness* postulating different components.
**Mesocircuit**	Schiff (2010)	The "mesocircuit hypothesis" considers the thalamus (and globus pallidus) as the key structure for conscious states due to its widespread direct and indirect connections with both brainstem and frontal areas. This theory accounted for both evidence from patients

**Hypothesis**		with disorders of consciousness following thalamic damages, from neuromodulation studies and the mechanisms underlying the paradoxical effect of some drugs in restoring the level of consciousness thanks to its inhibitory action on specific brain areas.
**Min's Model**	Min (2010)	According to the present theory, the Thalamic Reticular Nucleus could act as a mediator for the signals derived from the other thalamic nuclei to the cortex and *vice versa*, exercising in this way a sort of "control" over the thalamic activity in a feed-forward fashion. As the TRN is equipped with GABAergic neurons, it is plausible that it is responsible for the synchronization of the cortical and thalamic neural activities given rise to information processing and, hence, to consciousness, suggesting mechanisms that allow to explain consciousness generation and the unity of consciousness.
**NIH**	Yu and Blumenfeld (2009)	The network Inhibition Hypothesis is a theory that aims to explain consciousness starting from the evidence reported in the studies on epilepsy. The idea is that the so-called "conscious system" is analogous to other systems by involving different cortical and sub-cortical structures and that the consciousness system must include neural areas that govern functions like attention (i.e. a prerequisite for consciousness), awareness and alert state. The NIH hypothesized that the loss of consciousness is a secondary effect due to the dysregulation of the networks described above and that focal limbic seizures propagate to subcortical structures involved in the arousal systems.
**O'Doherty's theory**	O'Doherty (2013)	In O'Doherty's view, consciousness can be conceptualized as the product of an interaction between an individual and the environment, rather than something located inside the individual himself/herself. Within this view, memory is considered a prerequisite for language development, as it allows for information storage. Finally, qualia are considered as a key feature of consciousness since an individual cannot be conscious in the absence of an experience/stimulation and its storage.
**PToC**	Shanon (2008)	Originally, Shanon identified three types of consciousness (identifying consciousness as the region of the internal subjective experience), hierarchically ordered and interdependent among them. They form a well-integrated system, which is defined as a "tripartite system". The system of consciousness encloses different regions and each one presents different levels: the Self, concerning personal identity, the World which is inherent in the relationship between knowledge and the world and, finally, the Temporality that concerns the temporal part of the experience.
	Klemm & Klink (2008)	In this section are described different theories. However, almost all affirmed that consciousness depends in some way on the processes of

**Quantum theories**	Das (2009)<br>Koehler (2011)<br>Argonov (2012)<br>Baars and Edelman (2012)<br>Genovesi (2012)<br>Georgiev (2013)<br>Li (2013)<br>Hameroff & Penrose (2014)<br>Kak et al. (2016)<br>Sieb (2016)<br>Brabant (2016)	the general nature of quantum computations. The fundamental problems these authors highlighted are: i) the material world in classical deterministic physical theories is causally close (if the brain states produce conscious experiences, then these experiences cannot possibly have an effect upon brain dynamics and ii) the illusion that consciousness is able to make a choice between alternatives such as a "free" system, since the deterministic frameworks make it an illusion produced by our subjective brain. Different perspectives are then presented in the text.
**Reji Kumar's theory**	Reji Kumar (2010)<br>Ahmad and Khan (2012)<br>Reji Kumar (2016)<br>Reji Kumar (2016)	Reji Kumar suggested a model where consciousness is the result of the information processing taking place in the mind which, in turn, consists of information accepting, processing and generating. The information processing has been assumed similar among individuals, however, what subjectively changes is the information accepting. The present theory grounded on four different axioms: (i) A model represents a piece of information; (ii) the mind compares and classifies different models; (iii) new models can be created starting from the existing models; (iv) the mind attributes a value/preference to each model. The author attributed a pivotal role to the memory by assuming the existence of the temporary memory which receives information from both the outside world and the individuals' inside and a permanent memory where the information is stored after their processing and integration according to α-, β- and γ- models. When information is associated with an entity, within the β- models, the perception is subjective and so conscious.
**RPT**	Cleeremans et al. (2007)<br>Cleeremans (2008)	The author proposed the radical plasticity thesis of consciousness by taking into account the subjective experience as a core component. The individual creates some representations based on the external input and the metarepresentations inform the individual of his internal status by taking into account the representations previously created. The subjective experience takes place only if a certain system learned its representations. As a consequence, the consciousness is the

		awareness of both external and internal status.
**SPC**	Thagard and Stewart (2014)	The phenomenon of consciousness has been studied by the Semantic Pointer Competition (SPC) through three different hypotheses. The first one supposes that consciousness is the result of a process, within the brain, that springs from neural mechanisms, the second hypothesis identifies the representation by patterns of firing in neural populations, binding of these representations into semantic pointers and competition among semantic pointers" and The third hypothesis assumes that consciousness derives from an interactive competition between semantic pointers after which the winner indicators will determine the qualitative aspects of experience (i.e. qualia). The different kinds of consciousness depend on the difference in the complexity and the extent of the three mechanisms that generate experiences.

References

Agnati, L. F., Guidolin, D., Cortelli, P., Genedani, S., Cela-Conde, C., & Fuxe, K. (2012). Neuronal correlates to consciousness. the Hall of Mirrors metaphor describing consciousness as an epiphenomenon of multiple dynamic mosaics of cortical functional modules. *Brain Research*, *1476*, 3–21. https://doi.org/10.1016/j.brainres.2012.01.003

Ahmad, F., & Khan, Q. (2012). Can mathematical cognition formulate consciousness? *ICOSST 2012 - 2012 International Conference on Open Source Systems and Technologies, Proceedings*, 7–11. https://doi.org/10.1109/ICOSST.2012.6472846

Argonov, V. Y. (2012). Neural correlate of consciousness in a single electron: radical answer to "quantum theories of consciousness." *NeuroQuantology*, *10*(2), 276–285. Retrieved from www.neuroquantology.com

Aston-Jones, G., & Bloom, F. E. (1981). Activity of norepinephrine-containing locus coeruleus neurons in behaving rats anticipates fluctuations in the sleep-waking cycle. *Journal of Neuroscience*. https://doi.org/10.1523/jneurosci.01-08-00876.1981

Baars, B J, & Franklin, S. (2007). An architectural model of conscious and unconscious brain functions: Global Workspace Theory and IDA. *Neural Networks : The Official Journal of the International Neural Network Society*, *20*(9), 955–961. https://doi.org/S0893-6080(07)00161-X [pii]

Baars, B J, Ramsoy, T. Z., & Laureys, S. (2003). Brain, conscious experience and the observing self. *Trends in Neurosciences*, *26*(12), 671–675. https://doi.org/S0166-2236(03)00334-5 [pii]

Baars, Bernard J. (1997). In the theatre of consciousness: Global workspace theory, A rigorous scientific theory of consciousness. *Journal of Consciousness Studies*.

Baars, Bernard J., & Franklin, S. (2009). Consciousness is computational: The LIDA model of global workspace theory. *International Journal of Machine Consciousness*, *1*(1), 23–32. https://doi.org/10.1142/S1793843009000050

Baars, Bernard J., Franklin, S., & Ramsoy, T. Z. (2013). Global workspace dynamics: Cortical "binding and propagation" enables conscious contents. *Frontiers in Psychology*, *4*(MAY). https://doi.org/10.3389/fpsyg.2013.00200

Baars, Bernard J. (1993). *A cognitive theory of consciousness*. Cambridge University Press.

Balduzzi, D., & Tononi, G. (2008). Integrated information in discrete dynamical systems: Motivation and theoretical framework. *PLoS Computational Biology*, *4*(6). https://doi.org/10.1371/journal.pcbi.1000091

Bartolomei, F., McGonigal, A., & Naccache, L. (2014). Alteration of consciousness in focal epilepsy: the global workspace alteration theory. *Epilepsy & Behavior : E&B*, *30*, 17–23. https://doi.org/10.1016/j.yebeh.2013.09.012 [doi]

Bell, J. S. (1966). On the problem of hidden variables in quantum mechanics. *Reviews of Modern Physics*, *38*(3), 447–452. https://doi.org/10.1103/RevModPhys.38.447

Berkovich-Ohana, A., & Glicksohn, J. (2014). The consciousness state space (CSS)-a unifying model for consciousness and self. *Frontiers in Psychology*, *5*(APR). https://doi.org/10.3389/fpsyg.2014.00341

Berkovich-Ohana, A., & Wittmann, M. (2017). *A typology of altered states according to the consciousness state space (CSS) model: A special reference to subjective time*. Retrieved from https://www.researchgate.net/publication/316254551

Bieberich, E. (2012). Introduction to the Fractality Principle of Consciousness and the Sentyon Postulate. *Cognitive Computation*, *4*(1), 13–28. https://doi.org/10.1007/s12559-011-9104-5

Block, N. (2007). Consciousness, accessibility, and the mesh between psychology and neuroscience. *The Behavioral and Brain Sciences*, *30*(5–6), 481–548. https://doi.org/10.1017/S0140525X07002786 [doi]

Blouw, P., Solodkin, E., Thagard, P., Manuscript, C. E.-U., Of, U., & 2012, U. (2014). Concepts as semantic pointers: A theory and computational model. *Unpublished Manuscript, University of Waterloo*.

Blumenfeld, H. (2012). Impaired consciousness in epilepsy. *The Lancet Neurology*, *11*(9), 814–826.

https://doi.org/10.1016/S1474-4422(12)70188-6

Blumenfeld, H., McNally, K. A., Vanderhill, S. D., Paige, A. L. B., Chung, R., Davis, K., … Spencer, S. S. (2004). Positive and negative network correlations in temporal lobe epilepsy. *Cerebral Cortex*, *14*(8), 892–902. https://doi.org/10.1093/cercor/bhh048

Bohm, D. (1980). *Wholeness and the implicate order*. Retrieved from https://scholar.google.it/scholar?hl=it&as_sdt=0%2C5&q=wholeness+and+the+implicate+order&btnG=&oq=wholeness+and+the

Bohm, D., & Hiley, B. (1993). *The Undivided Universe-an Ontological Interpretation of Quantum Theory*. Retrieved from http://adsabs.harvard.edu/abs/1993Natur.366..420B

Bosse, T., Jonker, C. M., & Treur, J. (2008). Formalisation of Damasio's theory of emotion, feeling and core consciousness. *Consciousness and Cognition*, *17*(1), 94–113. https://doi.org/10.1016/j.concog.2007.06.006

Carruthers, P. (2009). How we know our own minds: The relationship between mindreading and metacognition. *Behavioral and brain sciences*, 32(2), 121-138.

Casali, A. G., Gosseries, O., Rosanova, M., Boly, M., Sarasso, S., Casali, K. R., … Massimini, M. (2013). A theoretically based index of consciousness independent of sensory processing and behavior. *Science Translational Medicine*, *5*(198), 198ra105. https://doi.org/10.1126/scitranslmed.3006294 [doi]

Castaigne, P., Lhermitte, F., Buge, A., Escourolle, R., Hauw, J. J., & Lyon-Caen, O. (1981). Paramedian thalamic and midbrain infarcts: Clinical and neuropathological study. *Annals of Neurology*, *10*(2), 127–148. https://doi.org/10.1002/ana.410100204

Cleeremans, A. (2007). Consciousness: the radical plasticity thesis. *Progress in Brain Research*, *168*, 19–33. https://doi.org/10.1016/S0079-6123(07)68003-0

Cleeremans, A., Timmermans, B., & Pasquali, A. (2007). Consciousness and metarepresentation: A computational sketch. *Neural Networks*, *20*(9), 1032–1039. https://doi.org/10.1016/j.neunet.2007.09.011

Cook, N. D. (2008, May 15). The neuron-level phenomena underlying cognition and consciousness: Synaptic activity and the action potential. *Neuroscience*, Vol. 153, pp. 556–570. https://doi.org/10.1016/j.neuroscience.2008.02.042

Cul, A. Del, Baillet, S., & Dehaene, S. (2007). Brain dynamics underlying the nonlinear threshold for access to consciousness. *PLoS Biology*, *5*(10), e260. https://doi.org/07-PLBI-RA-0389 [pii]

Dalla Barba, G. D., & Boissé, M. F. (2010). Temporal consciousness and confabulation: Is the medial temporal lobe temporal? *Cognitive Neuropsychiatry*, *15*(1–3), 95–117. https://doi.org/10.1080/13546800902758017

Damasio, A. R. (1996). The somatic marker hypothesis and the possible functions of the prefrontal cortex. *Philosophical Transactions of the Royal Society of London.Series B, Biological Sciences*, *351*(1346), 1413–1420. https://doi.org/10.1098/rstb.1996.0125 [doi]

Damasio, A. (1999). *The feeling of what happens: Body and emotion in the making of consciousness*. New York: Houghton Mifflin Harcourt.

Damasio, AR. (2012). *Self comes to mind: Constructing the conscious brain*. Vintage, New York.

Dayan, P., Abbott, L., & Abbott, L. (2001). *Theoretical neuroscience: computational and mathematical modeling of neural systems*. Cambridge: MIT Press.

De Lange, F. P., Jensen, O., & Dehaene, S. (2010). Accumulation of evidence during sequential decision making: The importance of top-down factors. *Journal of Neuroscience*, *30*(2), 731–738. https://doi.org/10.1523/JNEUROSCI.4080-09.2010

de Lange, F. P., van Gaal, S., Lamme, V. A. F., & Dehaene, S. (2011). How Awareness Changes the Relative Weights of Evidence During Human Decision-Making. *PLoS Biology*, *9*(11), e1001203. https://doi.org/10.1371/journal.pbio.1001203

Dehaene, S, & Changeux, J. P. (2005). Ongoing spontaneous activity controls access to consciousness: a neuronal model for inattentional blindness. *PLoS Biology*, *3*(5), e141. https://doi.org/04-PLBI-RA-0755R3

[pii]

Dehaene, S, & Changeux, J. P. (2011). Experimental and theoretical approaches to conscious processing. *Neuron*, *70*(2), 200–227. https://doi.org/10.1016/j.neuron.2011.03.018 [doi]

Dehaene, S, Charles, L., King, J. R., & Marti, S. (2014). Toward a computational theory of conscious processing. *Current Opinion in Neurobiology*, *25*, 76–84. https://doi.org/10.1016/j.conb.2013.12.005 [doi]

Dehaene, Stanislas, Changeux, J. P., & Naccache, L. (2011). The global neuronal workspace model of conscious access: From neuronal architectures to clinical applications. *Research and Perspectives in Neurosciences*. https://doi.org/10.1007/978-3-642-18015-6_4

Dehaene, Stanislas, & Naccache, L. (2001). Towards a cognitive neuroscience of consciousness: Basic evidence and a workspace framework. *Cognition*, *79*(1–2), 1–37. https://doi.org/10.1016/S0010-0277(00)00123-2

DeWitt, B. (1973). *The many worlds interpretation of quantum mechanics* (B. DeWitt & N. Graham, Eds.). Retrieved from https://scholar.google.it/scholar?hl=it&as_sdt=0%2C5&q=the+many+worlds+interpretation+of+quantum+mechanics&btnG=&oq=the+many+worlds+interpretation+of+quantum+mechani

Draguhn, A., Traub, R. D., Schmitz, D., & Jefferys, J. G. R. (1998). Electrical coupling underlies high-frequency oscillations in the hippocampus in vitro. *Nature*, *394*(6689), 189–192. https://doi.org/10.1038/28184

Dretske, F. (1995). *Naturalizing the Mind*. Cambridge, MA: Bradford/MIT Press.

Edelman, G. M. (1992). *Bright Air, Brilliant Fire: On The Matter Of The Mind*. Retrieved from https://www.britannica.com/topic/Bright-Air-Brilliant-Fire-On-

Edelman, G., & Tononi, G. (2000). *A Universe of Consciousness*. New York: Basic Books.

Eliasmith, C., & Anderson, C. (2004). *Neural engineering: Computation, representation, and dynamics in*

neurobiological systems. Retrieved from https://books.google.it/books?hl=it&lr=&id=J6jz9s4kbfIC&oi=fnd&pg=PR17&dq=Neural+engineering:+Computation,+representation+and+dynamics+in+neurobiological+systems&ots=2fsMp9LOeA&sig=NUTniTAoV37tp0wNqcUHJb6zQVU

Frankfurt, H. (1987). Equality as a Moral Ideal. *Ethics*. https://doi.org/10.1086/292913

Fridman, E. A., & Schiff, N. D. (2014, December 1). Neuromodulation of the conscious state following severe brain injuries. *Current Opinion in Neurobiology*, Vol. 29, pp. 172–177. https://doi.org/10.1016/j.conb.2014.09.008

Gelepithis, P. A. M. (2001). A concise comparison of selected studies of consciousness. *Cognitive Systems*, *5*(4), 373–392.

Gelepithis, P. A. M. (2014). A novel theory of consciousness. *International Journal of Machine Consciousness*, *6*(2), 125–139. https://doi.org/10.1142/S1793843014400150

Gennaro, R. (2011). *The Consciousness Paradox: Consciousness, Concepts, and Higher-order Thoughts (Representation and Mind)*.

Georgiev, D. (2013). Quantum No-Go Theorems and Consciousness. *Axiomathes*, *23*(4), 683–695. https://doi.org/10.1007/s10516-012-9204-1

Gilbert, C. D., & Sigman, M. (2007, June 7). Brain States: Top-Down Influences in Sensory Processing. *Neuron*, Vol. 54, pp. 677–696. https://doi.org/10.1016/j.neuron.2007.05.019

Gödel, K. (1931). Über formal unentscheidbare Sätze der Principia Mathematica und verwandter Systeme I. *Monatshefte Für Mathematik Und Physik*, *38*(1), 173–198. https://doi.org/10.1007/BF01700692

Graziano, M. S. A., & Kastner, S. (2011, June). Awareness as a perceptual model of attention. *Cognitive Neuroscience*, Vol. 2, pp. 125–127. https://doi.org/10.1080/17588928.2011.585237

Grossberg, S. (2007). Consciousness CLEARS the mind. *Neural Networks*, *20*(9), 1040–1053.

https://doi.org/10.1016/j.neunet.2007.09.014

Grossberg, S. (2017, March 1). Towards solving the hard problem of consciousness: The varieties of brain resonances and the conscious experiences that they support. *Neural Networks*, Vol. 87, pp. 38–95. https://doi.org/10.1016/j.neunet.2016.11.003

Gurwitsch, A. (1964). *Field of consciousness.* (Duquesne University Press., Ed.). Pittsburgh, PA; US.

Gurwitsch, A. (1985). *Marginal consciousness* (In P. Wrer; O. U. Press, Ed.). Ohio.

Hameroff, S. (2001). Consciousness, the brain, and spacetime geometry. *Annals of the New York Academy of Sciences*, *929*, 74–104.

Hameroff, S., & Penrose, R. (2014). Consciousness in the universe: a review of the "Orch OR" theory. *Physics of Life Reviews*, *11*(1), 39–78. https://doi.org/10.1016/j.plrev.2013.08.002 [doi]

Hobson, J. A., & McCarley, R. W. (1977). The brain as a dream state generator: an activation-synthesis hypothesis of the dream process. *American Journal of Psychiatry*, *134*(12), 1335–1348. https://doi.org/10.1176/ajp.134.12.1335

Hobson, J. Allan. (2009, November). REM sleep and dreaming: Towards a theory of protoconsciousness. *Nature Reviews Neuroscience*, Vol. 10, pp. 803–814. https://doi.org/10.1038/nrn2716

Husserl, E. (1964). *The phenomenology of internal time-consciousness, trans*. James Churchill. The Hague: Martinus Nijhoff.

Jones, E. G. (2002). Thalamic circuitry and thalamocortical synchrony. *Philosophical Transactions of the Royal Society B: Biological Sciences*, *357*(1428), 1659–1673. https://doi.org/10.1098/rstb.2002.1168

Jonker, C. M., & Treur, J. (1998). *Compositional Verification of Multi-Agent Systems: a Formal Analysis of Pro-activeness and Reactiveness*. https://doi.org/10.1007/3-540-49213-5_13

Kak, A., Gautam, A., & Kak, S. (2016). A three-layered model for consciousness states. *NeuroQuantology*,

14(2), 166–174. https://doi.org/10.14704/nq.2016.14.2.935

Kak, S. (2013). Biological memories and agents as quantum collectives. *NeuroQuantology*, *11*(3), 391–398. Retrieved from www.neuroquantology.com391

Kant, I. (1787). *Critique of pure reason* (P. Guyer & A. Wood, Eds.). Cambridge University Press.

Kochen, S., & Specker, E. (1967). The problem of hidden variables in quantum mechanics. *Journal of Mathematics and Mechanics*, *17*(1), 59–87.

Koehler, G. (2011). Q-Consciousness: Where is the Flow? *Nonlinear Dynamics, Psychology, and Life Sciences*, *15*(3), 335–357.

Kriegel, U. (2007). A cross-order integration hypothesis for the neural correlate of consciousness. *Consciousness and Cognition*, *16*(4), 897–912. https://doi.org/10.1016/j.concog.2007.02.001

LaBerge, D., & Kasevich, R. (2007). The apical dendrite theory of consciousness. *Neural Networks*, *20*(9), 1004–1020. https://doi.org/10.1016/j.neunet.2007.09.006

Lambert, N., Chen, Y. N., Cheng, Y. C., Li, C. M., Chen, G. Y., & Nori, F. (2013). Quantum biology. *Nature Physics*, *9*(1), 10–18. https://doi.org/10.1038/nphys2474

Lau, H., & Rosenthal, D. (2011, August). Empirical support for higher-order theories of conscious awareness. *Trends in Cognitive Sciences*, Vol. 15, pp. 365–373. https://doi.org/10.1016/j.tics.2011.05.009

Lee, K. H., Meador, K. J., Park, Y. D., King, D. W., Murro, A. M., Pillai, J. J., & Kaminski, R. J. (2002). Pathophysiology of altered consciousness during seizures: Subtraction SPECT study. *Neurology*, *59*(6), 841–846. https://doi.org/10.1212/WNL.59.6.841

Lewis, E. R., & MacGregor, R. J. (2010, June). A natural science approach to consciousness. *Journal of Integrative Neuroscience*, Vol. 9, pp. 153–191. https://doi.org/10.1142/S0219635210002202

Li, J. (2013). A Timeless and Spaceless Quantum Theory of Consciousness. *NeuroQuantology*, *11*(3). Retrieved

from www.neuroquantology.com431

MacDonald, C. J., Lepage, K. Q., Eden, U. T., & Eichenbaum, H. (2011). Hippocampal "time cells" bridge the gap in memory for discontiguous events. *Neuron*, *71*(4), 737–749. https://doi.org/10.1016/j.neuron.2011.07.012

McFadden, J. (2002a). Synchronous Firing and Its Influence on the Brain's Electromagnetic Field. *Journal of Cons*, *9*, 23–50.

McFadden, J. (2002b). The Conscious Electromagnetic Information (Cemi) Field Theory: The Hard Problem Made Easy? *Journal of Consciousness Studies*, *9*, 45–60.

McFadden, J. (2007). Conscious electromagnetic (CEMI) field theory. *NeuroQuantology*, *5*(3), 262–270.

Mehta, N., & Mashour, G. A. (2013). General and specific consciousness: A first-order representationalist approach. *Frontiers in Psychology*. https://doi.org/10.3389/fpsyg.2013.00407

Mensky, M. (2011). Mathematical models of subjective preferences in quantum concept of consciousness. *NeuroQuantology*, *9*, 614–620.

Merker, B. (2007). Consciousness without a cerebral cortex: A challenge for neuroscience and medicine. *Behavioral and Brain Sciences*, *30*(1), 63–81. https://doi.org/10.1017/S0140525X07000891

Min, B. K. (2010). A thalamic reticular networking model of consciousness. *Theoretical Biology and Medical Modelling*, *7*(1). https://doi.org/10.1186/1742-4682-7-10

Mountcastle, V. B. (1957). Modality and topographic properties of single neurons of cat's

somatic sensory cortex. *Journal of Neurophysiology*, *20*(4), 408–434. https://doi.org/10.1152/jn.1957.20.4.408

Nagel, T. (1974). What Is It Like to Be a Bat? *The Philosophical Review*, *83*(4), 435. https://doi.org/10.2307/2183914

Natsoulas, T. (1981). Basic problems of consciousness. *Journal of Personality and Social Psychology*, *41*(1), 132–178. https://doi.org/10.1037/0022-3514.41.1.132

Nidditch, P. (1975). *John Locke: An essay concerning human understanding*. Retrieved from https://philpapers.org/rec/NIDJLA-3

O'Doherty, F. (2013). A Contribution to Understanding Consciousness: Qualia as Phenotype. *Biosemiotics*, *6*(2), 191–203. https://doi.org/10.1007/s12304-012-9140-x

O'Keefe, J., & Dostrovsky, J. (1971). The hippocampus as a spatial map. Preliminary evidence from unit activity in the freely-moving rat. *Brain Research*, *34*(1), 171–175. https://doi.org/10.1016/0006-8993(71)90358-1

O'Keefe, John. (1976). Place units in the hippocampus of the freely moving rat. *Experimental Neurology*, *51*(1), 78–109. https://doi.org/10.1016/0014-4886(76)90055-8

O'Reilly, R., & Munakata, Y. (2000). *Computational explorations in cognitive neuroscience: Understanding the mind by simulating the brain*. Retrieved from https://books.google.it/books?hl=it&lr=&id=BLf34BFTaIUC&oi=fnd&pg=PR19&dq=Computational+explorations+in+cognitive+neuroscience&ots=_bHrxmw7Eq&sig=8nKbyHXeyVZLKJZTYsjIVSaJOKI

Oizumi, M., Albantakis, L., & Tononi, G. (2014). From the phenomenology to the mechanisms of consciousness: Integrated Information Theory 3.0. *PLoS Computational Biology*, *10*(5), e1003588. https://doi.org/10.1371/journal.pcbi.1003588 [doi]

Payne, D., & Wenger, M. (1998). *Cognitive psychology*. Houghton Mifflin College Division.

Penfield, W., & Jasper, H. (1954). *Epilepsy and the functional anatomy of the human brain*. Retrieved from https://psycnet.apa.org/record/1955-01377-000

Penrose, R. (1994). *Shadows of the Mind: A Search for the Missing Science of Consciousness*. Retrieved from https://scholar.google.it/scholar?hl=it&as_sdt=0%2C5&q=Penrose+R.+Shadows+of+the+Mind%3A+A+Search+for+the+Missing+Science+of+Consciousness.+&btnG=

Penrose, Roger. (1996). On gravity's role in quantum state reduction. *General Relativity and Gravitation*. https://doi.org/10.1007/BF02105068

Petkov, V. (2010). *Minkowski spacetime: A hundred years later*. Retrieved from https://link.springer.com/content/pdf/10.1007/978-90-481-3475-5.pdf

Pockett, S. (2007). Difficulties with the electromagnetic field theory of consciousness: an update. *NeuroQuantology*, *5*(3), 271–275.

Pompeiano, O. (1967). The neurophysiological mechanisms of the postrual and motor events during desynchronized sleep. *Research Publications - Association for Research in Nervous and Mental Disease*, *45*, 351–423. Retrieved from http://www.ncbi.nlm.nih.gov/pubmed/4867152

Prakash, R., Prakash, O., Prakash, S., Abhishek, P., & Gandotra, S. (2008). Global workspace model of consciousness and its electromagnetic correlates. *Annals of Indian Academy of Neurology*, *11*(3), 146–153. https://doi.org/10.4103/0972-2327.42933

Raffone, A., & Pantani, M. (2010). A global workspace model for phenomenal and access consciousness. *Consciousness and Cognition*, *19*(2), 580–596. https://doi.org/10.1016/j.concog.2010.03.013 [doi]

Reji Kumar, K. (2010). Modeling of Consciousness: Classification of Models. *Advanced Studies in Biology*, *2*(3), 141–146.

Reji Kumar, K. (2016a). Mathematical modeling of consciousness: A foundation for information processing. *Proceedings of IEEE International Conference on Emerging Technological Trends in Computing, Communications and Electrical Engineering, ICETT 2016*. https://doi.org/10.1109/ICETT.2016.7873719

Reji Kumar, K. (2016b). Mathematical modeling of consciousness: Subjectivity of mind. *Proceedings of IEEE International Conference on Circuit, Power and Computing Technologies, ICCPCT 2016*. https://doi.org/10.1109/ICCPCT.2016.7530157

Robinson, W. (2012). Epiphenomenalism. In E. Zalta, U. Nodelman, & C. Allen (Eds.), *Stanford encyclopedia of philosophy*. Stanford: Stanford University.

Rosenthal, D. M. (1990). A theory of consciousness. In *ZiF technical report 40*. Bielfield, Germany: Reprinted in Block et al. (1997).

Rosenthal, D. M. (2002). Explaining consciousness. In D. J. Chalmers (Ed.), *Philosophy of*

mind: Classical and contemporary readings (pp. 406–421). Retrieved from https://scholar.google.com/scholar_lookup?title=Explaining consciousness&publication_year=2002&author=D.M. Rosenthal

Rosenthal, D. M. (2005). *Consciousness and Mind*. Retrieved from https://scholar.google.it/scholar?hl=it&as_sdt=0%2C5&q=D.M.+Rosenthal+Consciousness+and+Mind+Clarendon+Press+%282005%29&btnG=

Rounis, E., Maniscalco, B., Rothwell, J. C., Passingham, R. E., & Lau, H. (2010). Theta-burst transcranial magnetic stimulation to the prefrontal cortex impairs metacognitive visual awareness. *Cognitive Neuroscience*. https://doi.org/10.1080/17588921003632529

Schiff, N. D. (2010, January). Recovery of consciousness after brain injury: a mesocircuit hypothesis. *Trends in Neurosciences*, Vol. 33, pp. 1–9. https://doi.org/10.1016/j.tins.2009.11.002

Schiff, N. D. (2013). Central thalamic deep brain stimulation for support of forebrain arousal regulation in the minimally conscious state. In *Handbook of Clinical Neurology* (Vol. 116, pp. 295–306). https://doi.org/10.1016/B978-0-444-53497-2.00024-3

Schiff, N. D. (2016). Central thalamic deep brain stimulation to support anterior forebrain mesocircuit function in the severely injured brain. *Journal of Neural Transmission, 123*(7), 797–806. https://doi.org/10.1007/s00702-016-1547-0

Schrödinger, E. (1926). An undulatory theory of the mechanics of atoms and molecules. *Physical Review, 28*(6), 1049–1070. https://doi.org/10.1103/PhysRev.28.1049

Schwiedrzik, C. M., Singer, W., & Melloni, L. (2011). Subjective and objective learning effects dissociate in space and in time. *Proceedings of the National Academy of Sciences of the United States of America*. https://doi.org/10.1073/pnas.1009147108

Sergent, C., Baillet, S., & Dehaene, S. (2005). Timing of the brain events underlying access to consciousness during the attentional blink. *Nature Neuroscience*, *8*(10), 1391–1400. https://doi.org/nn1549 [pii]

Sergent, C., & Naccache, L. (2012). Imaging neural signatures of consciousness: "What", "When", "Where" and "How" does it work? *Archives Italiennes de Biologie*, *150*(2–3), 91–106. https://doi.org/10.4449/aib.v150i2.1270

Sevush, S. (2006). Single-neuron theory of consciousness. *Journal of Theoretical Biology*, *238*(3), 704–725. https://doi.org/10.1016/j.jtbi.2005.06.018

Shanon, B. (2008). A psychological theory of consciousness. *Journal of Consciousness Studies*, *15*(5), 5–47. Retrieved from https://www.ingentaconnect.com/content/imp/jcs/2008/00000015/00000005/art00001

Sieb, R. (2017). Four-Dimensional Consciousness. *Activitas Nervosa Superior*, *59*(2), 43–60. https://doi.org/10.1007/s41470-017-0008-x

Sieb, R. A. (2016). Human Conscious Experience is Four-Dimensional and has a Neural Correlate Modeled by Einstein's Special Theory of Relativity. *NeuroQuantology*, *14*, 630–644. https://doi.org/10.14704/nq.2016.14.3.983

Smith, R. (1993). *Psychology*. St. Paul, Minnesota: West Publishing Company.

Sternberg, R. J. (1998). In search of the human mind. In *Harcourt Brace College Publishers* (2nd ed.). Retrieved from https://books.google.com/books?id=BVolKydwWGUC&q=Sternberg+1998&dq=Sternberg+1998&hl=en&sa=X&ved=0ahUKEwikkcPY1tLXAhWG2RoKHbgLCbkQ6AEILDAB

Tegmark, M. (2000). Importance of quantum decoherence in brain processes. *Physical Review E - Statistical Physics, Plasmas, Fluids, and Related Interdisciplinary Topics*, *61*(4), 4194–4206. https://doi.org/10.1103/PhysRevE.61.4194

Thagard, P., & Stewart, T. C. (2014). Two theories of consciousness: Semantic pointer competition vs. information integration. *Consciousness and Cognition*, *30*, 73–90. https://doi.org/10.1016/j.concog.2014.07.001

Tononi, G. (2012). Integrated information theory of consciousness: an updated account. *Archives Italiennes de Biologie*, Vol. 150, pp. 293–329. https://doi.org/10.4449/aib.v149i5.1388

Tononi, G., & Koch, C. (2015). Consciousness: here, there and everywhere? *Philosophical Transactions of the Royal Society B: Biological Sciences*, *370*(1668), 20140167–20140167. https://doi.org/10.1098/rstb.2014.0167

Tononi, G, & Edelman, G. M. (1998). Consciousness and complexity. *Science (New York, N.Y.)*, *282*(5395), 1846–1851.

Tononi, G. (2008). Consciousness as integrated information: a provisional manifesto. *The*

Biological Bulletin, *215*(3), 216–242. https://doi.org/10.2307/25470707

Tononi, GS, & Edelman, G. (1998). Consciousness and the integration of information in the brain. In H. H. Jasper, L. Descarries, V. F. Castellucci, & S. Rossignol (Eds.), *Consciousness: at the frontiers of neuroscience* (pp. 245–279). Retrieved from https://philpapers.org/rec/TONCAT

Treisman, A., & Schmidt, H. (1982). Illusory conjunctions in the perception of objects. *Cognitive Psychology*, *14*(1), 107–141. https://doi.org/10.1016/0010-0285(82)90006-8

Tye, M. (1995). *Ten problems of consciousness: A representational theory of the phenomenal mind*. Retrieved from https://books.google.com/books?hl=it&lr=&id=L6QMP6LaPckC&oi=fnd&pg=PR11&dq=Tye,+M.+1995.+Ten+Problems+of+Consciousness.+Cambridge,+MA:+MIT+Press&ots=scIfLDlfax&sig=Cox7oa-PgS73x4Za4JNGPA8vZqk

Tye, M. (2000). *Consciousness, Color, and Content*. Cambridge: MIT Press.

Wang, Y. (2003). On Cognitive Informatics. *Brain and Mind*, *4*(2), 151–167. https://doi.org/10.1023/A:1025401527570

Wang, Y. (2007). The Theoretical Framework of Cognitive Informatics. *International Journal of Cognitive Informatics and Natural Intelligence (IJCINI)*, *1*(1), 1–27. https://doi.org/10.4018/jcini.2007010101

Wang, Y. (2009). Formal description of the cognitive process of memorization. *Lecture Notes in Computer Science (Including Subseries Lecture Notes in Artificial Intelligence and*

Lecture Notes in Bioinformatics), 5540 LNCS, 81–98. https://doi.org/10.1007/978-3-642-02097-1_5

Wang, Y. (2012a). Cognitive Mechanisms and Formal Models of Consciousness Article in International Journal of Cognitive Informatics and Natural Intelligence. *International Journal of Cognitive Informatics and Natural Intelligence*, *6*(2), 23–40. https://doi.org/10.4018/jcini.2012040102

Wang, Y. (2012b). On abstract intelligence and brain informatics: Mapping the cognitive functions onto the neural architectures. *2012 IEEE 11th International Conference on Cognitive Informatics and Cognitive Computing*, 5–6. https://doi.org/10.1109/ICCI-CC.2012.6311158

Wang, Y., Wang, Y., Patel, S., & Patel, D. (2006). A layered reference model of the brain (LRMB). *IEEE Transactions on Systems, Man and Cybernetics Part C: Applications and Reviews*, *36*(2), 124–133. https://doi.org/10.1109/TSMCC.2006.871126

Ward, L. M. (2011, June). The thalamic dynamic core theory of conscious experience. *Consciousness and Cognition*, Vol. 20, pp. 464–486. https://doi.org/10.1016/j.concog.2011.01.007

Wegner, D. (2002). *The illusion of conscious will*. Cambridge: The MIT Press.

Widerker, D., & Frankfurt, H. (2018). Alternate Possibilities and Moral Responsibility. In *Moral Responsibility and Alternative Possibilities*. https://doi.org/10.4324/9781315248660-2

Yoshimi, J. (2011). Phenomenology and connectionism. *Frontiers in Psychology*. https://doi.org/10.3389/fpsyg.2011.00288

Yoshimi, J., & Vinson, D. W. (2015). Extending Gurwitsch's field theory of consciousness. *Consciousness and Cognition*. https://doi.org/10.1016/j.concog.2015.03.017

Yu, L., & Blumenfeld, H. (2009). Theories of impaired consciousness in epilepsy. *Annals of the New York Academy of Sciences*, *1157*, 48–60. https://doi.org/10.1111/j.1749-6632.2009.04472.x [doi]
